# Genetic history of Cambridgeshire before and after the Black Death

**DOI:** 10.1126/sciadv.adi5903

**Published:** 2024-01-17

**Authors:** Ruoyun Hui, Christiana L. Scheib, Eugenia D’Atanasio, Sarah A. Inskip, Craig Cessford, Simone A. Biagini, Anthony W. Wohns, Muhammad Q.A. Ali, Samuel J. Griffith, Anu Solnik, Helja Niinemäe, Xiangyu Jack Ge, Alice K. Rose, Owyn Beneker, Tamsin C. O’Connell, John E. Robb, Toomas Kivisild

**Affiliations:** ^1^ Alan Turing Institute, London, UK.; ^2^ McDonald Institute for Archaeological Research, University of Cambridge, Cambridge, UK.; ^3^ Estonian Biocentre, Institute of Genomics, University of Tartu, Tartu, Estonia.; ^4^ St John’s College, University of Cambridge, Cambridge, UK.; ^5^ Institute of Molecular Biology and Pathology, CNR, Rome, Italy.; ^6^ School of Archaeology and Ancient History, University of Leicester, Leicester, UK.; ^7^ Cambridge Archaeological Unit, Department of Archaeology, University of Cambridge, Cambridge, UK.; ^8^ Department of Human Genetics, KU Leuven, Leuven, Belgium.; ^9^ School of Medicine, Stanford University, Stanford, CA, USA.; ^10^ Department of Genetics and Biology, Stanford University, Stanford, CA, USA.; ^11^ Core Facility, Institute of Genomics, University of Tartu, Tartu, Estonia.; ^12^ Wellcome Genome Campus, Wellcome Sanger Institute, Hinxton, UK.; ^13^ Department of Archaeology, University of Durham, Durham, UK.; ^14^ Department of Archaeology, University of Cambridge, Cambridge, UK.

## Abstract

The extent of the devastation of the Black Death pandemic (1346–1353) on European populations is known from documentary sources and its bacterial source illuminated by studies of ancient pathogen DNA. What has remained less understood is the effect of the pandemic on human mobility and genetic diversity at the local scale. Here, we report 275 ancient genomes, including 109 with coverage >0.1×, from later medieval and postmedieval Cambridgeshire of individuals buried before and after the Black Death. Consistent with the function of the institutions, we found a lack of close relatives among the friars and the inmates of the hospital in contrast to their abundance in general urban and rural parish communities. While we detect long-term shifts in local genetic ancestry in Cambridgeshire, we find no evidence of major changes in genetic ancestry nor higher differentiation of immune loci between cohorts living before and after the Black Death.

## Teaser

The Black Death had less genetic impact on the medieval residents of Cambridge than local long-term ancestry changes.

## INTRODUCTION

Evidence from ancient DNA (aDNA) continues to increase our understanding of the human past. By linking the genetic profiles to a place and time, it allows us to study population movements (*1*, *2*), genetic relatedness (*3*–*5*), infectious diseases (*6*, *7*), and natural selection (*8*, *9*) as they occurred. When combined with historical and archaeological contexts, such information offers a more detailed perspective on life in past societies.

**Fig. 1.**
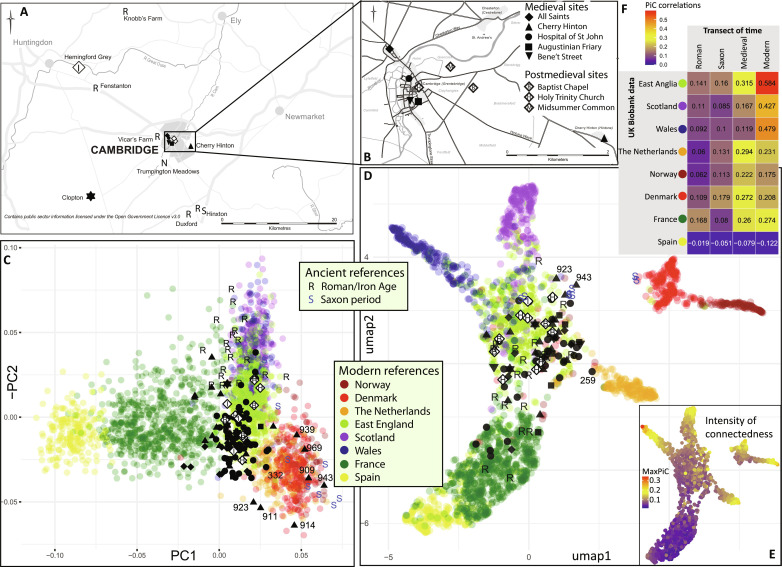
Sampling locations and genetic ancestry. (**A**) Sampling locations in Cambridgeshire. (**B**) Zoomed in map of Cambridge. (**C**) PC plot of 109 later medieval and postmedieval genomes with coverage >0.1× in context of ancient (Roman and Saxon period) and modern (UK Biobank) references, with PC1 and PC2 accounting for 0.165 and 0.07% of total variance explained, respectively. (**D**) Supervised Uniform Manifold Approximation and Projection (UMAP) cluster analysis using PiC with modern references based on 5-cM LSAI sharing among modern and 108 ancient genomes, including 80 from later medieval period, with coverage >0.2×. (**E**) Intensity of maximum PiC scores. (**F**) Transect of time of correlations between the regional PiC vectors. The “Modern” correlation for East Anglia is shown as the correlation between PiC vectors of East Anglia and Bedfordshire/Hertfordshire.

aDNA studies centered around broad geographical regions and long time periods have been fundamental in establishing major migration events, population turnovers, or continuity in both prehistory and historic periods, while being less informative about everyday life experience within complex societies. Taking what we call “the whole town approach,” we have studied hundreds of skeletal remains from later medieval (*c*. 1000–1550 CE) Cambridgeshire (Table 1). They were excavated from burial grounds connected with different social groups: urban and rural parish churchyards, urban charitable institutions, and religious institutions. For historical context, we also included postmedieval burial grounds (*c*. 1550–1855 CE). Apart from Clopton and Hemingford Grey, all the sites are within a few kilometers of each other (Fig. 1A).

**Table 1.** Ancient genomes from later medieval and postmedieval Cambridgeshire. Note: *N*, total number of genomes sequenced per site and by coverage, followed by the numbers assigned to pre– and post–Black Death if the site was in use around the mid-14th century. The pre– and post–Black Death labels are estimated according to whether the burial dates to before or after the year 1348. Some medieval individuals straddle this division and cannot be assigned.

Archaeological site	Social group	Period, date range	*N* (pre–/post–Black Death)	**>** 0.01**×**	**>** 0.05**×**	0.1**×**
Cherry Hinton	Rural parish	Later medieval, 940–1170	48 (48/0)	37	29	25
All Saints	Urban parish	Later medieval, 940–1365	49 (27/1)	34	22	13
Clopton	Rural parish	Later medieval, 1200–1561	17 (0/8)	7	4	4
Hospital of St John	Urban charitable inmates	Later medieval, 1204–1511	104 (36/46)	74	59	45
Augustinian Friary	Urban friars and lay patrons	Later medieval, 1290–1538	28 (17/10)	19	13	7
Bene’t Street	Urban plague pit	Later medieval, 1349	4 (0/4)	2	2	2
Midsummer Common	Urban plague victims	Postmedieval, 1550–1666	2 (0/2)	1	1	0
Hemingford Grey Quakers	Rural noncon formist	Postmedieval, 1681–1721	7 (0/7)	3	3	3
Providence Calvinistic Baptist Chapel	Urban noncon formist	Postmedieval, 1833–1837	6 (0/6)	5	4	4
Holy Trinity Church	Urban parish	Postmedieval, 1833–1855	10 (0/10)	8	6	6
		Total	275 (128/94)	190	143	109

Cambridge during the later medieval period was a middle-sized market town where people from all sections of society crossed paths. After they died, most townspeople were buried in one of the parish cemeteries, including All Saints by the Castle; however, other places of burial existed and increased over time. Toward the end of the 12th century, the Hospital of St John the Evangelist was founded by the townspeople as a charitable institute for the poor, the infirm and the sick. Most of the burials included in this study come from a cemetery for charitable inmates of the Hospital. The 13th century saw the founding of the university and houses of the mendicant orders, including an Augustinian Friary (from which some of our study population are drawn). Apart from the friars, some patrons of the friary were also buried in the cemetery and chapter house of the friary. The first wave of the Second Plague Pandemic (which we hereafter refer to with the commonly used term “Black Death,” although the term was not in use until the 18th century) hit Cambridge in 1349; some of its victims were found in a mass burial of unknown size on Bene’t Street (*10*). Two parish cemeteries outside Cambridge, Cherry Hinton and Clopton, are within the rural hinterland of the town. Table 1 and table S1 list the archaeological sites covered in this study, the dating of the burials, and the function of the medieval and postmedieval sites.

The Black Death and subsequent plague outbreaks had multiple effects on medieval society in England. Its death toll in Europe, estimated at 30 to 65% (*11*, *12*), could have posed selective pressure for better resistance to the plague. Genetic adaptations via the innate (*13*) and adaptive immune system (*14*) have been proposed. Although reference bias can pose challenges to detect allele frequency changes due to natural selection (*15*), a study of 206 aDNA extracts from individuals buried in London and Denmark before, during, and after the Black Death revealed enrichment of immune genes among highly differentiated single-nucleotide variants, suggesting major impact of the pandemic in shaping the disease susceptibility of surviving population (*16*).

Besides genetic susceptibility, it is not clear to what extent social identity modified the morbid and mortal effects of the Black Death. Plague mortality appeared to be selective with respect to frailty (*17*, *18*) caused possibly by factors such as malnutrition, impaired immunocompetence, and others affected by social conditions. For example, the Great Famine of 1315–1322 could have severely affected people of low socioeconomic position. In this sense, health inequality between social groups sets the background for understanding the potential for different experiences through the pandemic. As longer-term consequences of the mortality, it has been argued that the Black Death pandemic initiated or accelerated profound socioeconomic changes, such as increased social mobility, improved quality of life of the laboring population, and technological innovations to increase productivity (*19*, *20*). Together with evidence from osteology, isotopes, and the rich context around the burial grounds, we aim to explore to what extent genetic data might aid the construction of a social history, both in relation to the pandemic and regarding the more stable aspects of later medieval life.

## RESULTS

We extracted aDNA and generated whole-genome shotgun sequence data with a mean coverage of 0.228 from a total of 250 later medieval and 25 postmedieval skeletons, retrieving for further analyses 190 genomes at coverage >0.01× (Table 1 and table S1). They form the most extensive bioarchaeological sampling within a focused temporal and geographical range to date. The examined medieval sites represent burials of individuals from different social and cause of death backgrounds, including urban cemeteries of the charitable poor from the Hospital of St. John, All Saints parish cemetery, Augustinian Friary, Bene’t Street plague burial, and rural cemeteries of Cherry Hinton and Clopton (Table 1, Fig. 1A, and the Supplementary Materials). The analyses of the medieval genomes were performed in context of postmedieval genomes from four sites in Cambridgeshire (Table 1) as well as published genomic data of the Late Iron Age/Roman (*c*. 100 BCE to 400 CE) and Early Saxon periods (*c*. 400–700 CE) from Cambridgeshire and elsewhere from England (*21*, *22*). Average endogenous human DNA content was 13% and average contamination rate 1.06%, with 209 individuals under 5%. Average damage in the first 5 base pairs (bp) was 8.02% (table S1). A subset of 143 genomes sequenced to >0.05× coverage were imputed to study the changes in phenotypes related to health and lifestyle. The imputed genomes include 109 individuals with coverage >0.1×, which were subsequently used to resolve genetic ancestry, kinship, recent inbreeding, and heterozygosity.

### Genetic ancestry

The frequencies of mitochondrial DNA (mtDNA) haplogroups in England have remained relatively stable since the Neolithic (table S2). Similarly, principal components analysis (PCA) reveals that all 109 individuals with >0.1× coverage from later medieval Cambridgeshire (Fig. 1, A and B) share their autosomal ancestry with modern northern and western European populations without evidence of migration from more distant regions (Fig. 1C and fig. S1); the same conclusion is supported when projecting pseudohaploid genomes without imputation onto PC space established by modern genomes (figs. S2 to S11). In contrast to genomes from the Roman or Early Saxon periods (*21*, *22*), most later medieval genomes cluster with those from the modern English genomes from the UK Biobank data (Fig. 1C). Individual outliers who, similarly to most Early Saxon period individuals, are placed among modern Dutch and Danish populations, include a few from Cherry Hinton and the Hospital of St John. Two of them (PSN332 from the Hospital and PSN930 from Cherry Hinton) are also outliers in terms of dental enamel ^87^Sr/^86^Sr values (PSN332 = 0.7122, PSN930 = 0.7108) (*23*). These values, particularly for PSN332, are higher than the estimated biosphere ^87^Sr/^86^Sr values for the East of England (*24*), indicating that they did not spend their childhoods in the area local to where they were buried.

To study the genetic affinity changes across time at finer geographic resolution, we defined interindividual connections by identifying long [>5 centimorgan (cM)] shared allele intervals (LSAIs) with IBIS (*25*) and explored the modularity of individual connectedness (PiC) (*26*) among the historical and modern genomes. Similarly to PCA results, we find that the majority of historical genomes from Cambridgeshire cluster by their connectedness with modern UK Biobank genomes from East England (Fig. 1D and table S3) whereas a small fraction of later medieval and Roman period genomes, which display low LSAI sharing with any population (Fig. 1E), cluster with the UK Biobank donors born in France who also display low levels of LSAI sharing. The Early Saxon period genomes show higher connectedness with Scandinavian genomes, which is also reflected in individual PCA outliers from Cherry Hinton. Overall, we observe regional shifts in individual connectedness over time (Fig. 1F). We observe increasing Danish connectedness in the transition from Roman to Early Saxon period; later, during and after the later medieval period, there is an increase of LSAI sharing with both modern Dutch genomes [mirroring documentary evidence showing the Dutch as the most common late medieval immigrants locally (*27*, *28*)] and genomes from a broader zone of England. Last, we identify a major shift in modern East England toward higher LSAI sharing with Wales and Scotland, clearly reflecting the political and economic integration of recent Britain.

**Fig. 2.**
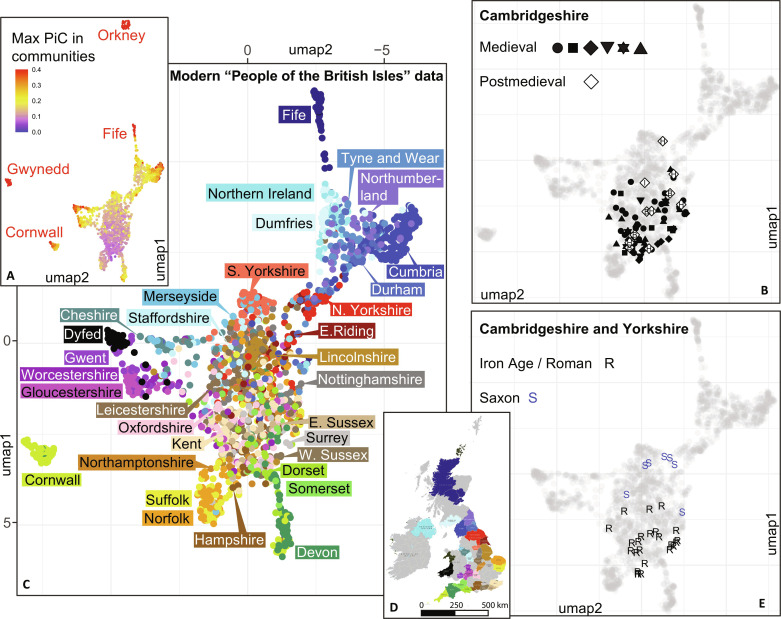
UMAP plot of individual connectedness among modern and ancient genomes from Britain. (**A**) Density of maximum PiC score values per individual in one of the extracted communities. (**B**) UMAP coordinates of the medieval and postmedieval genomes (> 0.2× coverage) from Cambridgeshire. Archaeological site codes as shown in Fig. 1. (**C**) Individual connectedness among modern genomes of the “People of the British Isles” project based on PiC scores of 20 significant communities with more than 10 members extracted from the combined data with the Louvain method (unsupervised cluster analysis). (**D**) Map showing the color codes by counties for the modern genomes used in the UMAP plot A. (**E**) UMAP coordinates of the Iron Age/Roman and Saxon period genomes.

Our analyses of individual connectedness in the People of the British Isles (*29*) data suggest that all later medieval genomes from Cambridgeshire likely draw most of their genetic ancestry broadly from the same sources as present-day central/eastern England population (Fig. 2). Although we are able to distinguish certain regional differences in the modern data with our approach, such as between Cornwall and Devon or between North and South Yorkshire, we observe less resolution in a broad area between Lincolnshire and Surrey where our ancient genomes come from (Fig. 2). This means that even if some of the individuals had come from Kent or Lincolnshire, for example, we would not be able to detect such fine-scale migration patterns among regions within that area.

Changes in genetic ancestry or selective pressure could cause phenotypic changes over time. We analyzed 214 genomes with >0.05× coverage from the Roman period to the 19th century, including previously published data (*21*, *22*, *30*), for changes in allele frequency of 113 phenotype informative single-nucleotide polymorphisms (SNPs) related to diet, health, and pigmentation (tables S4 to S6). Of 74 SNPs related to health and diet, only 2 involved in autoimmune diseases reached the adjusted significance threshold in the analysis of variance (ANOVA) statistical tests, showing differences between the medieval and postmedieval periods. One SNP, i.e., rs6822844, is an intronic variant in the *KIAA1109/Tenr/IL2/IL21* block and has been identified as a risk factor in several autoimmune diseases, including celiac disease, rheumatoid arthritis and type 1 diabetes (*31*, *32*). The other variant, i.e., the intronic rs1891467 in the *TGFB2* gene, has been associated with sarcoidosis (*33*). We did not find significant allele frequency changes during and after the later medieval period for the 39 SNPs affecting eye, hair, and skin color included in the HIrisPlex-S set (*34*), which is expected considering the proximity of the later medieval and present-day English in genetic ancestry (Fig. 2).

### Social landscape

#### Kinship and relatedness

**Fig. 3.**
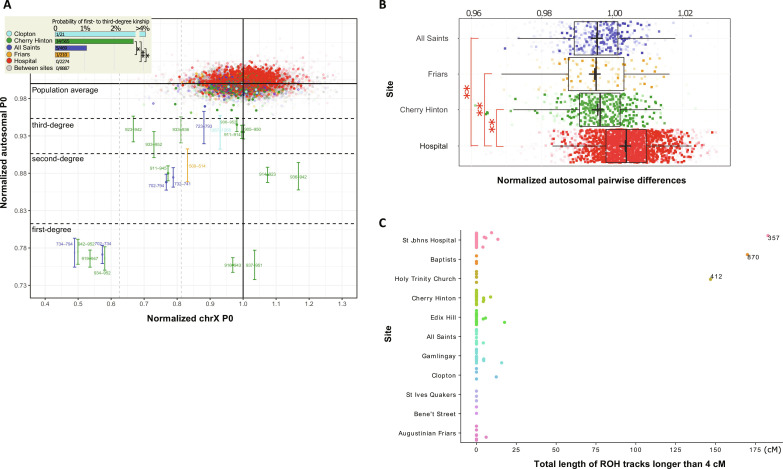
Kinship, genetic diversity and inbreeding. (**A**) Normalized pairwise differences (P0) in autosomal data and X chromosome for later medieval sites with more than five burials. Each individual data point represents a pair of individuals (from a total of 171 individuals with >0.01× coverage tested), the aggregate coverage of which is reflected by the opacity of the color. Boundaries for the first- to third-degree of relatedness for autosomal data were defined as in (*3*). Error bars with two SEMs are shown for the pairs of first- to third-degree of relatedness only. ** and * correspond to significant differences at *P* < 0.01 and *P* < 0.05 by two-tailed *t* test, respectively. (**B**) Boxplot of normalized autosomal pairwise differences in four Cambridge medieval sites represented by the largest sample size in this study. Each rectangular data point represents a pairwise comparison of individuals sampled from the same site, normalized (with READ) by the average of all pairwise comparisons made in the pool of all later medieval individuals from Cambridge. The opacity of the rectangular color reflects the aggregate of the coverages of the two individuals. The results of significant (*P* < 0.01) two-tailed Wilcoxon rank sum test are shown with **. (**C**) The total lengths of ROH tracks longer than 4 cM in individuals grouped by site, showing highly inbred outliers.

Although the “kinship” bonds that tie together social groups often go beyond or replace “blood-relationships,” the types and intensity of genetic relatedness among individuals buried in the same locality can be informative of the social structure of the population. To study the probability of genetic relatedness among burials of different social backgrounds we used READ-based estimates (*3*) of pairwise differences in autosomes and the X chromosome in 171 later medieval genomes with >0.01× coverage. Among individual pairs with >10,000 overlapping SNPs, 21 cases of first- to third-degree relatedness were detected (Fig. 3A and table S7). All kinship pairs detected by READ that involved individuals with >0.1× coverage were confirmed in our analyses with IBIS (*25*) to share multiple > 7-cM LSAI segments and kinship coefficient > 0.005 (table S7). Expectedly, considering the time gaps, none of the 97 later medieval individuals showed kinship with 463,855 modern UK Biobank individuals by the same threshold. Nine of the 12 tested postmedieval individuals were found to form a total of 20 fourth- to sixth-degree relationships with modern individuals who identify themselves as British, including one born outside of the United Kingdom. Among the later medieval individuals, we detect 12 cases of more distant form of relatedness within the same archaeological site beyond those identified with READ—10 at Cherry Hinton and 2 at All Saints—while none were found at the Hospital or Friary. We found multiple cases (more than 1% of all pairs considered) in the rural Cherry Hinton and urban All Saints parish cemeteries. In contrast, we detected only one pair of relatives, a middle-aged (46 to 59 years old) friar and a female child (second degree). No kinship relations were found at the Hospital, despite the large sample size analyzed. On average, pairwise differences between individuals from the Hospital were found to be greater than those between individuals from other sites (Fig. 3B), highlighting the heterogeneity of the ancestry of individuals entering the Hospital.

We also searched for runs of homozygosity (ROH) tracks in the imputed genomes using hapROH (*5*) and found that most individuals have no or very few ROH tracks that are longer than 4 cM (table S8), while three individuals (PSN357 from the Hospital, PSN870 from the Baptist Chapel, and PSN412 from Holy Trinity) have up to 150 to 175 cM in ROH, which is compatible with the parents being fourth- to fifth-degree relatives, including second- to third-cousin marriage (Fig. 3C).

### Before/after the Black Death

Because of the broad error range of the radiocarbon dates as well as the limited accuracy of the assignments to the pre/post–Black Death groups based on associated finds and position in the sequence of dated stratigraphic context, some individuals in the post–Black Death group might have been born before 1348, although two obvious cases from Bene’t Street have been excluded from analysis. This might limit our power to detect changes after the Black Death. None of the individuals in the pre– or post–Black Death groups are among those tested positive for *Yersinia pestis* previously (*10*, *35*, *36*). Our analyses of genetic ancestry (Figs. 1 and 2, figs. S1 to S11, and table S2) were unable to detect changes in rates of long-distance migration associated with the Black Death comparable to those recently shown in case of Trondheim population (*15*). However, besides the potential effect on broader regional ancestry, the Black Death pandemic could have left other detectable signatures on genetic diversity of the population at the genome scale, or, it could have affected specific genes and variants associated with infectious disease vulnerability. To examine its effect on the genetic diversity of the Cambridge medieval population further, we estimated heterozygosity and nucleotide diversity genome-wide and in the human leukocyte antigen (HLA) locus in imputed genomes.

**Fig. 4.**
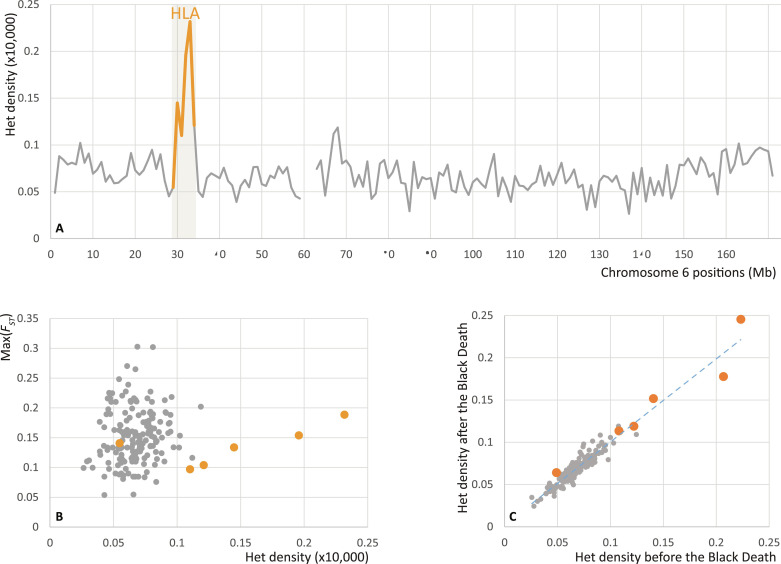
Heterozygote density and allele frequency differentiation in the HLA locus. (**A**) Distribution of average heterozygote density in 1-Mbp windows of chromosome 6. Gray line shows the density of heterozygous sites at common variant positions with minor allele frequency higher than 0.05 in the HRC imputation panel in chromosome 6 for 50 imputed (>0.1× coverage) pre– and post–Black Death genomes from Cambridge. The orange line highlights windows containing genes in the HLA locus. (**B**) Scatter plot of Max(*F_ST_*) - maximum *F_ST_* between before and after the Black Death cohorts - and Het density values by 1-Mbp windows of chromosome 6. (**C**) Het density in the before (*n* = 31) and after (*n* = 19) the Black Death cohorts.

Genome-wide heterozygosity and nucleotide diversity are sensitive to demographic events such as bottlenecks, founder events, or admixture. High mortality during the pandemic could be detectable in extreme cases in a small isolated population as a reduction of diversity across all loci. Changes in the HLA region might capture possible signals of selection specifically at immunity loci. If any one or a few variants in this locus responded to selection, they would have been expected to affect the whole region because of linkage. Consistent with the long-term effect of balancing selection on HLA locus, we find this region has higher density of heterozygous positions at common variants and nucleotide diversity in our pool of imputed genomes (Fig. 4A and fig. S13). However, the “before” and “after” the Black Death cohorts do not show higher than average allele frequency differentiation within the HLA region (Fig. 4B) nor notable differences in the heterozygote density (Fig. 4C). Within a subset of 50 imputed genomes assigned to either before or after the Black Death and coverage >0.1×, we observed no significant differences in genome-wide (two-tailed *t* test, *P* = 0.205) nor HLA locus (*P* = 0.700) heterozygosities (fig. S12). Similarly, we did not detect changes in nucleotide diversity in the HLA region or genome-wide after the Black Death (fig. S13).

Our analyses of 70 pre– and post–Black Death imputed genomes for changes in allele frequency in 25 variants previously identified as potential targets of selection in humans against viral and bacterial pathogens as well as four variants recently highlighted as selection targets specifically against *Y. pestis* (*16*) revealed (tables S4 and S6) one significant (*P* = 0.003) difference at individual test level at the rs42490 SNP in the *RIPK2* gene. The *RIPK2* allele previously shown to be protective against leprosy (*37*) showed increased allele frequency after the Black Death. This result would not remain, however, significant after applying multiple test corrections and considering the limited sample size of our cohort it requires further validation in an independent dataset.

None of the four immunity variants identified by Klunk *et al.* (*16*) with significant allele frequency changes both in their London and Danish cohorts were replicated between Cambridge before and after Black Death cohorts (table S9). Using simulations, we demonstrated that this is unlikely to be caused by a lack of power due to our sample size (fig. S14). We did observe a similar enrichment (1.4-fold, *P* = 0.0001) of variants related to immunity among highly differentiated variants (*F_ST_* > 95th percentile) when using the same list of immunity-related and neutrally evolving variants as the authors (tables S9 and S10). Of the 245 highly differentiated immunity-related variants identified in their London cohort, 22 were replicated, significantly more than expected by chance (*P* = 0.0001); However, 10 of the 22 overlapping variants that are above the 95th threshold in the Cambridge cohort and 2 of the 3 variants above the 99th threshold show opposite directionality of allele frequency change in time in London and Cambridge cohorts (table S9). While the minor allele frequencies of the immunity variants appear to be highly correlated between our studies (*r* = 0.90, *P* < 1 × 10^−15^), the *F_ST_* between the pre- and postpandemic cohorts are not (*r* = 0.019, *P* = 0.26). The significant enrichment of immunity genes cannot be reproduced with our data when using the full list of 37,574 neutral regions defined in (*38*) instead of the relatively small number of variants ascertained by Klunk *et al. (*16**) in its subset of 250 regions (table S10). We observe a reduction (1.14-fold, *P* = 0.29) of high *F_ST_* values among the Klunk *et al. (*16**) immunity variants when we define the neutral 95th threshold using 55,965 variants from the full range of the 37,574 neutral regions, which becomes significant (1.73-fold, *P* < 1 × 10^−10^) when also using an expanded set of immunity variants from InnateDB (table S10). Notably, within the pool of highly differentiated immune locus variants identified by Klunk *et al. (*16**), we observe significant excess of “gwas” variants, i.e., positions that had previously been confirmed to be polymorphic (table S10) over immunity variants ascertained in the exonic regions, suggesting that ascertainment of previously unidentified variants from low-coverage data (as by Klunk *et al. (*16**) in their “exon” and “neutral” categories) is one possible cause for the disappearance of the signal when we used the full set of neutral regions defined in (*38*) that overlap with positions confirmed to be polymorphic in the Haplotype Reference Consortium (HRC) panel (*39*) to define the threshold.

## DISCUSSION

Our analyses of genetic ancestry in Cambridge through the transect of time revealed, first, notable differences between individuals from different time periods in their placement on PCA and a corresponding increase of LSAIs with continental North Europe between the Roman and more recent periods. These findings partly reflect the cumulatively massive changes in local genetic ancestry in East England that occurred over many centuries in the first millennium CE (*22*, *40*). Most of the individuals that fall outside of the local range of variation come from the rural cemetery at Cherry Hinton, possibly reflecting a diversity of Late Anglo-Saxon, Scandinavian, and Norman ancestries in the process of intermixing. Despite the scale of these ancestry changes, it is difficult to convincingly detect any first-generation migrants from other parts of western Europe using genetic information alone, partially becuse of an absence of comparable reference material from medieval Europe. In case of the Friary, a context where individuals with nonlocal ancestry would have been more likely to be found, it is also possible that some migrating friars living in Cambridge during their life moved back and were buried elsewhere. Second, with the analyses of individual connectedness patterns, we were able to distinguish between modern Dutch, Danish, and Norwegian ancestries and observed a major increase of population-scale connectedness in Cambridge with the Dutch dating to the later medieval period. This pattern could, in principle, reflect gene flow either from the Low Countries to Cambridge or gene flow from East England to the Low Countries, although the former is better supported by documented sources. Last, the major change we observed between medieval and modern periods in connectedness with modern Welsh and Scottish genomes is likely to reflect recent and ongoing migrations and mobility. In contrast to the highly region-specific LSAI sharing between modern and medieval genomes in Estonia (*26*), later medieval Cambridge genomes do not show increased affinity to modern East Anglia compared to other regions. These recent changes in genetic ancestry suggest that modern Biobank sources may not be ideal references for the study of population histories, highlighting the need for aDNA sampling of historic populations from different time points.

Although we observe major long-term changes in genetic ancestry, we were unable to detect a change in ancestry or genetic diversity between our pre– and post–Black Death cohorts to account for the hypothesized increased mobility after the Black Death caused by labor shortages. The short time span of a few decades, small sample size and relatively low genetic differentiation in central and eastern England [Fig. 2; (*29*)] would all make detecting short- to medium-range migration within England challenging. The lack of evidence for long-distance migration is in agreement with earlier mtDNA evidence in medieval British and Danish cohorts (*41*) but in contrast to genome-wide evidence of increased long-distance migration from medieval Trondheim (*15*). A recent isotope study also found that fewer adolescents moved into York from long distance following the Black Death, possibly because of the economic decline of York and the enforcement of labor laws (*42*). These results suggest that the pandemic had a variable impact on human mobility in European cities depending on their regional, socioeconomic, and political context and circumstances.

We found that all later medieval social groups we examined—the ordinary urban population buried at All Saints, rural population buried at Cherry Hinton, charitable inmates buried at the Hospital of St John, and friars and benefactors buried at Augustinian Friary—shared largely the same local genetic ancestry. Although historical record suggests that some friars might have traveled from as far as the Mediterranean to attend the Augustinian studium generale (regional study center) they were either not sampled or buried elsewhere. In contrast to the lack of differentiation by ancestry, we found clear differences in kinship probabilities between the social groups. The lack of close genetic kinship is consistent with the function of the Hospital as a safety net for those without family support, although we note that genetic relatedness does not capture the whole range of social relationships viewed as kinship. Relatedly, the hospital inmates also show elevated background genetic diversity compared to other later medieval sites. Some of them could have arrived from farther away than the average townsfolk, and the lack of local social support might be related to their hardship. Given that friaries maintained long-lasting ties with local benefactor families, it is not unexpected that a female child who was a second-degree relative to one of the friars was allowed to be buried within the Friary, but no other close genetic relatives were found among the Friary population.

We found a small number of cases of extended ROH tracks in the genome consistent with the parents being second to third cousins. These cases can be considered unexpected because marriage within four degrees of consanguinity was forbidden by the Church (*43*). We speculate that this could result from incomplete church records, especially if the two parents attended different parish churches, or extra-pair paternity events in the family history.

The Black Death has been hypothesized to exert selective pressure on genes related to health and immunity (*14*, *44*). If existing genetic variations differ in their resistance against the plague, and if the plague was a major cause of mortality, we would expect a drop in genetic diversity immediately after the pandemic. However, among individuals who lived shortly before or after the Black Death, we did not find changes in heterozygosity in the HLA region. Although the mortality rate (30 to 65%) was devastating, followed by subsequent plague outbreaks until the second half of the 17th century in Britain, it may not have exerted a strong enough uniform selective pressure for a long enough period to leave a detectable signature in the low-coverage genomes. It should be cautioned, though, that conclusions drawn from HLA analyses at SNP and haplotype level may differ (*45*). Immel *et al.* (*14*) identified frequency differences in three HLA haplotypes between 16th-century plague victims in Ellwangen, Germany and the modern local population that were significant at the individual test level (*P* < 0.05) while failing to pass the significance test after multiple test correction. While the signal of heterozygosity change has been recently used to map an ancient selective sweep in the HLA region (*46*), the fact that we were unable to detect significant changes in HLA heterozygosity in our data may be a reflection of the short effect time of the pandemic.

Similarly, we did not find an excess of variants in genes related to health and immunity to be highly differentiated between the before and after the Black Death cohorts. The most highly differentiated variant in the *RIPK2* gene shows an increase in frequency after the Black Death of an allele protective against leprosy. Notably, this gene has been suggested to also have a role in the recognition of *Y. pestis* by the innate immune response (*47*). Some scholars have suggested that the Second Plague Pandemic contributed to the decline of leprosy starting from the mid-13th century, as many people vulnerable to leprosy fell victim to the plague (*48*, *49*). On the other hand, we could not replicate in our Cambridge cohort the findings by Klunk *et al.* (*16*) of significantly higher differentiation of immune genes, and, more specifically, differentiation of the four SNPs identified in their London and Danish cohorts (table S4). The signal of enrichment disappears or even reverses direction when we use an extended set of neutral regions as our reference. The high correlation in allele frequency of common variants [minor allele frequency (MAF) > 0.1 in Klunk *et al. (*16**) data] between our Cambridge cohort and the London/Danish cohort and the fact that we got qualitatively similar results when using the same variant lists suggest that differences in genotyping, either with or without imputation, are not likely the key factors; our results show that the subset of 250 regions used by Klunk *et al. (*16**) are likely to contain too few variants to be robustly representative of variation in the full list of 37,574 neutral regions defined by Gronau *et al.* (*38*). Furthermore, Barton *et al.* (*50*) have shown that the enrichment of high *F_ST_* variants at immune loci in Klunk *et al. (*16**) results is attributable to a statistical artifact caused by different coverage of loci. Although not supporting the results of Klunk *et al. (*16**) study, our results are in line with other research showing relatively low or moderate differentiation of immunity genes by *F_ST_* (*51*, *52*). Low differentiation is consistent with the expectations of the theory of balancing selection (*45*) as well as poison/antidote model (*53*) whereby infectious disease transmission between communities often co-occurs with mobility and gene flow that locally increase the effective population size of genes involved in host defense. Because immunity genes appear to show generally lower *F_ST_* values than the rest of the genome, our observation of lower differentiation between the pre– and post–Black Death cohorts does not by itself reflect negative selection at the given time point.

Our limited sample size sets restrictions to identification of selection, yet it is comparable to the one used by Klunk *et al.* (*16*), and although low sequencing coverage could prevent us from identifying individual loci under selection, the polygenic approach taken by Klunk *et al. (*16**), which we have followed, should in principle be able to detect selection at many variants contributing to a trait. Although our results revealed no enrichment of highly differentiated variants in immunity genes between the before and after the Black Death cohorts, these results do not mean that plague had no selective impact on genetic variation in Cambridge. The immune response to *Y. pestis* might involve multiple pathways which are yet to be fully understood and by our “blind” approach of including all possible immune variants the signal of selection can be buried under the background of allele frequency changes of many loci that are not responsive to plague.

In sum, we have shown that even at very low coverage, whole-genome sequencing of historical genomes when combined with rich historical context and other archaeological evidence can help to reconstruct many aspects of medieval life: the occasional incomers in a rural community; a young child from a benefactor family who was buried in the Friary; the Hospital full of unrelated inmates who might have traveled from afar; the social groups that had little difference in genetic background; a pandemic whose genetic consequence in the Cambridge population remained evasive, despite the devastating effect on individual lives and medieval society. The range will surely expand with advancement in aDNA sequencing technology as well as framework to combine various sources of evidence.

## MATERIALS AND METHODS

### Sample information and ethical statement

A description of the archaeological sites included in this study can be found in the Supplementary Materials. All skeletal elements were sampled with permissions from the representative bodies/host institutions. Samples were taken and processed to maximize research value and minimize destructive sampling. Teeth were sampled from skeletons using gloves. Molars were preferred because of having more roots and larger mass, but premolars were also sampled.

### Dating

The radiocarbon determinations were undertaken at the Scottish Universities Environmental Research Centre radiocarbon laboratory and followed their standard procedures (*54*). Analysis was undertaken using OxCal v.4.3 (*55*, *56*) using the IntCal13 calibration curve (*57*). A small number of additional radiocarbon determinations (marked by * in table S1) were undertaken at a late stage, to date specific individuals that produced evidence for specific pathogen aDNA. These were undertaken at the 14Chrono Centre, Queen’s University Belfast. These additional determinations were calibrated using IntCal 2020 (*58*).

At the Hospital of St John, Augustinian Friary and All Saints by the Castle, detailed Bayesian modeling of these results combined with expert archaeological judgment has helped to inform whether these individuals (along with others in the stratigraphic sequence) died before or after the Black Death pandemic of 1348–1349. For skeletons for which direct radiometric dates were not available, dating was based on associated finds and relative position within the sequence of dated stratigraphic contexts within a site.

### Sampling, aDNA extraction, and library preparation

Inside a class IIB hood in the dedicated aDNA facility of the University of Cambridge Department of Archaeology or University of Tartu Institute of Genomics, root portions of teeth were removed with a sterile drill wheel. Petrous was sampled with a 10-mm core drill sterilized with bleach followed by distilled water and then ethanol rinse. In some cases, postcranial remains were also sampled (see table S1); in these cases, a small portion was cut off with a sterilized drill wheel and treated like a tooth root/petrous portion except that proteinase K was not added to the extraction buffer. Root and petrous portions were briefly brushed to remove surface dirt, any varnish or lacquer, and microbial film with full-strength household bleach (6% w/v NaOCl) using a disposable toothbrush that was soaked in 6% (w/v) bleach before use. They were then soaked in 6% (w/v) bleach for 5 min. Samples were rinsed twice with 18.2-megohm·cm H_2_O and soaked in 70% (v/v) ethanol for 2 min, transferred to a clean paper towel on a rack inside a class IIB hood with the ultraviolet light on, and allowed to dry. They were weighed and transferred to polymerase chain reaction (PCR)–clean 5- or 15-ml conical tubes (Eppendorf) for chemical extraction.

Inside a class IIB hood, per 100 mg of each sample, 2 ml of 0.5 M EDTA buffer (pH 8.0) (Fluka) and 50 μl of proteinase K (10 mg/ml; Roche) was added. Tubes were rocked in an incubator for 72 hours at room temperature. Extracts were concentrated to 250 μl using Amplicon Ultra-15 concentrators with a 30-kDa filter (Millipore). Samples were purified according to the manufacturer’s instructions using buffers from the Minelute PCR Purification Kit (Qiagen) with the following changes: (i) the use of High-Volume spin columns (Roche); (ii) 10× PB buffer instead of 5×; and (iii) samples incubated with EB buffer (Qiagen) at 37°C for 10 min before elution. The columns were transferred to clean, labeled, 1.5-ml Eppendorf tubes. One hundred microliters EB buffer is added to the membrane and centrifuged at 13,000 rpm for 2 min after the 10-min incubation and stored at −20°C. Only one extraction was performed per sample for screening and 30 μl used for libraries.

Library preparation was conducted using a protocol modified from the manufacturer’s instructions included in the NEBNext Library Preparation Kit for 454 (E6070S, New England Biolabs, Ipswich, MA) as detailed in (*59*). DNA was not fragmented and reactions were scaled to half volume; adaptors were made as described in (*59*) and used in a final concentration of 2.5 μM each. DNA was purified on MinElute columns (Qiagen). Libraries were amplified using the following PCR setup: 50 μl of DNA library, 1× PCR buffer, 2.5 mM MgCl_2_, BSA (1 mg/ml), 0.2 μM inPE1.0, 0.2 mM deoxynucleotide triphosphate (dNTP) each, HGS Taq Diamond (0.1 U/μl), and 0.2 μM indexing primer. Cycling conditions were as follows: 5′ at 94°C, followed by 18 cycles of 30 s each at 94°, 60°, and 68°C, with a final extension of 7 min at 72°C. Amplified products were purified using MinElute columns and eluted in 35 μl of EB (Qiagen). Three verification steps were implemented to make sure library preparation was successful and to measure the concentration of double-stranded DNA/sequencing libraries—fluorometric quantitation (Qubit, Thermo Fisher Scientific), parallel capillary electrophoresis (Fragment Analyser, Advanced Analytical), and quantitative PCR.

### DNA sequencing

DNA was sequenced using the Illumina NextSeq 500/550 High-Output single-end 75 cycle kit at the University of Cambridge Department of Biochemistry DNA Sequencing Facility. As a norm, 20 samples were sequenced together on one flow cell; additional data were generated for samples over 5% endogenous human content to increase coverage.

### Mapping

Before mapping, the sequences of the adapters, indexes, and poly-G tails were removed from read ends and reads shorter than 30 bp were removed using cutadapt-1.11 (*60*).

The sequences were aligned to the reference sequence GRCh37 (hg19) using Burrows-Wheeler Aligner (BWA 0.7.12) (*61*) and the command *aln* with reseeding disabled.

After alignment, the sequences were converted to BAM format and only sequences that mapped to the human genome were kept with samtools 1.3 (*62*). Data from different flow cell lanes were merged and duplicates were removed using picard 2.12.0 (http://broadinstitute.github.io/picard/index.html, accessed in September 2017). Sequencing coverage was estimated using Qualimap 2.2.1 (*63*) after applying a custom accessibility mask that includes the 1000 genomes accessibility mask and a composite mappability track (*64*).

### aDNA authentication

As a result of degradation over time, aDNA can be distinguished from modern DNA by certain characteristics: short fragments and a high frequency of C > T substitutions at the 5′ ends of sequences due to cytosine deamination. The program mapDamage2.0 (*65*) was used to estimate the frequency of 5’ C > T transitions. Rates of contamination were estimated from mtDNA using ContamMix (*66*) and from X chromosome using two methods implemented in ANGSD (*67*).

Samtools 1.3 (*62*) option stats was used to determine the number of final reads, average read length, average coverage, etc. The average endogenous DNA content (proportion of reads mapping to the human genome) was 13.22% (0.00 to 81.27%).

### Calculating genetic sex estimation

Genetic sex was calculated using the methods and script described in (*68*), estimating the fraction of reads mapping to Y chromosome out of all reads mapping to either X or Y chromosome. Genetic sex was calculated for genomess with a coverage >0.01×, and only reads with a mapping quality >30 were counted for the autosomal, X, and Y chromosomes.

### Determining mtDNA haplogroups

mtDNA haplogroups were determined using Haplogrep2 (*69*). Subsequently, the identical results between the individuals were checked visually by aligning mapped reads to the reference sequence using samtools-1.3 (*62*) command tview and confirming the haplogroup assignment in PhyloTree (accessed at: www.phylotree.org). In addition, private mutations were noted for further kinship analysis.

### Y chromosome variant calling and haplotyping

A total of 256,463 binary Y chromosome SNPs that have been detected as polymorphic in previous high-coverage whole Y chromosome sequencing studies (*70*–*72*) and by YFull (www.yfull.com/snp-list/) were called in male individuals with more than 0.001× autosomal coverage using ANGSD-0.916 (*67*) “-doHaploCall” option. Basal haplogroup affiliations (table S1) could be determined for 128 individuals by assessing the proportion of derived allele calls (pD) in a set of primary (A, B, C...T) haplogroup defining internal branches, as defined in (*71*), using 4081 informative sites. Haplogroup assignments were confirmed and specified using pathPhynder (*73*). Further detailed subhaplogroup assignments within the phylogeny of the primary haplogroup were determined on the basis of mapping the derived allele calls to the internal branches of the YFull tee (www.yfull.com/tree/), requiring support of at least two variants for the terminal branch assignment.

### Pseudohaploid genotype calling

Autosomal variants were called with the ANGSD 0.917 (*67*) command --doHaploCall, which randomly selects one base at each specified position in the genome. The pseudohaploid genotypes were used in genetic kinship analysis with READ and for PC analyses of individual sites with smartPCA (figs. S2 to S11).

### Imputation

Following (*74*), genotype likelihoods were first updated with Beagle 4.1 (*75*) from genotype likelihoods produced by ANGSD 0.917 (−doMajorMinor 3 -GL 1 -doPost 1 -doVcf 1 -sites …) (*67*) in Beagle -gl mode, followed by imputation in Beagle -gt mode with Beagle 5 (*76*) from sites where the genotype probability (GP) of the most likely genotype reaches 0.99. To balance between imputation time and accuracy, we used 503 Europeans genomes in 1000 Genomes Project Phase 3 (*77*) as the reference panel in Beagle -gl step, and 27,165 genomes (except for chromosome 1, where the sample size is reduced to 22,691 because of a processing issue in the release) from the HRC (*39*) in the Beagle -gt step. After filtering out rare variants with MAC <5, we retained a total of ~37 million variants genome-wide. Because Beagle treats “./.” in the variant call format (VCF) input as sporadically missing and imputes them during haplotype phasing, which damages the accuracy when such missing genotypes are common, we imputed each genome individually so that missing genotypes were not included in the VCF input to Beagle 5. The output single-individual VCFs were then merged for downstream analysis. Apart from newly generated genomes in this study, we also imputed published genomes from (*21*, *22*, *30*).

We down-sampled a 10.83× genome that has been previously reported in (*78*), PSN31, to 0.05× to 1× to evaluate our imputation pipeline (table S11). The accuracy was estimated by comparing the imputed genotypes to genotypes called using GATK HaplotypeCaller (*79*) (with the flags --min-pruning 1 --min-dangling-branch-length 1).

For some analysis, we filtered the imputed genotypes further by GP and/or minor allele frequencies at the sites as described in the respective sections. In general we applied a coverage cutoff of at least 0.1× for analyses requiring individual genotypes and lower cutoffs where allele frequency is of more concern. We also sought to address the questions using both imputed and genotyped data whenever possible.

### Principal components analysis

We used FlashPCA2 (*80*) for PCA of imputed genomes (without projection) together with modern reference genomes from selected groups in UK Biobank after excluding variants in linkage disequilibrium with the PLINK --indep-pairwise 1000 50 0.5 option and exclusion of the likely non-neutral regions exclusion_regions_hg19.txt (Fig. 1C and fig. S1). To check for possible artifacts introduced by imputation, we also performed PCA using modern reference genomes only, before projecting pseudo-haploid genomes (coverage >0.05×) onto the PC space following (https://github.com/chrchang/eigensoft/blob/master/POPGEN/lsqproject.pdf, accessed in April 2020) (figs. S2 to S11). The error bars of the projected genomes represent 1 SD obtained from a 20-fold block jackknife. The results using imputed genomes and pseudohaploid genotypes are highly consistent with largely overlapping outliers (apart from those only included in PCA with lsq projection due to low coverage).

### Detecting IBD and LSAI segments

Identical by descent (IBD)/LSAI segments (*26*) and kinship coefficients were estimated from merged plink files of 80 (coverage >0.2×) imputed ancient genomes, 503 Europeans from the 1000 Genome Project and UK Biobank data with IBIS version 1.20.9 (*25*) using minimum shared segment length (−min_L) thresholds 5 and/or 7 cM together with -maxDist 0.1 and -mt 300 parameters. In total, 269,319 binary SNPs with MAF > 0.05 were used.

Although IBIS has the highest IBD inference accuracy for >7-cM segments, which we use in kinship analyses, we use >5-cM threshold in our diachronic inferences of population affinities because our focus is on relationships at generational distances >15 at which longer IBD sharing expectations become relatively low, particularly in combination with the loss of sensitivity to detect long IBD segments from imputed aDNA sequences. Because true IBD segments of this length are not expected to be common at these generational distances, we need to consider the detected segments as “long shared allele intervals” (LSAIs) rather than IBD segments sensu stricto. Because they are inferred from unphased data after removal of rare variants (which cannot be imputed with sufficient accuracy), the LSAIs are likely to include undetected recombination points and smaller IBD segments residing on different haplotypes.

The PiC score (*26*) for individual x in group Z was estimated as the proportion of individuals from group Z with whom individual x shared IBD above the given threshold. In practice, we estimated the count of connected individuals from group Z from sorted IBIS .coef output files by using the linux “join” function to add group codes to individual identifiers and by using the “crosstab” function of datamash (*81*) to generate the table of counts, each of which we divided by the total number of individuals in group Z to obtain the individual connectedness proportions by groups (the PiC scores).

Unsupervised community extraction analyses on IBIS .coef files calculated from merged plink files including imputed ancient genomes with coverage >0.2× and modern UK Biobank data were run with the Louvain algorithm (*82*) implemented in the R library “igraph” (*83*) with additional significance tests as described in (*26*) using scripts available at https://github.com/SABiagini/Louvain (accessed in October 2021).

### Genetic kinship analysis

Pseudohaploid genotypes of 171 (coverage >0.01×, not including two individuals from the plague pit at Bene’t Street) later medieval genomes at 5,494,912 positions with MAF > 0.05 in the UK10K subset of the HRC panel were called with ANGSD version 0.917. For the comparison with published studies, the merged PLINK file from the PCA analysis was used and select populations retained using plink --keep and converted to .tped. The ANGSD output files were converted to .tped format, which was used as an input for kinship analyses with READ (*3*). Given highly homogenous ancestry in all study sites, individuals from all sites were analyzed together for Fig. 3 as well as separately to test for potential biases in normalized P0 calculation. In addition to first- and second-degree relationships, we also estimated P0 cutoffs [15/16 = 0.9375 as per (*3*)] for the detection of third-degree relatives while acknowledging that because of lack of relevant empirical data the false-positive and false-negative error rates for this class of relationship remain unknown.

### Runs of homozygosity

We used hapROH (*5*) to detect ROH in 109 ancient genomes with coverage >0.1×. Using information from a reference panel, hapROH has been shown to work for genomes with more than 400K of the 1240K-SNP panel covered at an error rate lower than 3% in pseudo-haploid genotypes (*5*). We note that the requirement is broadly in line with the imputation accuracy we get from coverages as low as 0.1×, where ~60% of common variants (MAF ≥ 0.05) in the HRC panel are recovered with an overall accuracy around 97% for diploid genotypes (table S11). Among common variants in the HRC panel, 853,159 overlap with the 1240K-SNP panel.

1000 Genomes Project data were used to construct the reference haplotypes. We kept the standard parameters in hapROH, which had been optimized for 1240K aDNA genotype data

e_model='haploid', post_model='Standard', random_allele=True, roh_in=1, roh_out=20, roh_jump=300, e_rate=0.01, e_rate_ref=0.0, cutoff_post=0.999, max_gap=0, roh_min_l=0.01

We also tested the effect of imputation using down-sampled genomes of PSN31. There is an excess of inferred short (< 4 cM) ROH tracks at 0.05×, but the results are similar at 0.1× and above (table S8).

### Heterozygosity

Genome-wide heterozygosity and heterozygosity in the HLA region (--chr 6 --from-kb 28477 --to-kb 33448) were estimated with the --het function in PLINK 1.9 (*84*) for imputed genomes with >0.05× coverage and for 5,448,740 sites that had MAF > 0.05 in the HRC reference panel. To assess the impact of imputation accuracy on heterozygosity estimation we plotted the heterozygosity estimates of imputed genomes against their coverage (fig. S15) and observed weakly negative but nonsignificant correlation (*r* = −0.05, *P* = 0.58) and the highest variance in the group of genomes imputed from the lowest (0.05× to 0.1×) coverage. We retained for further analyses only genomes with >0.1× coverage.

### Nucleotide diversity

Nucleotide diversity in the HLA region and throughout the autosomes was estimated using vcftools --window-pi (*85*), which by default outputs result in 10-kb windows, in imputed genomes with >0.1× coverage. Sites where the highest genotype probability is less than 0.99 were set to missing. The results are similar whether we filtered the variants by MAF > 0.05 in the HRC reference panel or not (fig. S13).

### Phenotype prediction

Using the imputed genomes generated in this study (143 individuals with a coverage higher than 0.05×) and previously published (71 individuals) (*21*, *22*, *30*), we extracted genotype calls for 39 of the 41 HIrisPlex-S variants and 74 SNPs involved in diet and diseases, coding the allele information as the number of the effective allele (0, 1, 2) using plink (tables S4 to S6). The diet and disease set of 74 SNPs was selected starting from lists of variants previously analyzed in aDNA studies (*86*–*88*), prioritizing those with a role in the response to pathogenic infection. In addition to the 70 variants that we have previously used for phenotype prediction (*89*, *90*), we also analyzed the four SNPs in immune loci recently detected to be highly differentiated before or after the Second Pandemic (*16*) (table S6). A table with the HIrisPlex-S SNP alleles per individual was uploaded on the HIrisPlex-S webtool (https://hirisplex.erasmusmc.nl/) to obtain probabilities values for each eye, hair, and skin color category per individual. This output was then interpreted following the manual to obtain the final pigmentation prediction for each individual (table S5). We then grouped the individuals by time period. The groups were compared performing an ANOVA statistical test, applying a Bonferroni’s correction on each group of variants (carbohydrate metabolism, lipid metabolism, vitamin metabolism, response to pathogens, autoimmune diseases, other diseases, and pigmentation) (table S4) to set the significance threshold. For only the significant SNPs, we also performed a post hoc Tukey test to identify the significantly different group pairs. For our medieval genomes, we also grouped them by relative time to the Second Pandemic (before or after) and performed a statistical *t* test as reported in table S4.

### Enrichment of immune genes

To test the enrichment for higher allele frequency differences at variant positions of immune genes we used Weir and Cockerham (*91*) *F_ST_* as implemented in PLINK (--fst case-control) on lists of variants derived from the Klunk *et al.* study (*16*), as well as an expanded list of 54,931 variants polymorphic in the HRC panel from the 37,574 putatively neutral regions defined by Gronau *et al.* (*38*) that had MAF > 0.1 in 70 imputed genomes from Cambridge dating to before and after the Black Death. As an expanded set of immunity regions, we extracted 19,940 variants with MAF > 0.1 in the Cambridge cohort from 189,173 exonic regions of the 4723 innate immunity genes curated by InnateDB (www.innatedb.com/).

To explore how our sample size (before Black Death: *n* = 44; after Black Death: *n* = 26) might limit our power to detect extreme *F_ST_* values, we postulated a population of 10,000 individuals from before the Black Death and another of the same size from after the Black Death. For each combination of allele frequencies before and after the Black Death (on a grid of 100 × 100 points), assuming Hardy-Weinberg equilibrium, we sampled from these large populations according to our sample sizes and calculated the observed *F_ST_* values. The sampling was repeated 10,000 times. The power of the analysis was calculated as the proportion of observed *F_ST_* values that exceed a predefined threshold, which was taken from the 95th percentile of the distribution of *F_ST_* from a set of neutral variants according to Klunk *et al.* (*16*).

We note that we already deviate from the authors if we choose the threshold using our own dataset, regardless of considerations about sample size. The variants within the same neutral region used by Klunk *et al.* (*16*) produced a much higher 95th percentile threshold, whether we filter out variants with a minor allele frequency lower than 0.1 (0.027; 95% confidence interval: [0.022, 0.031]) or not (0.030; 95% confidence interval: [0.028, 0.038]). The threshold is even higher when we include variants (MAF > 0.1) in the full list of neutral regions described by Gronau *et al.* (*38*) (0.035; 95% confidence interval: [0.035, 0.036]). None of the *F_ST_* values of the four variants reported by Klunk *et al. (*16**) in their London dataset would pass these thresholds. We believe this is related to the ascertainment of previously unidentified variants from low-coverage data in their study as mentioned in our main text.

Nevertheless we went on to adopt their threshold (0.0089) for our power analysis. We ask “what is the probability of obtaining an *F_ST_* value greater than 0.0089 in our samples given each combination of true population allele frequencies before and after the Black Death.” The results are shown in a heatmap (fig. S14). The gray zone along the diagonal line is the region where the true *F_ST_* is below 0.0089. Expectedly, the closer the true allele frequencies, the lower the power of the analysis. The four squares in the plot correspond to the allele frequencies of the four highly differentiated variants estimated in their London cohort according to Klunk *et al.* (*16*), which are replicated in their Danish cohort. If we assume that our Cambridge cohorts derive from the same meta-population as London, and that the allele frequencies estimated from the pre- and postplague London cohorts reported by Klunk *et al. (*16**) accurately reflect the allele frequencies in this meta-population, the power to obtain *F_ST_* scores higher than 0.0089 given our sample size ranges between 0.50 and 0.71 for those variants highlighted by Klunk *et al. (*16**). Under these assumptions, however, the probabilities of obtaining the small *F_ST_* values as we observe in the Cambridge cohort (table S9) range from low (rs2549794: 0.16; rs17473484: 0.19) to very low (rs1052025: 0.013; rs11571319: 0.016), suggesting that our failure to replicate high differentiation at these variants is unlikely due to our sample size.

## Supplementary Materials

### This PDF file includes:

Supplementary Text Figs. S1 to S15 Legends for tables S1 to S11 References

## References

[1] P. Skoglund, I. Mathieson, Ancient genomics of modern humans: The first decade. Annu. Rev. Genomics Hum. Genet. 19, 381–404 (2018). 29709204 10.1146/annurev-genom-083117-021749

[2] L. Orlando, R. Allaby, P. Skoglund, C. Der Sarkissian, P. W. Stockhammer, M. C. Ávila-Arcos, Q. Fu, J. Krause, E. Willerslev, A. C. Stone, C. Warinner, Ancient DNA analysis. Nat. Rev. Methods Primer 1, 14 (2021).

[3] J. M. Kuhn, M. Jakobsson, T. Günther, Estimating genetic kin relationships in prehistoric populations. PLOS ONE 13, e0195491 (2018). 29684051 10.1371/journal.pone.0195491 PMC5912749

[4] L. M. Cassidy, R. Ó. Maoldúin, T. Kador, A. Lynch, C. Jones, P. C. Woodman, E. Murphy, G. Ramsey, M. Dowd, A. Noonan, C. Campbell, E. R. Jones, V. Mattiangeli, D. G. Bradley, A dynastic elite in monumental Neolithic society. Nature 582, 384–388 (2020). 32555485 10.1038/s41586-020-2378-6 PMC7116870

[5] H. Ringbauer, J. Novembre, M. Steinrücken, Parental relatedness through time revealed by runs of homozygosity in ancient DNA. Nat. Commun. 12, 5425 (2021). 34521843 10.1038/s41467-021-25289-w PMC8440622

[6] C. Warinner, A. Herbig, A. Mann, J. A. Fellows Yates, C. L. Weiß, H. A. Burbano, L. Orlando, J. Krause, A Robust Framework for Microbial Archaeology. Annu. Rev. Genomics Hum. Genet. 18, 321–356 (2017). 28460196 10.1146/annurev-genom-091416-035526 PMC5581243

[7] M. A. Spyrou, K. I. Bos, A. Herbig, J. Krause, Ancient pathogen genomics as an emerging tool for infectious disease research. Nat. Rev. Genet. 20, 323–340 (2019). 30953039 10.1038/s41576-019-0119-1 PMC7097038

[8] S. Marciniak, G. H. Perry, Harnessing ancient genomes to study the history of human adaptation. Nat. Rev. Genet. 18, 659–674 (2017). 28890534 10.1038/nrg.2017.65

[9] E. K. Irving-Pease, R. Muktupavela, M. Dannemann, F. Racimo, Quantitative human paleogenetics: What can ancient DNA tell us about complex trait evolution? Front. Genet. 12, 703541 (2021). 34422004 10.3389/fgene.2021.703541 PMC8371751

[10] C. Cessford, C. L. Scheib, M. Guellil, M. Keller, C. Alexander, S. A. Inskip, J. E. Robb, Beyond Plague Pits: Using genetics to identify responses to plague in medieval cambridgeshire. Eur. J. Archaeol. 24, 496–518 (2021).

[11] O. J. Benedictow, *The Complete History of the Black Death* (Boydell & Brewer, 2021).

[12] R. Horrox, Ed., *The Black Death* (Manchester Medieval Sources series, Manchester Univ. Press, Manchester, reprint., 1995).

[13] A. Dumay, O. Gergaud, M. Roy, J.-P. Hugot, Is Crohn’s Disease the price to pay today for having survived the black death? J. Crohns Colitis 13, 1318–1322 (2019). 30893422 10.1093/ecco-jcc/jjz062

[14] A. Immel, F. M. Key, A. Szolek, R. Barquera, M. K. Robinson, G. F. Harrison, W. H. Palmer, M. A. Spyrou, J. Susat, B. Krause-Kyora, K. I. Bos, S. Forrest, D. I. Hernández-Zaragoza, J. Sauter, U. Solloch, A. H. Schmidt, V. J. Schuenemann, E. Reiter, M. S. Kairies, R. Weiß, S. Arnold, J. Wahl, J. A. Hollenbach, O. Kohlbacher, A. Herbig, P. J. Norman, J. Krause, Analysis of genomic DNA from medieval plague victims suggests long-term effect of Yersinia pestis on human immunity genes. Mol. Biol. Evol. 38, 4059–4076 (2021). 34002224 10.1093/molbev/msab147 PMC8476174

[15] S. Gopalakrishnan, S. S. Ebenesersdóttir, I. K. C. Lundstrøm, G. Turner-Walker, K. H. S. Moore, P. Luisi, A. Margaryan, M. D. Martin, M. R. Ellegaard, Ó. Þ. Magnússon, Á. Sigurðsson, S. Snorradóttir, D. N. Magnúsdóttir, J. E. Laffoon, L. van Dorp, X. Liu, I. Moltke, M. C. Ávila-Arcos, J. G. Schraiber, S. Rasmussen, D. Juan, P. Gelabert, T. de Dios, A. K. Fotakis, M. Iraeta-Orbegozo, Å. J. Vågene, S. D. Denham, A. Christophersen, H. K. Stenøien, F. G. Vieira, S. Liu, T. Günther, T. Kivisild, O. G. Moseng, B. Skar, C. Cheung, M. Sandoval-Velasco, N. Wales, H. Schroeder, P. F. Campos, V. B. Guðmundsdóttir, T. Sicheritz-Ponten, B. Petersen, J. Halgunset, E. Gilbert, G. L. Cavalleri, E. Hovig, I. Kockum, T. Olsson, L. Alfredsson, T. F. Hansen, T. Werge, E. Willerslev, F. Balloux, T. Marques-Bonet, C. Lalueza-Fox, R. Nielsen, K. Stefánsson, A. Helgason, M. T. P. Gilbert, The population genomic legacy of the second plague pandemic. Curr. Biol. 32, 4743–4751.e6 (2022). 36182700 10.1016/j.cub.2022.09.023 PMC9671091

[16] J. Klunk, T. P. Vilgalys, C. E. Demeure, X. Cheng, M. Shiratori, J. Madej, R. Beau, D. Elli, M. I. Patino, R. Redfern, S. N. DeWitte, J. A. Gamble, J. L. Boldsen, A. Carmichael, N. Varlik, K. Eaton, J.-C. Grenier, G. B. Golding, A. Devault, J.-M. Rouillard, V. Yotova, R. Sindeaux, C. J. Ye, M. Bikaran, A. Dumaine, J. F. Brinkworth, D. Missiakas, G. A. Rouleau, M. Steinrücken, J. Pizarro-Cerdá, H. N. Poinar, L. B. Barreiro, Evolution of immune genes is associated with the Black Death. Nature 611, 312–319 (2022). 36261521 10.1038/s41586-022-05349-x PMC9580435

[17] S. N. DeWitte, J. W. Wood, Selectivity of Black Death mortality with respect to preexisting health. Proc. Natl. Acad. Sci. U. S. A. 105, 1436–1441 (2008). 18227518 10.1073/pnas.0705460105 PMC2234162

[18] K. Godde, V. Pasillas, A. Sanchez, Survival analysis of the Black Death: Social inequality of women and the perils of life and death in Medieval London. Am. J. Phys. Anthropol. 173, 168–178 (2020). 32472637 10.1002/ajpa.24081

[19] D. Herlihy, S. K. Cohn, *The Black Death and the Transformation of the West* (Harvard Univ. Press, 1997).

[20] B. M. S. Campbell, *The Great Transition: Climate, Disease and Society in the Late-Medieval World* (Cambridge Univ. Press, ed. 1, 2016).

[21] R. Martiniano, A. Caffell, M. Holst, K. Hunter-Mann, J. Montgomery, G. Müldner, R. L. McLaughlin, M. D. Teasdale, W. Van Rheenen, J. H. Veldink, L. H. Van Den Berg, O. Hardiman, M. Carroll, S. Roskams, J. Oxley, C. Morgan, M. G. Thomas, I. Barnes, C. McDonnell, M. J. Collins, D. G. Bradley, Genomic signals of migration and continuity in Britain before the Anglo-Saxons. Nat. Commun. 7, 10326 (2016). 26783717 10.1038/ncomms10326 PMC4735653

[22] S. Schiffels, W. Haak, P. Paajanen, B. Llamas, E. Popescu, L. Loe, R. Clarke, A. Lyons, R. Mortimer, D. Sayer, C. Tyler-Smith, A. Cooper, R. Durbin, Iron Age and Anglo-Saxon genomes from East England reveal British migration history. Nat. Commun. 7, 10408 (2016). 26783965 10.1038/ncomms10408 PMC4735688

[23] A. Rose, *Life in Medieval Cambridge: An Isotopic Analysis of Diet and Mobility* (University of Cambridge, 2020).

[24] J. Evans, C. Chenery, K. Mee, C. Cartwright, K. Lee, B. G. S. Andrew Marchant, B. G. S. Lina Hannaford, Biosphere Isotope Domains GB (V1): Interactive Website (2018).

[25] D. N. Seidman, S. A. Shenoy, M. Kim, R. Babu, I. G. Woods, T. D. Dyer, D. M. Lehman, J. E. Curran, R. Duggirala, J. Blangero, A. L. Williams, Rapid, phase-free detection of long identity-by-descent segments enables effective relationship classification. Am. J. Hum. Genet. 106, 453–466 (2020). 32197076 10.1016/j.ajhg.2020.02.012 PMC7118564

[26] T. Kivisild, L. Saag, R. Hui, S. A. Biagini, V. Pankratov, E. D’Atanasio, L. Pagani, L. Saag, S. Rootsi, R. Mägi, E. Metspalu, H. Valk, M. Malve, K. Irdt, T. Reisberg, A. Solnik, C. L. Scheib, D. N. Seidman, A. L. Williams, K. Tambets, M. Metspalu, Patterns of genetic connectedness between modern and medieval Estonian genomes reveal the origins of a major ancestry component of the Finnish population. Am. J. Hum. Genet. 108, 1792–1806 (2021). 34411538 10.1016/j.ajhg.2021.07.012 PMC8456179

[27] W. M. Ormrod, B. Lambert, J. Mackman, *Immigrant England, 1300-1550* (Manchester Medieval Studies, Manchester Univ. Press, 2018).

[28] W. M. Ormrod, J. Story, E. M. Tyler, Eds., *Migrants in Medieval England, c. 500-c. 1500* (Proceedings of the British Academy, Oxford Univ. Press, ed. 1, 2020).

[29] S. Leslie, B. Winney, G. Hellenthal, D. Davison, A. Boumertit, T. Day, K. Hutnik, E. C. Royrvik, B. Cunliffe, D. J. Lawson, D. Falush, C. Freeman, M. Pirinen, S. Myers, M. Robinson, P. Donnelly, W. Bodmer, The fine-scale genetic structure of the British population. Nature 519, 309–314 (2015). 25788095 10.1038/nature14230 PMC4632200

[30] A. Margaryan, D. J. Lawson, M. Sikora, F. Racimo, S. Rasmussen, I. Moltke, L. M. Cassidy, E. Jørsboe, A. Ingason, M. W. Pedersen, T. Korneliussen, H. Wilhelmson, M. M. Buś, P. de Barros Damgaard, R. Martiniano, G. Renaud, C. Bhérer, J. V. Moreno-Mayar, A. K. Fotakis, M. Allen, R. Allmäe, M. Molak, E. Cappellini, G. Scorrano, H. McColl, A. Buzhilova, A. Fox, A. Albrechtsen, B. Schütz, B. Skar, C. Arcini, C. Falys, C. H. Jonson, D. Błaszczyk, D. Pezhemsky, G. Turner-Walker, H. Gestsdóttir, I. Lundstrøm, I. Gustin, I. Mainland, I. Potekhina, I. M. Muntoni, J. Cheng, J. Stenderup, J. Ma, J. Gibson, J. Peets, J. Gustafsson, K. H. Iversen, L. Simpson, L. Strand, L. Loe, M. Sikora, M. Florek, M. Vretemark, M. Redknap, M. Bajka, T. Pushkina, M. Søvsø, N. Grigoreva, T. Christensen, O. Kastholm, O. Uldum, P. Favia, P. Holck, S. Sten, S. V. Arge, S. Ellingvåg, V. Moiseyev, W. Bogdanowicz, Y. Magnusson, L. Orlando, P. Pentz, M. D. Jessen, A. Pedersen, M. Collard, D. G. Bradley, M. L. Jørkov, J. Arneborg, N. Lynnerup, N. Price, M. T. P. Gilbert, M. E. Allentoft, J. Bill, S. M. Sindbæk, L. Hedeager, K. Kristiansen, R. Nielsen, T. Werge, E. Willerslev, Population genomics of the Viking world. Nature 585, 390–396 (2020). 32939067 10.1038/s41586-020-2688-8

[31] D. A. van Heel, L. Franke, K. A. Hunt, R. Gwilliam, A. Zhernakova, M. Inouye, M. C. Wapenaar, M. C. N. M. Barnardo, G. Bethel, G. K. T. Holmes, C. Feighery, D. Jewell, D. Kelleher, P. Kumar, S. Travis, J. R. Walters, D. S. Sanders, P. Howdle, J. Swift, R. J. Playford, W. M. McLaren, M. L. Mearin, C. J. Mulder, R. McManus, R. McGinnis, L. R. Cardon, P. Deloukas, C. Wijmenga, A genome-wide association study for celiac disease identifies risk variants in the region harboring IL2 and IL21. Nat. Genet. 39, 827–829 (2007). 17558408 10.1038/ng2058 PMC2274985

[32] A. Zhernakova, B. Z. Alizadeh, M. Bevova, M. A. van Leeuwen, M. J. H. Coenen, B. Franke, L. Franke, M. D. Posthumus, D. A. van Heel, G. van der Steege, T. R. D. J. Radstake, P. Barrera, B. O. Roep, B. P. C. Koeleman, C. Wijmenga, Novel association in chromosome 4q27 region with rheumatoid arthritis and confirmation of Type 1 diabetes point to a general risk locus for autoimmune diseases. Am. J. Hum. Genet. 81, 1284–1288 (2007). 17999365 10.1086/522037 PMC2276343

[33] S. Pabst, T. Fränken, J. Schönau, S. Stier, G. Nickenig, R. Meyer, D. Skowasch, C. Grohé, Transforming growth factor-β gene polymorphisms in different phenotypes of sarcoidosis. Eur. Respir. J. 38, 169–175 (2011). 21148227 10.1183/09031936.00120410

[34] L. Chaitanya, K. Breslin, S. Zuñiga, L. Wirken, E. Pośpiech, M. Kukla-Bartoszek, T. Sijen, P. de Knijff, F. Liu, W. Branicki, M. Kayser, S. Walsh, The HIrisPlex-S system for eye, hair and skin colour prediction from DNA: Introduction and forensic developmental validation. Forensic Sci. Int. Genet. 35, 123–135 (2018). 29753263 10.1016/j.fsigen.2018.04.004

[35] M. Keller, M. Guellil, P. Slavin, L. Saag, K. Irdt, H. Niinemäe, A. Solnik, M. Malve, H. Valk, A. Kriiska, C. Cessford, S. A. Inskip, J. E. Robb, C. Cooper, C. von Planta, M. Seifert, T. Reitmaier, W. A. Baetsen, D. Walker, S. Lösch, S. Szidat, M. Metspalu, T. Kivisild, K. Tambets, C. L. Scheib, A refined phylochronology of the second plague pandemic in Western Eurasia. bioRxiv 2023.07.18.549544 [Preprint]. 19 July 2023. 10.1101/2023.07.18.549544.

[36] M. A. Spyrou, M. Keller, R. I. Tukhbatova, C. L. Scheib, E. A. Nelson, A. Andrades Valtueña, G. U. Neumann, D. Walker, A. Alterauge, N. Carty, C. Cessford, H. Fetz, M. Gourvennec, R. Hartle, M. Henderson, K. von Heyking, S. A. Inskip, S. Kacki, F. M. Key, E. L. Knox, C. Later, P. Maheshwari-Aplin, J. Peters, J. E. Robb, J. Schreiber, T. Kivisild, D. Castex, S. Lösch, M. Harbeck, A. Herbig, K. I. Bos, J. Krause, Phylogeography of the second plague pandemic revealed through analysis of historical Yersinia pestis genomes. Nat. Commun. 10, 4470 (2019). 31578321 10.1038/s41467-019-12154-0 PMC6775055

[37] F.-R. Zhang, W. Huang, S.-M. Chen, L.-D. Sun, H. Liu, Y. Li, Y. Cui, X.-X. Yan, H.-T. Yang, R.-D. Yang, T.-S. Chu, C. Zhang, L. Zhang, J.-W. Han, G.-Q. Yu, C. Quan, Y.-X. Yu, Z. Zhang, B.-Q. Shi, L.-H. Zhang, H. Cheng, C.-Y. Wang, Y. Lin, H.-F. Zheng, X.-A. Fu, X.-B. Zuo, Q. Wang, H. Long, Y.-P. Sun, Y.-L. Cheng, H.-Q. Tian, F.-S. Zhou, H.-X. Liu, W.-S. Lu, S.-M. He, W.-L. Du, M. Shen, Q.-Y. Jin, Y. Wang, H.-Q. Low, T. Erwin, N.-H. Yang, J.-Y. Li, X. Zhao, Y.-L. Jiao, L.-G. Mao, G. Yin, Z.-X. Jiang, X.-D. Wang, J.-P. Yu, Z.-H. Hu, C.-H. Gong, Y.-Q. Liu, R.-Y. Liu, D.-M. Wang, D. Wei, J.-X. Liu, W.-K. Cao, H.-Z. Cao, Y.-P. Li, W.-G. Yan, S.-Y. Wei, K.-J. Wang, M. L. Hibberd, S. Yang, X.-J. Zhang, J.-J. Liu, Genomewide association study of leprosy. N. Engl. J. Med. 361, 2609–2618 (2009). 20018961 10.1056/NEJMoa0903753

[38] I. Gronau, M. J. Hubisz, B. Gulko, C. G. Danko, A. Siepel, Bayesian inference of ancient human demography from individual genome sequences. Nat. Genet. 43, 1031–1034 (2011). 21926973 10.1038/ng.937 PMC3245873

[39] S. McCarthy, S. Das, W. Kretzschmar, O. Delaneau, A. R. Wood, A. Teumer, H. M. Kang, C. Fuchsberger, P. Danecek, K. Sharp, Y. Luo, C. Sidore, A. Kwong, N. Timpson, S. Koskinen, S. Vrieze, L. J. Scott, H. Zhang, A. Mahajan, J. Veldink, U. Peters, C. Pato, C. M. Van Duijn, C. E. Gillies, I. Gandin, M. Mezzavilla, A. Gilly, M. Cocca, M. Traglia, A. Angius, J. C. Barrett, D. Boomsma, K. Branham, G. Breen, C. M. Brummett, F. Busonero, H. Campbell, A. Chan, S. Chen, E. Chew, F. S. Collins, L. J. Corbin, G. D. Smith, G. Dedoussis, M. Dorr, A. E. Farmaki, L. Ferrucci, L. Forer, R. M. Fraser, S. Gabriel, S. Levy, L. Groop, T. Harrison, A. Hattersley, O. L. Holmen, K. Hveem, M. Kretzler, J. C. Lee, M. McGue, T. Meitinger, D. Melzer, J. L. Min, K. L. Mohlke, J. B. Vincent, M. Nauck, D. Nickerson, A. Palotie, M. Pato, N. Pirastu, M. McInnis, J. B. Richards, C. Sala, V. Salomaa, D. Schlessinger, S. Schoenherr, P. E. Slagboom, K. Small, T. Spector, D. Stambolian, M. Tuke, J. Tuomilehto, L. H. Van Den Berg, W. Van Rheenen, U. Volker, C. Wijmenga, D. Toniolo, E. Zeggini, P. Gasparini, M. G. Sampson, J. F. Wilson, T. Frayling, P. I. W. De Bakker, M. A. Swertz, S. McCarroll, C. Kooperberg, A. Dekker, D. Altshuler, C. Willer, W. Iacono, S. Ripatti, N. Soranzo, K. Walter, A. Swaroop, F. Cucca, C. A. Anderson, R. M. Myers, M. Boehnke, M. I. McCarthy, R. Durbin, G. Abecasis, J. Marchini, A reference panel of 64,976 haplotypes for genotype imputation. Nat. Genet. 48, 1279–1283 (2016). 27548312 10.1038/ng.3643 PMC5388176

[40] J. Gretzinger, D. Sayer, P. Justeau, E. Altena, M. Pala, K. Dulias, C. J. Edwards, S. Jodoin, L. Lacher, S. Sabin, Å. J. Vågene, W. Haak, S. S. Ebenesersdóttir, K. H. S. Moore, R. Radzeviciute, K. Schmidt, S. Brace, M. A. Bager, N. Patterson, L. Papac, N. Broomandkhoshbacht, K. Callan, É. Harney, L. Iliev, A. M. Lawson, M. Michel, K. Stewardson, F. Zalzala, N. Rohland, S. Kappelhoff-Beckmann, F. Both, D. Winger, D. Neumann, L. Saalow, S. Krabath, S. Beckett, M. Van Twest, N. Faulkner, C. Read, T. Barton, J. Caruth, J. Hines, B. Krause-Kyora, U. Warnke, V. J. Schuenemann, I. Barnes, H. Dahlström, J. J. Clausen, A. Richardson, E. Popescu, N. Dodwell, S. Ladd, T. Phillips, R. Mortimer, F. Sayer, D. Swales, A. Stewart, D. Powlesland, R. Kenyon, L. Ladle, C. Peek, S. Grefen-Peters, P. Ponce, R. Daniels, C. Spall, J. Woolcock, A. M. Jones, A. V. Roberts, R. Symmons, A. C. Rawden, A. Cooper, K. I. Bos, T. Booth, H. Schroeder, M. G. Thomas, A. Helgason, M. B. Richards, D. Reich, J. Krause, S. Schiffels, The Anglo-Saxon migration and the formation of the early English gene pool. Nature 610, 112–119 (2022). 36131019 10.1038/s41586-022-05247-2 PMC9534755

[41] J. Klunk, A. T. Duggan, R. Redfern, J. Gamble, J. L. Boldsen, G. B. Golding, B. S. Walter, K. Eaton, J. Stangroom, J. M. Rouillard, A. Devault, S. N. DeWitte, H. N. Poinar, Genetic resiliency and the Black Death: No apparent loss of mitogenomic diversity due to the Black Death in medieval London and Denmark. Am. J. Phys. Anthropol. 169, 240–252 (2019). 30964548 10.1002/ajpa.23820

[42] M. Lewis, J. Montgomery, Youth mobility, migration, and health before and after the black death. Bioarchaeology Int. 7, 111–129 (2023).

[43] G. Caserta, Considerations about the marriage regulations of Canon Law and the apostolic penitentiary in late Middle Ages. Ius Canonicum XLVII, 119–139 (2007).

[44] J. C. Stephens, D. E. Reich, D. B. Goldstein, H. D. Shin, M. W. Smith, M. Carrington, C. Winkler, G. A. Huttley, R. Allikmets, L. Schriml, B. Gerrard, M. Malasky, M. D. Ramos, S. Morlot, M. Tzetis, C. Oddoux, F. S. di Giovine, G. Nasioulas, D. Chandler, M. Aseev, M. Hanson, L. Kalaydjieva, D. Glavac, P. Gasparini, E. Kanavakis, M. Claustres, M. Kambouris, H. Ostrer, G. Duff, V. Baranov, H. Sibul, A. Metspalu, D. Goldman, N. Martin, D. Duffy, J. Schmidtke, X. Estivill, S. J. O’Brien, M. Dean, Dating the origin of the *CCR5*-Δ32 AIDS-resistance Allele by the coalescence of haplotypes. Am. J. Hum. Genet. 62, 1507–1515 (1998). 9585595 10.1086/301867 PMC1377146

[45] D. Y. C. Brandt, J. César, J. Goudet, D. Meyer, The effect of balancing selection on population differentiation: A study with HLA genes. G3 (Bethesda) 8, 2805–2815 (2018). 29950428 10.1534/g3.118.200367 PMC6071603

[46] Y. Souilmi, R. Tobler, A. Johar, M. Williams, S. T. Grey, J. Schmidt, J. C. Teixeira, A. Rohrlach, J. Tuke, O. Johnson, G. Gower, C. Turney, M. Cox, A. Cooper, C. D. Huber, Admixture has obscured signals of historical hard sweeps in humans. Nat. Ecol. Evol. 6, 2003–2015 (2022). 36316412 10.1038/s41559-022-01914-9 PMC9715430

[47] B. Ferwerda, M. B. B. McCall, M. C. de Vries, J. Hopman, B. Maiga, A. Dolo, O. Doumbo, M. Daou, D. de Jong, L. A. B. Joosten, R. A. Tissingh, F. A. G. Reubsaet, R. Sauerwein, J. W. M. van der Meer, A. J. A. M. van der Ven, M. G. Netea, Caspase-12 and the inflammatory response to Yersinia pestis. PLOS ONE 4, e6870 (2009). 19721713 10.1371/journal.pone.0006870 PMC2730527

[48] R. M. Clay, *The Mediaeval Hospitals of England* (Methuen, 1909).

[49] P. Richards, *The Medieval Leper and His Northern Heirs* (D.S. Brewer Ltd, 1977).

[50] A. R. Barton, C. G. Santander, P. Skoglund, I. Moltke, D. Reich, I. Mathieson, Insufficient evidence for natural selection associated with the Black Death, Genomics, 2023); 10.1101/2023.03.14.532615. PMC11938207 39972236

[51] P. G. Bronson, S. J. Mack, H. A. Erlich, M. Slatkin, A sequence-based approach demonstrates that balancing selection in classical human leukocyte antigen (HLA) loci is asymmetric. Hum. Mol. Genet. 22, 252–261 (2013). 23065702 10.1093/hmg/dds424 PMC3526157

[52] A. S. Maróstica, K. Nunes, E. C. Castelli, N. S. B. Silva, B. S. Weir, J. Goudet, D. Meyer, How HLA diversity is apportioned: influence of selection and relevance to transplantation. Philos. Trans. R. Soc. B Biol. Sci. 377, 20200420 (2022). 10.1098/rstb.2020.0420 PMC9014195 35430892

[53] D. Enard, D. A. Petrov, Evidence that RNA viruses drove adaptive introgression between Neanderthals and modern humans. Cell 175, 360–371.e13 (2018). 30290142 10.1016/j.cell.2018.08.034 PMC6176737

[54] E. Dunbar, G. T. Cook, P. Naysmith, B. G. Tripney, S. Xu, AMS ^14^C dating at the Scottish Universities environmental research centre (SUERC) radiocarbon dating laboratory. Radiocarbon 58, 9–23 (2016).

[55] C. Bronk Ramsey, Bayesian analysis of radiocarbon dates. Radiocarbon 51, 337–360 (2009).

[56] C. Bronk Ramsey, S. Lee, Recent and planned developments of the program OxCal. Radiocarbon 55, 720–730 (2013).

[57] P. J. Reimer, E. Bard, A. Bayliss, J. W. Beck, P. G. Blackwell, C. B. Ramsey, C. E. Buck, H. Cheng, R. L. Edwards, M. Friedrich, P. M. Grootes, T. P. Guilderson, H. Haflidason, I. Hajdas, C. Hatté, T. J. Heaton, D. L. Hoffmann, A. G. Hogg, K. A. Hughen, K. F. Kaiser, B. Kromer, S. W. Manning, M. Niu, R. W. Reimer, D. A. Richards, E. M. Scott, J. R. Southon, R. A. Staff, C. S. M. Turney, J. van der Plicht, IntCal13 and marine13 radiocarbon age calibration curves 0–50,000 years cal BP. Radiocarbon 55, 1869–1887 (2013).

[58] P. J. Reimer, W. E. N. Austin, E. Bard, A. Bayliss, P. G. Blackwell, C. Bronk Ramsey, M. Butzin, H. Cheng, R. L. Edwards, M. Friedrich, P. M. Grootes, T. P. Guilderson, I. Hajdas, T. J. Heaton, A. G. Hogg, K. A. Hughen, B. Kromer, S. W. Manning, R. Muscheler, J. G. Palmer, C. Pearson, J. van der Plicht, R. W. Reimer, D. A. Richards, E. M. Scott, J. R. Southon, C. S. M. Turney, L. Wacker, F. Adolphi, U. Büntgen, M. Capano, S. M. Fahrni, A. Fogtmann-Schulz, R. Friedrich, P. Köhler, S. Kudsk, F. Miyake, J. Olsen, F. Reinig, M. Sakamoto, A. Sookdeo, S. Talamo, The IntCal20 Northern hemisphere radiocarbon age calibration Curve (0–55 cal kBP). Radiocarbon 62, 725–757 (2020).

[59] M. Meyer, M. Kircher, Illumina sequencing library preparation for highly multiplexed target capture and sequencing. Cold Spring Harb. Protoc. 2010, pdb.prot5448 (2010). 20516186 10.1101/pdb.prot5448

[60] M. Martin, Cutadapt removes adapter sequences from high-throughput sequencing reads. EMBnet.journal 17, 10 (2011).

[61] H. Li, R. Durbin, Fast and accurate short read alignment with Burrows–Wheeler transform. Bioinformatics 25, 1754–1760 (2009). 19451168 10.1093/bioinformatics/btp324 PMC2705234

[62] H. Li, B. Handsaker, A. Wysoker, T. Fennell, J. Ruan, N. Homer, G. Marth, G. Abecasis, R. Durbin; 1000 Genome Project Data Processing Subgroup, The sequence alignment/map format and SAMtools. Bioinformatics 25, 2078–2079 (2009). 19505943 10.1093/bioinformatics/btp352 PMC2723002

[63] K. Okonechnikov, A. Conesa, F. García-Alcalde, Qualimap 2: Advanced multi-sample quality control for high-throughput sequencing data. Bioinformatics 32, 292–294 (2016). 26428292 10.1093/bioinformatics/btv566 PMC4708105

[64] T. Derrien, J. Estellé, S. Marco Sola, D. G. Knowles, E. Raineri, R. Guigó, P. Ribeca, Fast computation and applications of genome mappability. PLOS ONE 7, e30377 (2012). 22276185 10.1371/journal.pone.0030377 PMC3261895

[65] H. Jónsson, A. Ginolhac, M. Schubert, P. L. F. Johnson, L. Orlando, mapDamage2.0: Fast approximate Bayesian estimates of ancient DNA damage parameters. Bioinforma. Oxf. Engl. 29, 1682–1684 (2013). 10.1093/bioinformatics/btt193 PMC3694634 23613487

[66] Q. Fu, A. Mittnik, P. L. F. Johnson, K. Bos, M. Lari, R. Bollongino, C. Sun, L. Giemsch, R. Schmitz, J. Burger, A. M. Ronchitelli, F. Martini, R. G. Cremonesi, J. Svoboda, P. Bauer, D. Caramelli, S. Castellano, D. Reich, S. Pääbo, J. Krause, A revised timescale for human evolution based on ancient mitochondrial genomes. Curr. Biol. 23, 553–559 (2013). 23523248 10.1016/j.cub.2013.02.044 PMC5036973

[67] T. S. Korneliussen, A. Albrechtsen, R. Nielsen, ANGSD: Analysis of next generation sequencing data. BMC Bioinformatics 15, 356 (2014). 25420514 10.1186/s12859-014-0356-4 PMC4248462

[68] P. Skoglund, J. Storå, A. Götherström, M. Jakobsson, Accurate sex identification of ancient human remains using DNA shotgun sequencing. J. Archaeol. Sci. 40, 4477–4482 (2013).

[69] H. Weissensteiner, D. Pacher, A. Kloss-Brandstätter, L. Forer, G. Specht, H.-J. Bandelt, F. Kronenberg, A. Salas, S. Schönherr, HaploGrep 2: Mitochondrial haplogroup classification in the era of high-throughput sequencing. Nucleic Acids Res. 44, W58–W63 (2016). 27084951 10.1093/nar/gkw233 PMC4987869

[70] P. Hallast, C. Batini, D. Zadik, P. Maisano Delser, J. H. Wetton, E. Arroyo-Pardo, G. L. Cavalleri, P. de Knijff, G. Destro Bisol, B. M. Dupuy, H. A. Eriksen, L. B. Jorde, T. E. King, M. H. Larmuseau, A. Lopez de Munain, A. M. Lopez-Parra, A. Loutradis, J. Milasin, A. Novelletto, H. Pamjav, A. Sajantila, W. Schempp, M. Sears, A. Tolun, C. Tyler-Smith, A. Van Geystelen, S. Watkins, B. Winney, M. A. Jobling, The Y-chromosome tree bursts into leaf: 13,000 high-confidence SNPs covering the majority of known clades. Mol. Biol. Evol. 32, 661–673 (2015). 25468874 10.1093/molbev/msu327 PMC4327154

[71] M. Karmin, L. Saag, M. Vicente, M. A. Wilson Sayres, M. Järve, U. G. Talas, S. Rootsi, A. M. Ilumäe, R. Mägi, M. Mitt, L. Pagani, T. Puurand, Z. Faltyskova, F. Clemente, A. Cardona, E. Metspalu, H. Sahakyan, B. Yunusbayev, G. Hudjashov, M. DeGiorgio, E. L. Loogväli, C. Eichstaedt, M. Eelmets, G. Chaubey, K. Tambets, S. Litvinov, M. Mormina, Y. Xue, Q. Ayub, G. Zoraqi, T. S. Korneliussen, F. Akhatova, J. Lachance, S. Tishkoff, K. Momynaliev, F. X. Ricaut, P. Kusuma, H. Razafindrazaka, D. Pierron, M. P. Cox, G. N. N. Sultana, R. Willerslev, C. Muller, M. Westaway, D. Lambert, V. Skaro, L. Kovačević, S. Turdikulova, D. Dalimova, R. Khusainova, N. Trofimova, V. Akhmetova, I. Khidiyatova, D. V. Lichman, J. Isakova, E. Pocheshkhova, Z. Sabitov, N. A. Barashkov, P. Nymadawa, E. Mihailov, J. W. T. Seng, I. Evseeva, A. B. Migliano, S. Abdullah, G. Andriadze, D. Primorac, L. Atramentova, O. Utevska, L. Yepiskoposyan, D. Marjanović, A. Kushniarevich, D. M. Behar, C. Gilissen, L. Vissers, J. A. Veltman, E. Balanovska, M. Derenko, B. Malyarchuk, A. Metspalu, S. Fedorova, A. Eriksson, A. Manica, F. L. Mendez, T. M. Karafet, K. R. Veeramah, N. Bradman, M. F. Hammer, L. P. Osipova, O. Balanovsky, E. K. Khusnutdinova, K. Johnsen, M. Remm, M., G. Thomas, C. Tyler-Smith, P. A. Underhill, E. Willerslev, R. Nielsen, M. Metspalu, R. Villems, T. Kivisild, A recent bottleneck of Y chromosome diversity coincides with a global change in culture. Genome Res. 25, 459–466 (2015). 25770088 10.1101/gr.186684.114 PMC4381518

[72] G. D. Poznik, Y. Xue, F. L. Mendez, T. F. Willems, A. Massaia, M. A. Wilson Sayres, Q. Ayub, S. A. McCarthy, A. Narechania, S. Kashin, Y. Chen, R. Banerjee, J. L. Rodriguez-Flores, M. Cerezo, H. Shao, M. Gymrek, A. Malhotra, S. Louzada, R. Desalle, G. R. S. Ritchie, E. Cerveira, T. W. Fitzgerald, E. Garrison, A. Marcketta, D. Mittelman, M. Romanovitch, C. Zhang, X. Zheng-Bradley, G. R. Abecasis, S. A. McCarroll, P. Flicek, P. A. Underhill, L. Coin, D. R. Zerbino, F. Yang, C. Lee, L. Clarke, A. Auton, Y. Erlich, R. E. Handsaker, C. D. Bustamante, C. Tyler-Smith, Punctuated bursts in human male demography inferred from 1,244 worldwide Y-chromosome sequences. Nat. Genet. 48, 593–599 (2016). 27111036 10.1038/ng.3559 PMC4884158

[73] R. Martiniano, B. De Sanctis, P. Hallast, R. Durbin, Placing ancient DNA sequences into reference phylogenies. Mol. Biol. Evol. 39, msac017 (2022). 35084493 10.1093/molbev/msac017 PMC8857924

[74] R. Hui, E. D’Atanasio, L. M. Cassidy, C. L. Scheib, T. Kivisild, Evaluating genotype imputation pipeline for ultra-low coverage ancient genomes. Sci. Rep. 10, 18542 (2020). 33122697 10.1038/s41598-020-75387-w PMC7596702

[75] B. L. Browning, S. R. Browning, Genotype imputation with millions of reference samples. Am. J. Hum. Genet. 98, 116–126 (2016). 26748515 10.1016/j.ajhg.2015.11.020 PMC4716681

[76] B. L. Browning, Y. Zhou, S. R. Browning, A one-penny imputed genome from next-generation reference panels. Am. J. Hum. Genet. 103, 338–348 (2018). 30100085 10.1016/j.ajhg.2018.07.015 PMC6128308

[77] 1000 Genomes Project Consortium, A. Auton, L. D. Brooks, R. M. Durbin, E. P. Garrison, H. M. Kang, J. O. Korbel, J. L. Marchini, S. M. Carthy, G. A. M. Vean, G. R. Abecasis, A global reference for human genetic variation. Nature 526, 68–74 (2015). 26432245 10.1038/nature15393 PMC4750478

[78] M. Guellil, M. Keller, J. M. Dittmar, S. A. Inskip, C. Cessford, A. Solnik, T. Kivisild, M. Metspalu, J. E. Robb, C. L. Scheib, An invasive Haemophilus influenzae serotype b infection in an Anglo-Saxon plague victim. Genome Biol. 23, 22 (2022). 35109894 10.1186/s13059-021-02580-z PMC8812261

[79] A. McKenna, M. Hanna, E. Banks, A. Sivachenko, K. Cibulskis, A. Kernytsky, K. Garimella, D. Altshuler, S. Gabriel, M. Daly, M. A. DePristo, The genome analysis toolkit: A MapReduce framework for analyzing next-generation DNA sequencing data. Genome Res. 20, 1297–1303 (2010). 20644199 10.1101/gr.107524.110 PMC2928508

[80] G. Abraham, Y. Qiu, M. Inouye, FlashPCA2: principal component analysis of Biobank-scale genotype datasets. Bioinformatics 33, 2776–2778 (2017). 28475694 10.1093/bioinformatics/btx299

[81] Free Software Foundation, GNU Datamash, (2014); www.gnu.org/software/datamash/.

[82] V. D. Blondel, J.-L. Guillaume, R. Lambiotte, E. Lefebvre, Fast unfolding of communities in large networks. J. Stat. Mech. Theory Exp. 2008, P10008 (2008).

[83] G. Csardi, T. Nepusz, The igraph software package for complex network research. *InterJournal Complex Syst.* 1695, 1–9 (2006).

[84] C. C. Chang, C. C. Chow, L. C. Tellier, S. Vattikuti, S. M. Purcell, J. J. Lee, Second-generation PLINK: Rising to the challenge of larger and richer datasets. GigaScience 4, 7 (2015). 25722852 10.1186/s13742-015-0047-8 PMC4342193

[85] P. Danecek, A. Auton, G. Abecasis, C. A. Albers, E. Banks, M. A. DePristo, R. E. Handsaker, G. Lunter, G. T. Marth, S. T. Sherry, G. McVean, R. Durbin; 1000 Genomes Project Analysis Group, The variant call format and VCFtools. Bioinformatics 27, 2156–2158 (2011). 21653522 10.1093/bioinformatics/btr330 PMC3137218

[86] M. E. Allentoft, M. Sikora, K. G. Sjögren, S. Rasmussen, M. Rasmussen, J. Stenderup, P. B. Damgaard, H. Schroeder, T. Ahlström, L. Vinner, A. S. Malaspinas, A. Margaryan, T. Higham, D. Chivall, N. Lynnerup, L. Harvig, J. Baron, P. D. Casa, P. Dąbrowski, P. R. Duffy, A. V. Ebel, A. Epimakhov, K. Frei, M. Furmanek, T. Gralak, A. Gromov, S. Gronkiewicz, G. Grupe, T. Hajdu, R. Jarysz, V. Khartanovich, A. Khokhlov, V. Kiss, J. Kolář, A. Kriiska, I. Lasak, C. Longhi, G. McGlynn, A. Merkevicius, I. Merkyte, M. Metspalu, R. Mkrtchyan, V. Moiseyev, L. Paja, G. Pálfi, D. Pokutta, Ł. Pospieszny, T. Douglas Price, L. Saag, M. Sablin, N. Shishlina, V. Smrčka, V. I. Soenov, V. Szeverényi, G. Tóth, S. V. Trifanova, L. Varul, M. Vicze, L. Yepiskoposyan, V. Zhitenev, L. Orlando, T. Sicheritz-Pontén, S. Brunak, R. Nielsen, K. Kristiansen, E. Willerslev, Population genomics of Bronze Age Eurasia. Nature 522, 167–172 (2015). 26062507 10.1038/nature14507

[87] T. Günther, H. Malmström, E. M. Svensson, A. Omrak, F. Sánchez-Quinto, G. M. Kılınç, M. Krzewińska, G. Eriksson, M. Fraser, H. Edlund, A. R. Munters, A. Coutinho, L. G. Simões, M. Vicente, A. Sjölander, B. Jansen Sellevold, R. Jørgensen, P. Claes, M. D. Shriver, C. Valdiosera, M. G. Netea, J. Apel, K. Lidén, B. Skar, J. Storå, A. Götherström, M. Jakobsson, Population genomics of Mesolithic Scandinavia: Investigating early postglacial migration routes and high-latitude adaptation. PLoS Biol. 16, e2003703 (2018). 29315301 10.1371/journal.pbio.2003703 PMC5760011

[88] I. Olalde, M. E. Allentoft, F. Sánchez-Quinto, G. Santpere, C. W. K. Chiang, M. DeGiorgio, J. Prado-Martinez, J. A. Rodríguez, S. Rasmussen, J. Quilez, O. Ramírez, U. M. Marigorta, M. Fernández-Callejo, M. E. Prada, J. M. V. Encinas, R. Nielsen, M. G. Netea, J. Novembre, R. A. Sturm, P. Sabeti, T. Marquès-Bonet, A. Navarro, E. Willerslev, C. Lalueza-Fox, Derived immune and ancestral pigmentation alleles in a 7,000-year-old Mesolithic European. Nature 507, 225–228 (2014). 24463515 10.1038/nature12960 PMC4269527

[89] L. Saag, S. V. Vasilyev, L. Varul, N. V. Kosorukova, D. V. Gerasimov, S. V. Oshibkina, S. J. Griffith, A. Solnik, L. Saag, E. D’Atanasio, E. Metspalu, M. Reidla, S. Rootsi, T. Kivisild, C. L. Scheib, K. Tambets, A. Kriiska, M. Metspalu, Genetic ancestry changes in stone to bronze age transition in the East European plain. Sci. Adv. 7, eabd6535 (2021). 33523926 10.1126/sciadv.abd6535 PMC7817100

[90] T. Saupe, F. Montinaro, C. Scaggion, N. Carrara, T. Kivisild, E. D’Atanasio, R. Hui, A. Solnik, O. Lebrasseur, G. Larson, L. Alessandri, I. Arienzo, F. De Angelis, M. F. Rolfo, R. Skeates, L. Silvestri, J. Beckett, S. Talamo, A. Dolfini, M. Miari, M. Metspalu, S. Benazzi, C. Capelli, L. Pagani, C. L. Scheib, Ancient genomes reveal structural shifts after the arrival of Steppe-related ancestry in the Italian Peninsula. Curr. Biol. 31, 2576–2591.e12 (2021). 33974848 10.1016/j.cub.2021.04.022

[91] B. S. Weir, C. C. Cockerham, Estimating F-statistics for the analysis of population structure. Evolution 38, 1358–1370 (1984). 28563791 10.1111/j.1558-5646.1984.tb05657.x

[92] C. Cessford, A. Dickens, The Manor of Hintona: The origins and development of Church End, Cherry Hinton. Proc. Camb. Antiqu. Soc. 94, 51–72 (2005).

[93] C. Cessford, A. Slater, Beyond the manor of hintona further thoughts on the development of church end, cherry hinton: The neath farm site. Proc. Camb. Antiqu. Soc. 103, 39–60 (2014).

[94] M. Lally, 69 to 115 Church End, Cherry Hinton, Cambridgeshire: Post Excavation Assessment and Updated Project Design (Archaeological Solutions Report 3012, 2008).

[95] HMC, Sixth Report of the Royal Commission on Historical Manuscripts. Part I: Report and Appendix (HMSO, 1877).

[96] C. Cessford, A. Dickens, Castle Hill, Cambridge: Excavation of Saxon, medieval and post-medieval deposits, Saxon execution site and a medieval coinhoard. Proc. Camb. Antiqu. Soc. 94, 73–102 (2005).

[97] M. Rubin, *Charity and Community in Medieval Cambridge* (Cambridge Univ. Press, ed. 1, 1987.

[98] M. Underwood, Ed., *The Cartulary of the Hospital of St John the Evangelist*, *Cambridge* (Cambridgeshire Records Society Map Series, Cambridgeshire Records Society, 2008).

[99] C. Cessford, The St. John’s hospital cemetery and environs, Cambridge: Contextualizing the medieval urban dead: Contextualizing the medieval urban dead. Archaeol. J. 172, 52–120 (2015).

[100] C. Cessford, Former Old Examination Hall, North Range Buildings, New Museums Site, Cambridge: an archaeological excavation (Cambridge Archaeology Unit, 2017).

[101] C. Cessford, North Range Buildings, New Museums Site, Cambridge: further archaeological investigations (Cambridge Archaeology Unit, 2020).

[102] C. Cessford, B. Neil, The people of the Cambridge Austin friars. Archaeol. J. 179, 383–444 (2022). 10.1080/00665983.2022.2090675 PMC9580237 36277234

[103] C. Cessford, M. Samuel, V. Herring, N. Holder, P. Mills, The architecture of the Augustinian Friary, Cambridge. Antiqu. J. 103, 162–194 (2023).

[104] C. Cessford, A. Hall, B. Mulder, B. Neil, I. Riddler, J. Wiles, E. Cameron, Q. Mould, Buried with their buckles on: Clothed burial at the Augustinian Friary Cambridge. Mediev. Archaeol. 66, 151–187 (2022). 35722222 10.1080/00766097.2022.2065066 PMC9197221

[105] C. Cessford, D. Fallon, Hostel Yard and Environs, Corpus Christi College, Cambridge: An Archaeological Watching Brief” (2006).

[106] G. Rees, A 19th Century Baptist Cemetery at St Matthew’s Primary School, Norfolk Street, Cambridge (Oxford Archaeology East, 2014).

[107] R. Newman, Holy Trinity Church, Cambridge: Archaeological Excavation and Monitoring, 2016–2017 (Archaeology Data Service, 2018).

[108] J. A. Alexander, “Clopton: the life-cycle of a Cambridgeshire village” in *East Anglian Studies*, L. M. Munby, Ed. (Heffer and Sons, 1968), pp. 48–70.

[109] S. Sims, Hemingfords Flood Alleviation Scheme, St Ives, Cambridgeshire: archaeological watching brief report (Oxford Archaeology, 2007).

[110] C. Fowler, I. Olalde, V. Cummings, I. Armit, L. Büster, S. Cuthbert, N. Rohland, O. Cheronet, R. Pinhasi, D. Reich, A high-resolution picture of kinship practices in an Early Neolithic tomb. Nature 601, 584–587 (2022). 34937939 10.1038/s41586-021-04241-4 PMC8896835

[111] I. Olalde, S. Brace, M. E. Allentoft, I. Armit, K. Kristiansen, T. Booth, N. Rohland, S. Mallick, A. Szécsényi-Nagy, A. Mittnik, E. Altena, M. Lipson, I. Lazaridis, T. K. Harper, N. Patterson, N. Broomandkhoshbacht, Y. Diekmann, Z. Faltyskova, D. Fernandes, M. Ferry, E. Harney, P. De Knijff, M. Michel, J. Oppenheimer, K. Stewardson, A. Barclay, K. W. Alt, C. Liesau, P. Rios, C. Blasco, J. V. Miguel, R. M. Garcia, A. A. Fernandez, E. Banffy, M. Bernabo-Brea, D. Billoin, C. Bonsall, L. Bonsall, T. Allen, L. Buster, S. Carver, L. C. Navarro, O. E. Craig, G. T. Cook, B. Cunliffe, A. Denaire, K. E. Dinwiddy, N. Dodwell, M. Ernee, C. Evans, M. Kucharik, J. F. Farre, C. Fowler, M. Gazenbeek, R. G. Pena, M. Haber-Uriarte, E. Haduch, G. Hey, N. Jowett, T. Knowles, K. Massy, S. Pfrengle, P. Lefranc, O. Lemercier, A. Lefebvre, C. H. Martinez, V. G. Olmo, A. B. Ramirez, J. L. Maurandi, T. Majo, J. I. McKinley, K. McSweeney, B. G. Mende, A. Mod, G. Kulcsar, V. Kiss, A. Czene, R. Patay, A. Endrodi, K. Kohler, T. Hajdu, T. Szeniczey, J. Dani, Z. Bernert, M. Hoole, O. Cheronet, D. Keating, P. Veleminsky, M. Dobe, F. Candilio, F. Brown, R. F. Fernandez, A. M. Herrero-Corral, S. Tusa, E. Carnieri, L. Lentini, A. Valenti, A. Zanini, C. Waddington, G. Delibes, E. Guerra-Doce, B. Neil, M. Brittain, M. Luke, R. Mortimer, J. Desideri, M. Besse, G. Brucken, M. Furmanek, A. Hauszko, M. Mackiewicz, A. Rapinski, S. Leach, I. Soriano, K. T. Lillios, J. L. Cardoso, M. P. Pearson, P. Wodarczak, T. D. Price, P. Prieto, P. J. Rey, R. Risch, M. A. R. Guerra, A. Schmitt, J. Serralongue, A. M. Silva, V. Smrcka, L. Vergnaud, J. Zilhao, D. Caramelli, T. Higham, M. G. Thomas, D. J. Kennett, H. Fokkens, V. Heyd, A. Sheridan, K. G. Sjogren, P. W. Stockhammer, J. Krause, R. Pinhasi, W. Haak, I. Barnes, C. Lalueza-Fox, D. Reich, The Beaker phenomenon and the genomic transformation of northwest Europe. Nature 555, 190–196 (2018). 29466337 10.1038/nature25738 PMC5973796

[112] N. Patterson, M. Isakov, T. Booth, L. Büster, C.-E. Fischer, I. Olalde, H. Ringbauer, A. Akbari, O. Cheronet, M. Bleasdale, N. Adamski, E. Altena, R. Bernardos, S. Brace, N. Broomandkhoshbacht, K. Callan, F. Candilio, B. Culleton, E. Curtis, L. Demetz, K. S. D. Carlson, C. J. Edwards, D. M. Fernandes, M. G. B. Foody, S. Freilich, H. Goodchild, A. Kearns, A. M. Lawson, I. Lazaridis, M. Mah, S. Mallick, K. Mandl, A. Micco, M. Michel, G. B. Morante, J. Oppenheimer, K. T. Özdoğan, L. Qiu, C. Schattke, K. Stewardson, J. N. Workman, F. Zalzala, Z. Zhang, B. Agustí, T. Allen, K. Almássy, L. Amkreutz, A. Ash, C. Baillif-Ducros, A. Barclay, L. Bartosiewicz, K. Baxter, Z. Bernert, J. Blažek, M. Bodružić, P. Boissinot, C. Bonsall, P. Bradley, M. Brittain, A. Brookes, F. Brown, L. Brown, R. Brunning, C. Budd, J. Burmaz, S. Canet, S. Carnicero-Cáceres, M. Čaušević-Bully, A. Chamberlain, S. Chauvin, S. Clough, N. Čondić, A. Coppa, O. Craig, M. Črešnar, V. Cummings, S. Czifra, A. Danielisová, R. Daniels, A. Davies, P. De Jersey, J. Deacon, C. Deminger, P. W. Ditchfield, M. Dizdar, M. Dobeš, M. Dobisíková, L. Domboróczki, G. Drinkall, A. Đukić, M. Ernée, C. Evans, J. Evans, M. Fernández-Götz, S. Filipović, A. Fitzpatrick, H. Fokkens, C. Fowler, A. Fox, Z. Gallina, M. Gamble, M. R. G. Morales, B. González-Rabanal, A. Green, K. Gyenesei, D. Habermehl, T. Hajdu, D. Hamilton, J. Harris, C. Hayden, J. Hendriks, B. Hernu, G. Hey, M. Horňák, G. Ilon, E. Istvánovits, A. M. Jones, M. B. Kavur, K. Kazek, R. A. Kenyon, A. Khreisheh, V. Kiss, J. Kleijne, M. Knight, L. M. Kootker, P. F. Kovács, A. Kozubová, G. Kulcsár, V. Kulcsár, C. Le Pennec, M. Legge, M. Leivers, L. Loe, O. López-Costas, T. Lord, D. Los, J. Lyall, A. B. Marín-Arroyo, P. Mason, D. Matošević, A. Maxted, L. McIntyre, J. McKinley, K. McSweeney, B. Meijlink, B. G. Mende, M. Menđušić, M. Metlička, S. Meyer, K. Mihovilić, L. Milasinovic, S. Minnitt, J. Moore, G. Morley, G. Mullan, M. Musilová, B. Neil, R. Nicholls, M. Novak, M. Pala, M. Papworth, C. Paresys, R. Patten, D. Perkić, K. Pesti, A. Petit, K. Petriščáková, C. Pichon, C. Pickard, Z. Pilling, T. D. Price, S. Radović, R. Redfern, B. Resutík, D. T. Rhodes, M. B. Richards, A. Roberts, J. Roefstra, P. Sankot, A. Šefčáková, A. Sheridan, S. Skae, M. Šmolíková, K. Somogyi, Á. Somogyvári, M. Stephens, G. Szabó, A. Szécsényi-Nagy, T. Szeniczey, J. Tabor, K. Tankó, C. T. Maria, R. Terry, B. Teržan, M. Teschler-Nicola, J. F. Torres-Martínez, J. Trapp, R. Turle, F. Ujvári, M. Van Der Heiden, P. Veleminsky, B. Veselka, Z. Vytlačil, C. Waddington, P. Ware, P. Wilkinson, L. Wilson, R. Wiseman, E. Young, J. Zaninović, A. Žitňan, C. Lalueza-Fox, P. De Knijff, I. Barnes, P. Halkon, M. G. Thomas, D. J. Kennett, B. Cunliffe, M. Lillie, N. Rohland, R. Pinhasi, I. Armit, D. Reich, Large-scale migration into Britain during the middle to late bronze age. Nature 601, 588–594 (2022). 34937049 10.1038/s41586-021-04287-4 PMC8889665

[113] S. Brace, Y. Diekmann, T. J. Booth, L. van Dorp, Z. Faltyskova, N. Rohland, S. Mallick, I. Olalde, M. Ferry, M. Michel, J. Oppenheimer, N. Broomandkhoshbacht, K. Stewardson, R. Martiniano, S. Walsh, M. Kayser, S. Charlton, G. Hellenthal, I. Armit, R. Schulting, O. E. Craig, A. Sheridan, M. Parker Pearson, C. Stringer, D. Reich, M. G. Thomas, I. Barnes, Ancient genomes indicate population replacement in Early Neolithic Britain. Nat. Ecol. Evol. 3, 765–771 (2019). 30988490 10.1038/s41559-019-0871-9 PMC6520225

[114] C. L. Scheib, R. Hui, E. D’Atanasio, A. W. Wohns, S. A. Inskip, A. Rose, C. Cessford, T. C. O’Connell, J. E. Robb, C. Evans, R. Patten, T. Kivisild, East Anglian early Neolithic monument burial linked to contemporary Megaliths. Ann. Hum. Biol. 46, 145–149 (2019). 31184205 10.1080/03014460.2019.1623912 PMC6816495

[115] N. S. Enattah, T. Sahi, E. Savilahti, J. D. Terwilliger, L. Peltonen, I. Järvelä, Identification of a variant associated with adult-type hypolactasia. Nat. Genet. 30, 233–237 (2002). 11788828 10.1038/ng826

[116] A. M. Hancock, D. B. Witonsky, E. Ehler, G. Alkorta-Aranburu, C. Beall, A. Gebremedhin, R. Sukernik, G. Utermann, J. Pritchard, G. Coop, A. Di Rienzo, Colloquium paper: Human adaptations to diet, subsistence, and ecoregion are due to subtle shifts in allele frequency. Proc. Natl. Acad. Sci. U. S. A. 107 Suppl 2, 8924–8930 (2010). 20445095 10.1073/pnas.0914625107 PMC3024024

[117] Y. S. Aulchenko, S. Ripatti, I. Lindqvist, D. Boomsma, I. M. Heid, P. P. Pramstaller, B. W. J. H. Penninx, A. C. J. W. Janssens, J. F. Wilson, T. Spector, N. G. Martin, N. L. Pedersen, K. O. Kyvik, J. Kaprio, A. Hofman, N. B. Freimer, M.-R. Jarvelin, U. Gyllensten, H. Campbell, I. Rudan, A. Johansson, F. Marroni, C. Hayward, V. Vitart, I. Jonasson, C. Pattaro, A. Wright, N. Hastie, I. Pichler, A. A. Hicks, M. Falchi, G. Willemsen, J.-J. Hottenga, E. J. C. de Geus, G. W. Montgomery, J. Whitfield, P. Magnusson, J. Saharinen, M. Perola, K. Silander, A. Isaacs, E. J. G. Sijbrands, A. G. Uitterlinden, J. C. M. Witteman, B. A. Oostra, P. Elliott, A. Ruokonen, C. Sabatti, C. Gieger, T. Meitinger, F. Kronenberg, A. Döring, H.-E. Wichmann, J. H. Smit, M. I. McCarthy, C. M. van Duijn, L. Peltonen, Loci influencing lipid levels and coronary heart disease risk in 16 European population cohorts. Nat. Genet. 41, 47–55 (2009). 19060911 10.1038/ng.269 PMC2687074

[118] F. Racimo, D. Marnetto, E. Huerta-Sánchez, Signatures of Archaic adaptive introgression in present-day human populations. Mol. Biol. Evol. 34, 296–317 (2017). 27756828 10.1093/molbev/msw216 PMC5400396

[119] C. Sabatti, S. K. Service, A.-L. Hartikainen, A. Pouta, S. Ripatti, J. Brodsky, C. G. Jones, N. A. Zaitlen, T. Varilo, M. Kaakinen, U. Sovio, A. Ruokonen, J. Laitinen, E. Jakkula, L. Coin, C. Hoggart, A. Collins, H. Turunen, S. Gabriel, P. Elliot, M. I. McCarthy, M. J. Daly, M.-R. Järvelin, N. B. Freimer, L. Peltonen, Genome-wide association analysis of metabolic traits in a birth cohort from a founder population. Nat. Genet. 41, 35–46 (2009). 19060910 10.1038/ng.271 PMC2687077

[120] S. Kathiresan, O. Melander, C. Guiducci, A. Surti, N. P. Burtt, M. J. Rieder, G. M. Cooper, C. Roos, B. F. Voight, A. S. Havulinna, B. Wahlstrand, T. Hedner, D. Corella, E. S. Tai, J. M. Ordovas, G. Berglund, E. Vartiainen, P. Jousilahti, B. Hedblad, M.-R. Taskinen, C. Newton-Cheh, V. Salomaa, L. Peltonen, L. Groop, D. M. Altshuler, M. Orho-Melander, Six new loci associated with blood low-density lipoprotein cholesterol, high-density lipoprotein cholesterol or triglycerides in humans. Nat. Genet. 40, 189–197 (2008). 18193044 10.1038/ng.75 PMC2682493

[121] M. García-Closas, D. W. Hein, D. Silverman, N. Malats, M. Yeager, K. Jacobs, M. A. Doll, J. D. Figueroa, D. Baris, M. Schwenn, M. Kogevinas, A. Johnson, N. Chatterjee, L. E. Moore, T. Moeller, F. X. Real, S. Chanock, N. Rothman, A single nucleotide polymorphism tags variation in the arylamine N-acetyltransferase 2 phenotype in populations of European background. Pharmacogenet. Genomics 21, 231–236 (2011). 20739907 10.1097/FPC.0b013e32833e1b54 PMC3003749

[122] A. Sabbagh, P. Darlu, B. Crouau-Roy, E. S. Poloni, Arylamine N-acetyltransferase 2 (NAT2) genetic diversity and traditional subsistence: A worldwide population survey. PLOS ONE 6, e18507 (2011). 21494681 10.1371/journal.pone.0018507 PMC3071824

[123] S. Mathieson, I. Mathieson, FADS1 and the timing of human adaptation to agriculture. Mol. Biol. Evol. 35, 2957–2970 (2018). 30272210 10.1093/molbev/msy180 PMC6278866

[124] T. J. Wang, F. Zhang, J. B. Richards, B. Kestenbaum, J. B. van Meurs, D. Berry, D. P. Kiel, E. A. Streeten, C. Ohlsson, D. L. Koller, L. Peltonen, J. D. Cooper, P. F. O’Reilly, D. K. Houston, N. L. Glazer, L. Vandenput, M. Peacock, J. Shi, F. Rivadeneira, M. I. McCarthy, P. Anneli, I. H. de Boer, M. Mangino, B. Kato, D. J. Smyth, S. L. Booth, P. F. Jacques, G. L. Burke, M. Goodarzi, C.-L. Cheung, M. Wolf, K. Rice, D. Goltzman, N. Hidiroglou, M. Ladouceur, N. J. Wareham, L. J. Hocking, D. Hart, N. K. Arden, C. Cooper, S. Malik, W. D. Fraser, A.-L. Hartikainen, G. Zhai, H. M. Macdonald, N. G. Forouhi, R. J. F. Loos, D. M. Reid, A. Hakim, E. Dennison, Y. Liu, C. Power, H. E. Stevens, L. Jaana, R. S. Vasan, N. Soranzo, J. Bojunga, B. M. Psaty, M. Lorentzon, T. Foroud, T. B. Harris, A. Hofman, J.-O. Jansson, J. A. Cauley, A. G. Uitterlinden, Q. Gibson, M.-R. Järvelin, D. Karasik, D. S. Siscovick, M. J. Econs, S. B. Kritchevsky, J. C. Florez, J. A. Todd, J. Dupuis, E. Hyppönen, T. D. Spector, Common genetic determinants of vitamin D insufficiency: a genome-wide association study. Lancet 376, 180–188 (2010). 20541252 10.1016/S0140-6736(10)60588-0 PMC3086761

[125] H. D. Shin, C. Winkler, J. C. Stephens, J. Bream, H. Young, J. J. Goedert, T. R. O’Brien, D. Vlahov, S. Buchbinder, J. Giorgi, C. Rinaldo, S. Donfield, A. Willoughby, S. J. O’Brien, M. W. Smith, Genetic restriction of HIV-1 pathogenesis to AIDS by promoter alleles of IL10. Proc. Natl. Acad. Sci. U. S. A. 97, 14467–14472 (2000). 11121048 10.1073/pnas.97.26.14467 PMC18942

[126] S. Shrestha, H. W. Wiener, B. Aissani, W. Song, A. Shendre, C. M. Wilson, R. A. Kaslow, J. Tang, Interleukin-10 (IL-10) pathway: genetic variants and outcomes of HIV-1 infection in African American adolescents. PLOS ONE 5, e13384 (2010). 20976276 10.1371/journal.pone.0013384 PMC2954785

[127] J. M. Valverde-Villegas, B. P. Dos Santos, R. M. de Medeiros, V. S. Mattevi, R. K. Lazzaretti, E. Sprinz, R. Kuhmmer, J. A. B. Chies, Endosomal toll-like receptor gene polymorphisms and susceptibility to HIV and HCV co-infection - Differential influence in individuals with distinct ethnic background. Hum. Immunol. 78, 221–226 (2017). 28062211 10.1016/j.humimm.2017.01.001

[128] M. Sironi, M. Biasin, R. Cagliani, D. Forni, M. De Luca, I. Saulle, S. Lo Caputo, F. Mazzotta, J. Macías, J. A. Pineda, A. Caruz, M. Clerici, A common polymorphism in TLR3 confers natural resistance to HIV-1 infection. J. Immunol. Baltim. Md 188, 818–823 (2012). 10.4049/jimmunol.1102179 22174453

[129] M. P. Martin, M. M. Lederman, H. B. Hutcheson, J. J. Goedert, G. W. Nelson, Y. van Kooyk, R. Detels, S. Buchbinder, K. Hoots, D. Vlahov, S. J. O’Brien, M. Carrington, Association of DC-SIGN promoter polymorphism with increased risk for parenteral, but not mucosal, acquisition of human immunodeficiency virus type 1 infection. J. Virol. 78, 14053–14056 (2004). 15564514 10.1128/JVI.78.24.14053-14056.2004 PMC533922

[130] A. Al-Qahtani, M. Al-Ahdal, A. Abdo, F. Sanai, M. Al-Anazi, N. Khalaf, N. A. Viswan, H. Al-Ashgar, H. Al-Humaidan, R. Al-Suwayeh, Z. Hussain, S. Alarifi, M. Al-Okail, F. N. Almajhdi, Toll-like receptor 3 polymorphism and its association with hepatitis B virus infection in Saudi Arabian patients. J. Med. Virol. 84, 1353–1359 (2012). 22825813 10.1002/jmv.23271

[131] C. M. Johnson, E. A. Lyle, K. O. Omueti, V. A. Stepensky, O. Yegin, E. Alpsoy, L. Hamann, R. R. Schumann, R. I. Tapping, Cutting Edge: A common polymorphism impairs cell surface trafficking and functional responses of TLR1 but protects against leprosy. J. Immunol. 178, 7520–7524 (2007). 17548585 10.4049/jimmunol.178.12.7520

[132] S. H. Wong, S. Gochhait, D. Malhotra, F. H. Pettersson, Y. Y. Teo, C. C. Khor, A. Rautanen, S. J. Chapman, T. C. Mills, A. Srivastava, A. Rudko, M. B. Freidin, V. P. Puzyrev, S. Ali, S. Aggarwal, R. Chopra, B. S. N. Reddy, V. K. Garg, S. Roy, S. Meisner, S. K. Hazra, B. Saha, S. Floyd, B. J. Keating, C. Kim, B. P. Fairfax, J. C. Knight, P. C. Hill, R. A. Adegbola, H. Hakonarson, P. E. M. Fine, R. M. Pitchappan, R. N. K. Bamezai, A. V. S. Hill, F. O. Vannberg, Leprosy and the adaptation of human toll-like receptor 1. PLOS Pathog. 6, e1000979 (2010). 20617178 10.1371/journal.ppat.1000979 PMC2895660

[133] R. P. Schuring, L. Hamann, W. R. Faber, D. Pahan, J. H. Richardus, R. R. Schumann, L. Oskam, Polymorphism N248S in the human Toll-like receptor 1 gene is related to leprosy and leprosy reactions. J Infect Dis 199, 1816–1819 (2009). 19456232 10.1086/599121

[134] B. R. Sapkota, M. Macdonald, W. R. Berrington, E. A. Misch, C. Ranjit, M. R. Siddiqui, G. Kaplan, T. R. Hawn, Association of TNF, MBL, and VDR polymorphisms with leprosy phenotypes. Hum. Immunol. 71, 992–998 (2010). 20650301 10.1016/j.humimm.2010.07.001 PMC2941523

[135] G. A. V. Silva, R. Ramasawmy, A. L. Boechat, A. C. Morais, B. K. S. Carvalho, K. B. A. Sousa, V. C. Souza, M. G. S. Cunha, R. H. Barletta-Naveca, M. P. Santos, F. G. Naveca, Association of TNF -1031 C/C as a potential protection marker for leprosy development in Amazonas state patients, Brazil. Hum. Immunol. 76, 137–141 (2015). 25636570 10.1016/j.humimm.2015.01.011

[136] B. Krause-Kyora, J. Susat, F. M. Key, D. Kühnert, E. Bosse, A. Immel, C. Rinne, S.-C. Kornell, D. Yepes, S. Franzenburg, H. O. Heyne, T. Meier, S. Lösch, H. Meller, S. Friederich, N. Nicklisch, K. W. Alt, S. Schreiber, A. Tholey, A. Herbig, A. Nebel, J. Krause, Neolithic and medieval virus genomes reveal complex evolution of hepatitis B. eLife 7, e36666 (2018). 29745896 10.7554/eLife.36666 PMC6008052

[137] E. A. Misch, W. R. Berrington, J. C. Vary Jr., T. R. Hawn, Leprosy and the human genome. Microbiol. Mol. Biol. Rev. 74, 589–620 (2010). 21119019 10.1128/MMBR.00025-10 PMC3008172

[138] V. M. Fava, C. Sales-Marques, A. Alcaïs, M. O. Moraes, E. Schurr, Age-dependent association of TNFSF15/TNFSF8 variants and leprosy type 1 reaction. Front. Immunol. 8, 155 (2017). 28261213 10.3389/fimmu.2017.00155 PMC5306391

[139] Y. Sun, A. Irwanto, L. Toyo-Oka, M. Hong, H. Liu, A. K. Andiappan, H. Choi, Y. Hitomi, G. Yu, Y. Yu, F. Bao, C. Wang, X. Fu, Z. Yue, H. Wang, H. Zhang, M. Kawashima, K. Kojima, M. Nagasaki, M. Nakamura, S.-K. Yang, B. D. Ye, Y. Denise, O. Rotzschke, K. Song, K. Tokunaga, F. Zhang, J. Liu, Fine-mapping analysis revealed complex pleiotropic effect and tissue-specific regulatory mechanism of TNFSF15 in primary biliary cholangitis Crohn’s disease and leprosy. Sci. Rep. 6, 31429 (2016). 27507062 10.1038/srep31429 PMC4979016

[140] C. Sales-Marques, H. Salomão, V. M. Fava, L. E. Alvarado-Arnez, E. P. Amaral, C. C. Cardoso, I. M. F. Dias-Batista, W. L. da Silva, P. Medeiros, M. da Cunha Lopes, F. C. F. Virmond, A. G. Lana, M. O. Pacheco, M. T. Moraes, A. C. Mira, P. Latini, NOD2 and CCDC122-LACC1 genes are associated with leprosy susceptibility in Brazilians. Hum. Genet. 133, 1525–1532 (2014). 25367361 10.1007/s00439-014-1502-9

[141] H. Schurz, M. Daya, M. Möller, E. G. Hoal, M. Salie, TLR1, 2, 4, 6 and 9 variants associated with tuberculosis susceptibility: A systematic review and meta-analysis. PLOS ONE 10, e0139711 (2015). 26430737 10.1371/journal.pone.0139711 PMC4592262

[142] M. Dannemann, A. M. Andrés, J. Kelso, Introgression of Neandertal- and Denisovan-like haplotypes contributes to adaptive variation in human toll-like receptors. Am. J. Hum. Genet. 98, 22–33 (2016). 26748514 10.1016/j.ajhg.2015.11.015 PMC4716682

[143] E. Sánchez, J. M. Sabio, J. L. Callejas, E. de Ramón, R. Garcia-Portales, F. J. García-Hernández, J. Jiménez-Alonso, M. F. González-Escribano, J. Martín, B. P. Koeleman, Association study of genetic variants of pro-inflammatory chemokine and cytokine genes in systemic lupus erythematosus. BMC Med. Genet. 7, 48 (2006). 16719905 10.1186/1471-2350-7-48 PMC1488833

[144] J. A. Shah, J. C. Vary, T. T. H. Chau, N. D. Bang, N. T. B. Yen, J. J. Farrar, S. J. Dunstan, T. R. Hawn, Human TOLLIP regulates TLR2 and TLR4 signaling and its polymorphisms are associated with susceptibility to tuberculosis. J. Immunol. 189, 1737–1746 (2012). 22778396 10.4049/jimmunol.1103541 PMC3428135

[145] C. C. Khor, S. J. Chapman, F. O. Vannberg, A. Dunne, C. Murphy, E. Y. Ling, A. J. Frodsham, A. J. Walley, O. Kyrieleis, A. Khan, C. Aucan, S. Segal, C. E. Moore, K. Knox, S. J. Campbell, C. Lienhardt, A. Scott, P. Aaby, O. Y. Sow, R. T. Grignani, J. Sillah, G. Sirugo, N. Peshu, T. N. Williams, K. Maitland, R. J. O. Davies, D. P. Kwiatkowski, N. P. Day, D. Yala, D. W. Crook, K. Marsh, J. A. Berkley, L. A. J. O’Neill, A. V. S. Hill, A Mal functional variant is associated with protection against invasive pneumococcal disease, bacteremia, malaria and tuberculosis. Nat. Genet. 39, 523–528 (2007). 17322885 10.1038/ng1976 PMC2660299

[146] P. O. Flores-Villanueva, J. A. Ruiz-Morales, C.-H. Song, L. M. Flores, E.-K. Jo, M. Montaño, P. F. Barnes, M. Selman, J. Granados, A functional promoter polymorphism in monocyte chemoattractant protein-1 is associated with increased susceptibility to pulmonary tuberculosis. J. Exp. Med. 202, 1649–1658 (2005). 16352737 10.1084/jem.20050126 PMC2212957

[147] G. Kerner, G. Laval, E. Patin, S. Boisson-Dupuis, L. Abel, J.-L. Casanova, L. Quintana-Murci, Human ancient DNA analyses reveal the high burden of tuberculosis in Europeans over the last 2,000 years. Am. J. Hum. Genet. 108, 517–524 (2021). 33667394 10.1016/j.ajhg.2021.02.009 PMC8008489

[148] M. Saleh, J. P. Vaillancourt, R. K. Graham, M. Huyck, S. M. Srinivasula, E. S. Alnemri, M. H. Steinberg, V. Nolan, C. T. Baldwin, R. S. Hotchkiss, T. G. Buchman, B. A. Zehnbauer, M. R. Hayden, L. A. Farrer, S. Roy, D. W. Nicholson, Differential modulation of endotoxin responsiveness by human caspase-12 polymorphisms. Nature 429, 75–79 (2004). 15129283 10.1038/nature02451

[149] K. Fujikura, Multiple loss-of-function variants of taste receptors in modern humans. Sci. Rep. 5, 12349 (2015). 26307445 10.1038/srep12349 PMC4549710

[150] A. M. Sutherland, K. R. Walley, T.-A. Nakada, A. H. P. Sham, M. M. Wurfel, J. A. Russell, A nonsynonymous polymorphism of IRAK4 associated with increased prevalence of gram-positive infection and decreased response to toll-like receptor ligands. J. Innate Immun. 3, 447–458 (2011). 21576904 10.1159/000323880 PMC3186712

[151] K. A. Hunt, A. Zhernakova, G. Turner, G. A. R. Heap, L. Franke, M. Bruinenberg, J. Romanos, L. C. Dinesen, A. W. Ryan, D. Panesar, R. Gwilliam, F. Takeuchi, W. M. McLaren, G. K. T. Holmes, P. D. Howdle, J. R. F. Walters, D. S. Sanders, R. J. Playford, G. Trynka, C. J. J. Mulder, M. L. Mearin, W. H. M. Verbeek, V. Trimble, F. M. Stevens, C. O’Morain, N. P. Kennedy, D. Kelleher, D. J. Pennington, D. P. Strachan, W. L. McArdle, C. A. Mein, M. C. Wapenaar, P. Deloukas, R. McGinnis, R. McManus, C. Wijmenga, D. A. van Heel, Newly identified genetic risk variants for celiac disease related to the immune response. Nat. Genet. 40, 395–402 (2008). 18311140 10.1038/ng.102 PMC2673512

[152] A. Zhernakova, C. C. Elbers, B. Ferwerda, J. Romanos, G. Trynka, P. C. Dubois, C. G. F. de Kovel, L. Franke, M. Oosting, D. Barisani, M. T. Bardella; Finnish Celiac Disease Study Group, L. A. B. Joosten, P. Saavalainen, D. A. van Heel, C. Catassi, M. G. Netea, C. Wijmenga, Evolutionary and functional analysis of celiac risk loci reveals SH2B3 as a protective factor against bacterial infection. Am. J. Hum. Genet. 86, 970–977 (2010). 20560212 10.1016/j.ajhg.2010.05.004 PMC3032060

[153] A. J. Monsuur, P. I. W. de Bakker, A. Zhernakova, D. Pinto, W. Verduijn, J. Romanos, R. Auricchio, A. Lopez, D. A. van Heel, J. B. A. Crusius, C. Wijmenga, Effective detection of human leukocyte antigen risk alleles in celiac disease using tag single nucleotide polymorphisms. PLOS ONE 3, e2270 (2008). 18509540 10.1371/journal.pone.0002270 PMC2386975

[154] E. A. Stahl, S. Raychaudhuri, E. F. Remmers, G. Xie, S. Eyre, B. P. Thomson, Y. Li, F. A. S. Kurreeman, A. Zhernakova, A. Hinks, C. Guiducci, R. Chen, L. Alfredsson, C. I. Amos, K. G. Ardlie, B. I. R. A. C. Consortium, A. Barton, J. Bowes, E. Brouwer, N. P. Burtt, J. J. Catanese, J. Coblyn, M. J. H. Coenen, K. H. Costenbader, L. A. Criswell, J. B. A. Crusius, J. Cui, P. I. W. de Bakker, P. L. De Jager, B. Ding, P. Emery, E. Flynn, P. Harrison, L. J. Hocking, T. W. J. Huizinga, D. L. Kastner, X. Ke, A. T. Lee, X. Liu, P. Martin, A. W. Morgan, L. Padyukov, M. D. Posthumus, T. R. D. J. Radstake, D. M. Reid, M. Seielstad, M. F. Seldin, N. A. Shadick, S. Steer, P. P. Tak, W. Thomson, A. H. M. van der Helm-van Mil, I. E. van der Horst-Bruinsma, C. E. van der Schoot, P. L. C. M. van Riel, M. E. Weinblatt, A. G. Wilson, G. J. Wolbink, B. P. Wordsworth, Y. E. A. R. Consortium, C. Wijmenga, E. W. Karlson, R. E. M. Toes, N. de Vries, A. B. Begovich, J. Worthington, K. A. Siminovitch, P. K. Gregersen, L. Klareskog, R. M. Plenge, Genome-wide association study meta-analysis identifies seven new rheumatoid arthritis risk loci. Nat. Genet. 42, 508–514 (2010). 20453842 10.1038/ng.582 PMC4243840

[155] V. D. Peltekova, R. F. Wintle, L. A. Rubin, C. I. Amos, Q. Huang, X. Gu, B. Newman, M. Van Oene, D. Cescon, G. Greenberg, A. M. Griffiths, P. H. St George-Hyslop, K. A. Siminovitch, Functional variants of OCTN cation transporter genes are associated with Crohn disease. Nat. Genet. 36, 471–475 (2004). 15107849 10.1038/ng1339

[156] P. Gaj, A. Habior, M. Mikula, J. Ostrowski, Lack of evidence for association of primary sclerosing cholangitis and primary biliary cirrhosis with risk alleles for Crohn’s disease in Polish patients. BMC Med. Genet. 9, 81 (2008). 18715515 10.1186/1471-2350-9-81 PMC2535589

[157] S. Nakagome, S. Mano, L. Kozlowski, J. M. Bujnicki, H. Shibata, Y. Fukumaki, J. R. Kidd, K. K. Kidd, S. Kawamura, H. Oota, Crohn’s disease risk alleles on the NOD2 locus have been maintained by natural selection on standing variation. Mol. Biol. Evol. 29, 1569–1585 (2012). 22319155 10.1093/molbev/mss006 PMC3697811

[158] Z. Liu, P. K. Yadav, X. Xu, J. Su, C. Chen, M. Tang, H. Lin, J. Yu, J. Qian, P.-C. Yang, X. Wang, The increased expression of IL-23 in inflammatory bowel disease promotes intraepithelial and lamina propria lymphocyte inflammatory responses and cytotoxicity. J. Leukoc. Biol. 89, 597–606 (2011). 21227898 10.1189/jlb.0810456

[159] V. Gateva, J. K. Sandling, G. Hom, K. E. Taylor, S. A. Chung, X. Sun, W. Ortmann, R. Kosoy, R. C. Ferreira, G. Nordmark, I. Gunnarsson, E. Svenungsson, L. Padyukov, G. Sturfelt, A. Jönsen, A. A. Bengtsson, S. Rantapää-Dahlqvist, E. C. Baechler, E. E. Brown, G. S. Alarcón, J. C. Edberg, R. Ramsey-Goldman, G. McGwin, J. D. Reveille, L. M. Vilá, R. P. Kimberly, S. Manzi, M. A. Petri, A. Lee, P. K. Gregersen, M. F. Seldin, L. Rönnblom, L. A. Criswell, A.-C. Syvänen, T. W. Behrens, R. R. Graham, A large-scale replication study identifies TNIP1, PRDM1, JAZF1, UHRF1BP1 and IL10 as risk loci for systemic lupus erythematosus. Nat. Genet. 41, 1228–1233 (2009). 19838195 10.1038/ng.468 PMC2925843

[160] H. Bouali, P. Nietert, T. M. Nowling, J. Pandey, M. A. Dooley, G. Cooper, J. Harley, D. L. Kamen, J. Oates, G. Gilkeson, Association of the G-463A myeloperoxidase gene polymorphism with renal disease in african americans with systemic lupus erythematosus. J. Rheumatol. 34, 2028–2034 (2007). 17896805 PMC2798120

[161] Y. Li, W. Liao, M. Cargill, M. Chang, N. Matsunami, B.-J. Feng, A. Poon, K. P. Callis-Duffin, J. J. Catanese, A. M. Bowcock, M. F. Leppert, P.-Y. Kwok, G. G. Krueger, A. B. Begovich, Carriers of rare missense variants in IFIH1 are protected from psoriasis. J. Invest. Dermatol. 130, 2768–2772 (2010). 20668468 10.1038/jid.2010.214 PMC3680368

[162] E. Galimova, R. Rätsep, T. Traks, K. Kingo, V. Escott-Price, S. Kõks, Interleukin-10 family cytokines pathway: Genetic variants and psoriasis. Br. J. Dermatol. 176, 1577–1587 (2017). 28150860 10.1111/bjd.15363

[163] H. Tang, Z. Cheng, W. Ma, Y. Liu, Z. Tong, R. Sun, H. Liu, TLR10 and NFKBIA contributed to the risk of hip osteoarthritis: Systematic evaluation based on Han Chinese population. Sci. Rep. 8, 10243 (2018). 29980729 10.1038/s41598-018-28597-2 PMC6035240

[164] M. S. Rajeevan, I. Dimulescu, J. Murray, V. R. Falkenberg, E. R. Unger, Pathway-focused genetic evaluation of immune and inflammation related genes with chronic fatigue syndrome. Hum. Immunol. 76, 553–560 (2015). 26116897 10.1016/j.humimm.2015.06.014

[165] P. R. Burton, D. G. Clayton, L. R. Cardon, N. Craddock, P. Deloukas, A. Duncanson, D. P. Kwiatkowski, M. I. M. Carthy, W. H. Ouwehand, N. J. Samani, J. A. Todd, P. Donnelly, J. C. Barrett, P. R. Burton, D. Davison, P. Donnelly, J. C. Barrett, P. R. Burton, D. Davison, P. Donnelly, D. Easton, D. Evans, H.-T. Leung, J. L. Marchini, A. P. Morris, C. C. A. Spencer, M. D. Tobin, L. R. Cardon, D. G. Clayton, A. P. Attwood, J. P. Boorman, B. Cant, U. Everson, J. M. Hussey, J. D. Jolley, A. S. Knight, K. Koch, E. Meech, S. Nutland, C. V. Prowse, H. E. Stevens, N. C. Taylor, G. R. Walters, N. M. Walker, N. A. Watkins, T. Winzer, J. A. Todd, W. H. Ouwehand, R. W. Jones, W. L. Mc Ardle, S. M. Ring, D. P. Strachan, M. Pembrey, G. Breen, D. S. Clair, S. Caesar, K. Gordon-Smith, L. Jones, C. Fraser, E. K. Green, D. Grozeva, M. L. Hamshere, P. A. Holmans, I. R. Jones, G. Kirov, V. Moskvina, I. Nikolov, M. C. O’Donovan, M. J. Owen, N. Craddock, D. A. Collier, A. Elkin, A. Farmer, R. Williamson, P. M. Guffin, A. H. Young, I. N. Ferrier, S. G. Ball, A. J. Balmforth, J. H. Barrett, D. T. Bishop, M. M. Iles, A. Maqbool, N. Yuldasheva, A. S. Hall, P. S. Braund, P. R. Burton, R. J. Dixon, M. Mangino, S. Stevens, M. D. Tobin, J. R. Thompson, N. J. Samani, F. Bredin, M. Tremelling, M. Parkes, H. Drummond, C. W. Lees, E. R. Nimmo, J. Satsangi, S. A. Fisher, A. Forbes, C. M. Lewis, C. M. Onnie, N. J. Prescott, J. Sanderson, C. G. Mathew, J. Barbour, M. K. Mohiuddin, C. E. Todhunter, J. C. Mansfield, T. Ahmad, F. R. Cummings, D. P. Jewell, J. Webster, M. J. Brown, D. G. Clayton, G. M. Lathrop, J. Connell, A. Dominiczak, N. J. Samani, C. A. Braga Marcano, B. Burke, R. Dobson, J. Gungadoo, K. L. Lee, P. B. Munroe, S. J. Newhouse, A. Onipinla, C. Wallace, M. Xue, M. Caulfield, M. Farrall, A. Barton; The Biologics in RA Genetics and Genomics, I. N. Bruce, H. Donovan, S. Eyre, P. D. Gilbert, S. L. Hider, A. M. Hinks, S. L. John, C. Potter, A. J. Silman, D. P. M. Symmons, W. Thomson, J. Worthington, D. G. Clayton, D. B. Dunger, S. Nutland, H. E. Stevens, N. M. Walker, B. Widmer, J. A. Todd, T. M. Frayling, R. M. Freathy, H. Lango, J. R. B. Perry, B. M. Shields, M. N. Weedon, A. T. Hattersley, G. A. Hitman, M. Walker, K. S. Elliott, C. J. Groves, C. M. Lindgren, N. W. Rayner, N. J. Timpson, E. Zeggini, M. I. Mc Carthy, M. Newport, G. Sirugo, E. Lyons, F. Vannberg, A. V. S. Hill, L. A. Bradbury, C. Farrar, J. J. Pointon, P. Wordsworth, M. A. Brown, J. A. Franklyn, J. M. Heward, M. J. Simmonds, S. C. L. Gough, S. Seal; Breast Cancer Susceptibility Collaboration, M. R. Stratton, N. Rahman, M. Ban, A. Goris, S. J. Sawcer, A. Compston, D. Conway, M. Jallow, M. Newport, G. Sirugo, K. A. Rockett, D. P. Kwiatkowski, S. J. Bumpstead, A. Chaney, K. Downes, M. J. R. Ghori, R. Gwilliam, S. E. Hunt, M. Inouye, A. Keniry, E. King, R. M. Ginnis, S. Potter, R. Ravindrarajah, P. Whittaker, C. Widden, D. Withers, P. Deloukas; (Wellcome Trust Sanger Institute Hinxton), H.-T. Leung, S. Nutland, H. E. Stevens, N. M. Walker, J. A. Todd, D. Easton, D. G. Clayton, P. R. Burton, M. D. Tobin, J. C. Barrett, D. Evans, A. P. Morris, L. R. Cardon, N. J. Cardin, D. Davison, T. Ferreira, J. Pereira-Gale, I. B. Hallgrimsdóttir, B. N. Howie, J. L. Marchini, C. C. A. Spencer, Z. Su, Y. Y. Teo, D. Vukcevic, P. Donnelly, D. Bentley, M. A. Brown, L. R. Cardon, M. Caulfield, D. G. Clayton, A. Compston, N. Craddock, P. Deloukas, P. Donnelly, M. Farrall, S. C. L. Gough, A. S. Hall, A. T. Hattersley, A. V. S. Hill, D. P. Kwiatkowski, C. G. Mathew, M. I. Mc Carthy, W. H. Ouwehand, M. Parkes, M. Pembrey, N. Rahman, N. J. Samani, M. R. Stratton, J. A. Todd, J. Worthington, Genome-wide association study of 14,000 cases of seven common diseases and 3,000 shared controls. Nature 447, 661–678 (2007). 17554300 10.1038/nature05911 PMC2719288

[166] B. E. Hart, R. I. Tapping, Genetic Diversity of Toll-Like Receptors and Immunity to M. leprae Infection. J. Trop. Med. 2012, 415057 (2012). 22529866 10.1155/2012/415057 PMC3317006

[167] S. Walsh, F. Liu, K. N. Ballantyne, M. van Oven, O. Lao, M. Kayser, IrisPlex: A sensitive DNA tool for accurate prediction of blue and brown eye colour in the absence of ancestry information. Forensic Sci. Int. Genet. 5, 170–180 (2011). 20457092 10.1016/j.fsigen.2010.02.004

[168] S. Walsh, F. Liu, A. Wollstein, L. Kovatsi, A. Ralf, A. Kosiniak-Kamysz, W. Branicki, M. Kayser, The HIrisPlex system for simultaneous prediction of hair and eye colour from DNA. Forensic Sci. Int. Genet. 7, 98–115 (2013). 22917817 10.1016/j.fsigen.2012.07.005

[169] S. Walsh, L. Chaitanya, K. Breslin, C. Muralidharan, A. Bronikowska, E. Pospiech, J. Koller, L. Kovatsi, A. Wollstein, W. Branicki, F. Liu, M. Kayser, Global skin colour prediction from DNA. Hum. Genet. 136, 847–863 (2017). 28500464 10.1007/s00439-017-1808-5 PMC5487854

[170] S. Walsh, L. Chaitanya, L. Clarisse, L. Wirken, J. Draus-Barini, L. Kovatsi, H. Maeda, T. Ishikawa, T. Sijen, P. De Knijff, W. Branicki, F. Liu, M. Kayser, Developmental validation of the HIrisPlex system: DNA-based eye and hair colour prediction for forensic and anthropological usage. Forensic Sci. Int. Genet. 9, 150–161 (2014). 24528593 10.1016/j.fsigen.2013.12.006

