# Supplementary material for: Genetic history of Cambridgeshire before and after the Black Death
Source: Sci Adv. 2024 Jan 17;10(3):eadi5903. doi: 10.1126/sciadv.adi5903 (PMC10793959; doi:10.1126/sciadv.adi5903)
Supplement: Supplementary file 1 — Supplementary Text Figs. S1 to S15 Legends for tables S1 to S11 References [file sciadv.adi5903_sm.pdf]

Science Advances
AAAS

Supplementary Materials for

Genetic history of Cambridgeshire before and after the Black Death

Ruoyun Hui *et al.*

Corresponding author: Ruoyun Hui, rhui@turing.ac.uk; Toomas Kivisild, toomas.kivisild@kuleuven.be

Sci. Adv. **10**, eadi5903 (2023)
DOI: 10.1126/sciadv.adi5903

The PDF file includes:

Supplementary Text
Figs. S1 to S15
Legends for tables S1 to S11
References

Other Supplementary Material for this manuscript includes the following:

Tables S1 to S11

Supplementary Text

Provenance

Here we briefly describe the archaeological sites included in this study and the provenance of the materials studied. More detailed information of all the sites examined by the Wellcome Trust funded ‘After the plague’ project is provided in (*10*), on our project website https://www.aftertheplague.org/, and through individual studies on the sites referred to in the following brief descriptions.

Cherry Hinton

The settlement of Church End Cherry Hinton (Cherry Hinton) is located around six kilometers southeast of Cambridge. In the late 9th to the mid-10th century, a large *thengly* (aristocratic) or proto-manorial center was established (*92*, *93*). The associated timber chapel and graveyard were excavated in 1999 by the Hertfordshire Archaeological Trust (subsequently Archaeological Solutions and now Wardell Armstrong) in advance of development of the site in accordance with the appropriate planning regulations (*94*). The burials have been dated by a combination of stratigraphy, typological dating of associated artefacts particularly pottery, art-historical dating of associated sculpture, parallels for the associated church and radiocarbon dating of a sample of the skeletons. The skeletons were initially studied by Lavinia Ferrante di Ruffano and Tony Waldron for the Hertfordshire Archaeological Trust and were reexamined for the After the Plague project by Sarah Inskip. The human skeletal remains are held by the Historic Environment Team of Cambridgeshire County Council (archaeology@cambridgeshire.gov.uk), to whom any requests for access should be made.

Only part of the cemetery was investigated, including over 670 graves and the remains of *c*. 980 individuals. Most burials were west-east aligned extended supine inhumations, with the head to the west. The cemetery population was estimated to be *c*. 1000–2000. The cemetery probably served an entire thriving Late Saxon to Norman rural settlement, broadly representative of a mixed rural peasant tenant hierarchy engaged primarily but not exclusively in agricultural labor. The burials follow a relatively uniform rite, so there are few archaeological indications of the status of particular individuals. Based on radiocarbon dating plus a range of other evidence the cemetery dates to *c*. 940/990–1120/70. As the population from which those buried at Church End were drawn probably increased over time it is likely that the majority, possibly around two thirds, died after the Norman Conquest. As this settlement was located firmly within the rural hinterland of Cambridge this population provides an important comparator to the town, albeit one that is rather earlier than the main populations that have been studied from there.

In total, 48 individuals from Cherry Hinton were targeted for DNA extraction in this study, including 24 females and 24 males (Table S1). Two of the sampled individuals have been directly radiocarbon dated.

All Saints

All Saints by the Castle (All Saints) is a medieval parish church and associated cemetery in Cambridge. Although not documented until 1217, the church and cemetery are likely to have been founded *c*. 940–1100 and the parish was amalgamated with St Giles in 1365/6 (*95*) (299, fol. 1026).

The cemetery of All Saints was first identified in 1972 in evaluation trenches by John Alexander, then partially investigated by Paul Craddock and Vince Gregory in 1973, with further excavations in 1988 and 1994 (*96*). The work took place in advance of development of the site in accordance with the appropriate planning regulations. The skeletons reported in this study were excavated in 1972 and 1973. The burials have been dated by a combination of documentary sources, stratigraphy, typological dating of associated artefacts particularly pottery, radiocarbon dating of a sample of the skeletons and ancient DNA evidence for *Y. pestis*. The skeletons were initially studied by Charles Bernard Denston of the Duckworth Laboratory and were reexamined for the After the Plague project by Sarah Inskip. The human skeletal remains are held by the Duckworth Laboratory of the Department of Archaeology, University of Cambridge, to whom any requests for access should be made via their website: https://www.arch.cam.ac.uk/institutes-and-facilities-overview/duckworth-laboratory/contact-duckworth

In total just over 210 skeletons have been excavated from the cemetery, out of an estimated original overall total of *c*. 2500–3500. The burials at the cemetery of All Saints probably constitute a broadly representative sample of the parishioners' *c*. 940/1100–1365/6, albeit slightly distorted by several factors. Most burials, probably over 80%, are likely to date to the period after the Norman Conquest. Although the population of All Saints was socially and economically mixed, they are probably somewhat poorer than the average for Cambridge and rather more involved in agricultural activities. The burials follow a relatively uniform rite, so there are few archaeological indications of the status of particular individuals. Radiocarbon dating is compatible with the burials dating to c. 940/1100–1365/6. The burials at All Saints begin and end earlier than the other main Cambridge urban skeletal assemblages, from the Hospital of St John and the Augustinian Friary.

Tooth roots of a total of 49 individuals, including 29 female and 20 male skeletons, were targeted for DNA extraction in this study (Table S1). Four of the sampled individuals have been directly radiocarbon dated. Sequence data of none of the 49 individuals previously screened for plague (*10*) had presented reads mapping uniquely to *Y. pestis* genome.

Hospital of St John

The Hospital of St John the Evangelist in Cambridge (Hospital or Hospital of St John), located near the junction of present-day Bridge and St John's Streets, between the main street of medieval Cambridge and the river Cam, was established by local townspeople *c*. 1190–1200 to care for, principally in a social and spiritual sense rather than medically, the poor, infirm and sick. It did this until it was dissolved to create St John's College in 1511, although it was allegedly already in decline during the 15th century. Considerable textual evidence for the Hospital survives, principally in the College archives (*97*, *98*). The Hospital rapidly came under more formal religious control, and by *c*. 1250 was following the Augustinian rule. Two of the buildings of the medieval Hospital, known as the Infirmary and the Chapel survived with major modifications until the construction of the New Chapel of St John's College in 1863–9.

Excavations were undertaken by the Cambridge Archaeological Unit in advance of development of the site in accordance with the appropriate planning regulations. The burials have been dated by a combination of stratigraphy, documentary sources, typological dating of associated artefacts particularly pottery and radiocarbon dating of a sample of the skeletons. The skeletons were initially studied by Natasha Dodwell of the Cambridge Archaeological Unit and were reexamined for the After the Plague project by Sarah Inskip. The human skeletal remains are held by the Duckworth Laboratory of the Department of Archaeology, University of Cambridge, to whom any requests for access should be made via their website: https://www.arch.cam.ac.uk/institutes-and-facilities-overview/duckworth-laboratory/contact-duckworth

Excavations in 2010–2011, conducted by the Cambridge Archaeological Unit in the detached cemetery of the hospital, located on the opposite side of St John's Street, revealed almost 400 complete or partial skeletons, from a likely original population of *c*. 1000–1500 burials (*99*). The Hospital did not acquire the right to conduct burials until *c*. 1204 and the results of the intensive radiocarbon dating program and other evidence are compatible with burial starting in the early 13th century (*c*. 1204–1214) and indicate that it continued until at least the mid/late 15th century (*c*. 1467–1511).

The burials at the detached cemetery of the Hospital of St John represent an amalgam of various urban groups associated with the institution. Most of the individuals buried there were probably charitable inmates of the Hospital. Various documentary sources describe these inmates as poor, infirm, weak, frail and sick and there are also references to poor scholars. The Hospital specifically excluded pregnant women, people with leprosy, the wounded, 'cripples' and the 'crazy', presumably because these groups required too much care. Also, likely to be represented, but in lower numbers, are corrodians, locals who gave modest gifts in return for burial in the cemetery, and possibly Hospital servants. A range of other individuals such as wealthy benefactors and the religious brethren of the Hospital are unlikely to be present, as they would have been buried elsewhere in or around the Hospital chapel. The burials follow a highly uniform rite, so there are no archaeological indications of the status of particular individuals.

In total, 104 individuals from the Hospital were sampled in this study, including 40 female, 63 male and one skeleton of unknown sex (Table S1). Of the sampled individuals 24 have been directly radiocarbon dated. Assignments to the before and after the Black Death of 1348/9 event could be made, on the basis of the direct radiocarbon dates and stratigraphic relationships, for 71 individuals. Sequence data of none of the 104 individuals previously screened for plague (*10*) had presented reads mapping uniquely to *Y. pestis* genome (Table S1).

Augustinian Friary

The Augustinian Friary (Friary) was established in Cambridge between 1279/80 and 1289, when it was first mentioned in a royal pittance. The Friary grew rapidly and thrived, becoming a *studium generale* or national study house with internal connections in 1318 and having 70 friars present in 1328. It continued until the Dissolution 1538, as one of the most important Augustinian friaries in England and one of the largest institutions in Cambridge.

The skeletons reported in this study were recovered during archaeological excavations in advance of development of the site undertaken by the Cambridge Archaeological Unit in 2016–2017 in accordance with the appropriate planning regulations. The burials have been dated by a combination of stratigraphy, documentary sources, typological dating of associated artefacts particularly pottery, radiocarbon dating of a sample of the skeletons and ancient DNA evidence for *Y. pestis*.. The skeletons were initially studied by Benjamin Neil of the Cambridge Archaeological Unit and were reexamined for the After the Plague project by Sarah Inskip. The human skeletal remains are held by the Historic Environment Team of Cambridgeshire County Council (archaeology@cambridgeshire.gov.uk), to whom any requests for access should be made.

Human remains have been recovered from three locations at the Friary: an early cemetery and the later chapter house and cloister. Twenty-eight burials (26 male and 2 female) included in this study (Table S1) form a subset of 72 burials that were excavated by the Cambridge Archaeological Unit in 2016–2017 and come from the cemetery and chapter house (*100–103*). The friary appears to have acquired the right to burial of individuals in 1290. Based on radiocarbon dating, stratigraphy, artefact typology and architectural criteria the cemetery burials are dated to *c*. 1290–1400/20 and those from the chapter house to *c*. 1330/50–1538. Some of the

burials were accompanied by single buckles located near the pelvis, indicating that the bodies were buried in a clothed state, with surviving evidence for associated leather girdles and some evidence for textiles. Some of the skeletons definitely lacked buckles and these were probably buried in shrouds. It appears that members of the Augustinian order received clothed burial, while shrouded burials are of lay individuals (*104*). The lay individuals would include patrons and benefactors, as well as lay servants of the Friary and corrodians. The 72 burials recovered from the Friary represent only a portion of the estimated 200–700 individuals likely to have been interred there between 1290 and 1538. Three of the 28 individuals examined here have been directly radiocarbon dated and four individuals had tested positive for *Y. pestis* (*10*)

Bene't Street

St Bene't's (a contraction of Benedict's) parochial church in Cambridge was established in *c.* 1000–1050 CE and remains in use, with burial continuing until the 1850s. A strip of land along the western side of the churchyard was transferred to Corpus Christi College between 1352 and 1377, to form an entrance route between Bene't Street and the College. Part of this strip was excavated by the Cambridge Archaeological Unit in 2006, revealing highly truncated individual burials and part of one mass burial (*105*). The skeletons reported in this study were recovered during archaeological excavations undertaken in advance of development of the site by the Cambridge Archaeological Unit in 2005–2006 in accordance with the appropriate planning regulations. The burials have been dated by a combination of stratigraphy, documentary sources, typological dating of associated artefacts particularly pottery and ancient DNA evidence for *Y. pestis*. The skeletons were initially studied by Natasha Dodwell of the Cambridge Archaeological Unit and were reexamined for the After the Plague project by Sarah Inskip. The human skeletal remains are currently held by the Cambridge Archaeological Unit and will be transferred to the Historic Environment Team of Cambridgeshire County Council (archaeology@cambridgeshire.gov.uk), to whom any requests for access should be made.

Among four skeletons from the mass burial whose teeth were tested for DNA, two yielded positive and one tentative identifications of *Y. pestis* (*10*). It is probable that this mass burial relates to the Black Death as it is likely to predate the construction of the College buildings, which took place between 1352 and 1377, and this has recently been confirmed (*35*). These individuals, although none of them have been directly radiocarbon dated, are interpreted as parishioners of St Bene't's, who died during the Black Death in 1349. Three of the tested individuals were male and one was female (Table S1).

Baptist Chapel

The Providence Calvinistic Baptist Chapel (Baptist Chapel) in Cambridge was in use for just four years 1833–1837 CE. Part of the associated cemetery was excavated in 2012, by Oxford Archaeology East in advance of development of the site in accordance with the appropriate planning regulations (*106*). The burials have been dated by a combination of documentary sources, stratigraphy and typological dating of associated artefacts. The skeletons were initially studied by Mark Gibson and Louise Loe of Oxford Archaeology East and were reexamined for the After the Plague project by Sarah Inskip. The human skeletal remains were initially held by Oxford Archaeology East, but will be transferred to the Historic Environment Team of Cambridgeshire County Council (archaeology@cambridgeshire.gov.uk), to whom any requests for access should be made.

There originally may have been a maximum of 20 graves in the investigated area, with at least 16 graves in five rows 11 of which were recorded. The graves were aligned north-northwest to south-southeast and were mainly earth cut, but two were brick-lined and there is evidence for coffins

and shrouds. The majority of the individuals recovered died at a young age and are interpreted as members of non-conformist families in Cambridge, documentary evidence indicates that the individuals who formed the community came from a range of social backgrounds. DNA was extracted from teeth and auditory ossicles of six individuals, including 4 females and 2 males (Table S1).

Holy Trinity

Holy Trinity Church in Cambridge (Holy Trinity) is first mentioned in 1174, when it is said to have been burnt down, and was probably established *c*. 1050–1150 CE. Excavations by the Cambridge Archaeological Unit in 2016–17 in advance of development of the site in accordance with the appropriate planning regulations revealed seventeen articulated burials (*107*). The burials have been dated by a combination of documentary sources, stratigraphy and typological dating of associated artefacts. The skeletons were studied by Benjamin Neil of the Cambridge Archaeological Unit and Sarah Inskip and Jenna Dittmar of the After the Plague project by Sarah Inskip. In accordance with the terms of the diocesan faculty under which the excavations took place and as is standard practice for still active churches the human skeletal remains were reburied at Holy Trinity Church, Cambridge.

Although the human skeletal remains were reburied, they were subject to thorough osteological analysis and a subset of 10 individuals subject to DNA analyses (Table S1). The 10 skeletons (6 males and 4 females) included in this study were placed in coffins in the vaults constructed after a vestry building of 1833/4 and prior to the end of burial in the cemetery in 1855. The individuals are interpreted as parishioners of Holy Trinity, burial in a vault indicates that they were relatively wealthy, and it is likely that the individuals in specific vaults were related to each other, although not necessarily genetically.

Midsummer Common

The circumstances of the discovery of these human skeletal remains is not recorded, but they were probably recovered during documented drainage works in the area. The human skeletal remains have been dated by a combination of documentary sources and radiocarbon dating. The human skeletal remains are held by the Duckworth Laboratory of the Department of Archaeology, University of Cambridge, to whom any requests for access should be made via their website: https://www.arch.cam.ac.uk/institutes-and-facilities-overview/duckworth-laboratory/contact-duckworth.

From 1574 until the last outbreak in Cambridge in 1665/6, some individuals infected with the plague were isolated and moved to pest houses, located some distance from the town in its surrounding fields. Although primarily about quarantine and isolation of the living, parish registers record that from at least 1603 onwards some individuals who died at pest houses were buried there rather than returned to their parish cemetery. The location of the pest houses changed over time, with at least four locations known. One of these sites was Midsummer Common, where pest houses are mentioned in 1593 and 1630. Two skulls discovered in the late 19th or early 20th century at Midsummer Common were presented to the Duckworth Collection by the town clerk, John Edleston Ledsam Whitehead (1853–1923, town clerk 1887–1923). A tooth from one of the skulls was dated to 1450–1630 cal ad (*10*). Both tested individuals were genetically determined to be male and neither of them had tested positive for *Y. pestis*.

Clopton

Clopton was a village in west Cambridgeshire, about 19 kilometers southwest of Cambridge. The village was established by the 10th–11th centuries and appears to have thrived until forcible

enclosure for sheep grazing in *c*. 1480–1520 CE. The church is documented as having gone out of use in 1561. There is good evidence that the village fell primarily within the hinterland of Cambridge, although its location meant that it also had links to urban centers in Hertfordshire. The church of St Mary had been established at Clopton by the late twelfth century, but is probably substantially older. A new church was dedicated in 1352 and this continued to stand after the village was deserted.

John Alexander excavated *c*. 70 skeletons from the church and cemetery between 1960 and 1964, out of over 120 burials with records (*108*). These investigations were undertaken as a research excavation. The burials have been dated by a combination of documentary sources, stratigraphy, typological dating of associated artefacts particularly pottery, radiocarbon dating of a sample of the skeletons and ancient DNA evidence for *Y. pestis*. The skeletons were initially studied by Charles Bernard Denston of the Duckworth Laboratory and were reexamined for the After the Plague project by Sarah Inskip. Some of the human skeletal remains were reburied in the churchyard of Hatley St George, Cambridgeshire, in 1967. The remaining human skeletal remains involved in this study are held by the Duckworth Laboratory of the Department of Archaeology, University of Cambridge, to whom any requests for access should be made via their website: https://www.arch.cam.ac.uk/institutes-and-facilities-overview/duckworth-laboratory/contact-duckworth.

The excavations focused primarily on the cemetery to the south of the church, which would have been where the bulk of the parishioners were buried. The burials follow a highly uniform rite, so there are no archaeological indications of the status of particular individuals. The bulk of the parish population were primarily engaged in agricultural activities, with others engaged in a range of trades and occupations. The limited excavations in and around the church means that the individuals who were buried there, which would have included clerics such as John Thorney as well as wealthy parishioners, are largely absent from the studied human remains. Radiocarbon dating and other evidence is consistent with a date of *c*. 1200–1561 CE for the burials.

DNA was extracted from the tooth roots of 17 individuals, including 13 males and 4 females (Table S1). Three individuals tested positive for *Y. pestis*, two more individuals possibly were positive but not confirmed (Table S1).

Hemingford Grey

Excavations at Meadow Lane, north of Hemingford Grey, 19km northwest of Cambridge were undertaken by Oxford Archaeology in advance of development of the site in accordance with the appropriate planning regulations in 2006, revealing sixteen burials in a late 17th–early 18th-century nonconformist cemetery that has been linked to the Society of Friends or Quakers (*109*). The burials have been dated by a combination of documentary sources, stratigraphy and typological dating of associated artefacts. The skeletons were initially studied by Sharon Clough and Louise Loe of Oxford Archaeology East and were reexamined for the After the Plague project by Sarah Inskip. The human skeletal remains were initially held by Oxford Archaeology East, but will be transferred to the Historic Environment Team of Cambridgeshire County Council (archaeology@cambridgeshire.gov.uk), to whom any requests for access should be made.

Textual evidence for the cemetery comes from the vicar at Hemingford Grey Parish noting in the Parish register burials that took place away from the Parish cemetery at a site referred to as Wobourn or Oubourne. This lists 17 burials between 1681 and 1721, although it is unclear if the list is complete and earlier and later burials are possible. The individuals buried came from Hemingford Grey and the adjacent Parishes of Fenstanton, St Ives and Hemingford Abbot and there are several examples of multiple members of a single family. The individuals are interpreted

as non-conformists, documentary evidence indicates that the individuals who formed this community came from a range of social backgrounds. The burials follow a relatively uniform rite, so there are few archaeological indications of the status of particular individuals.

DNA was extracted from teeth and ribs of 7 individuals, including one female and 6 males (Table S1).

Supplementary Figures

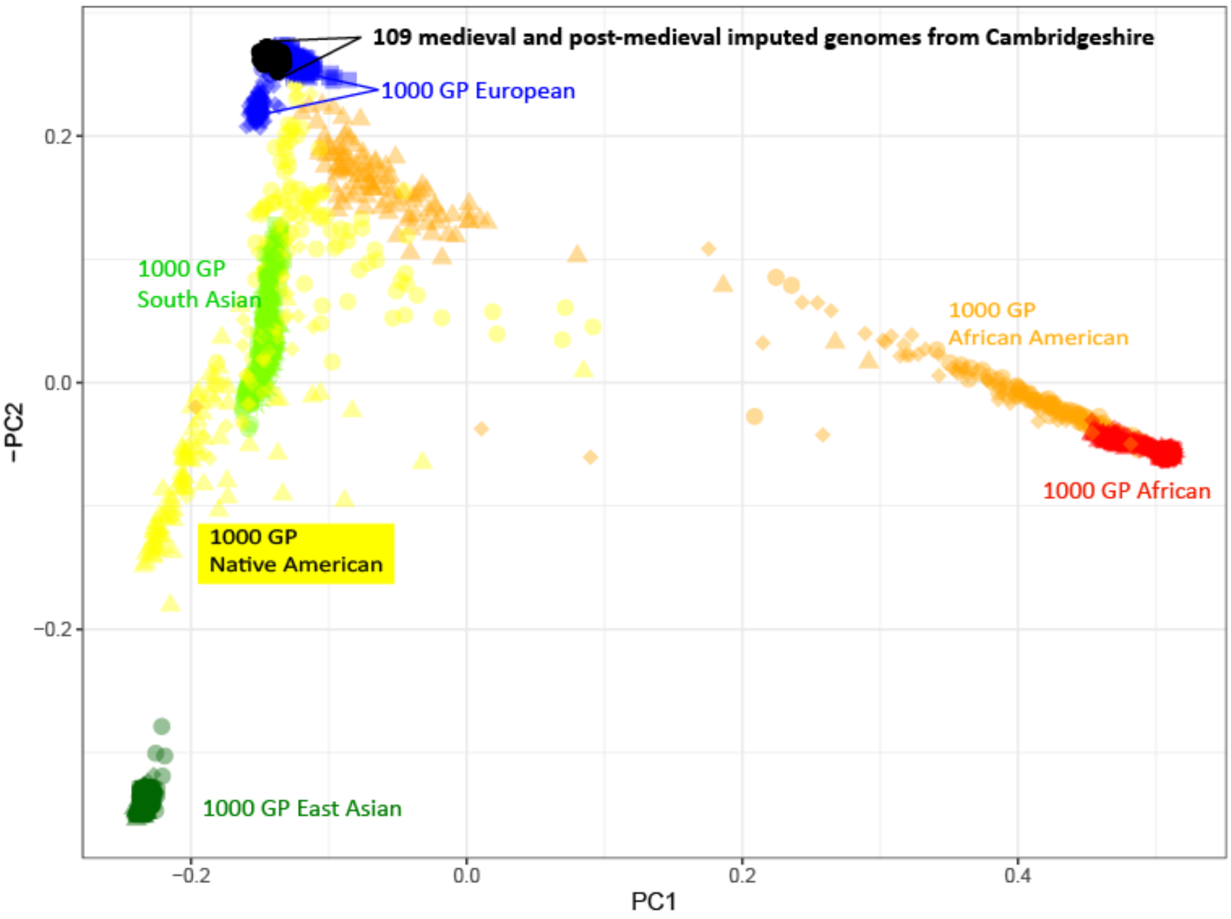

Fig. S1. PCA of 109 later medieval and post-medieval genomes (>0.1x) in context of 1000 Genome Project (1000 GP) data.

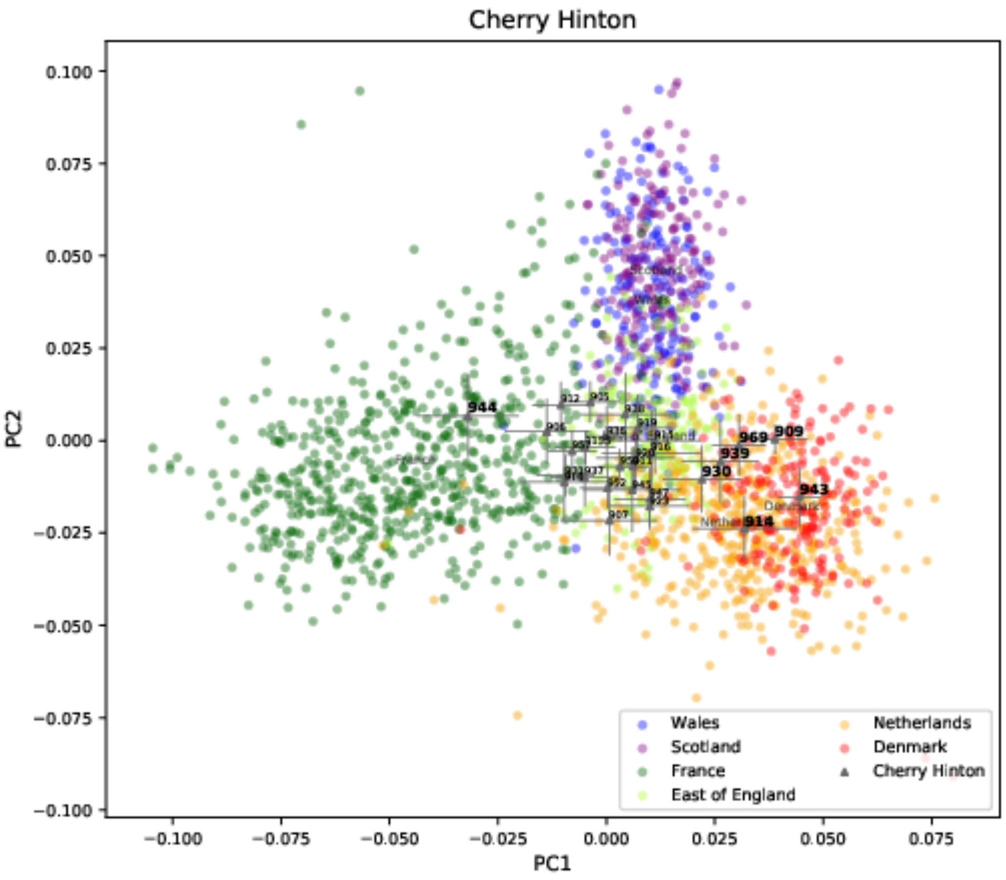

Fig. S2: PCA of West Europe individuals from UK Biobank, showing lsq-projection of historical genomes from Cherry Hinton. Only genomes > 0.05× are included; error bars represent one standard deviation estimated from 20-fold block jackknife; outliers labelled in bold.

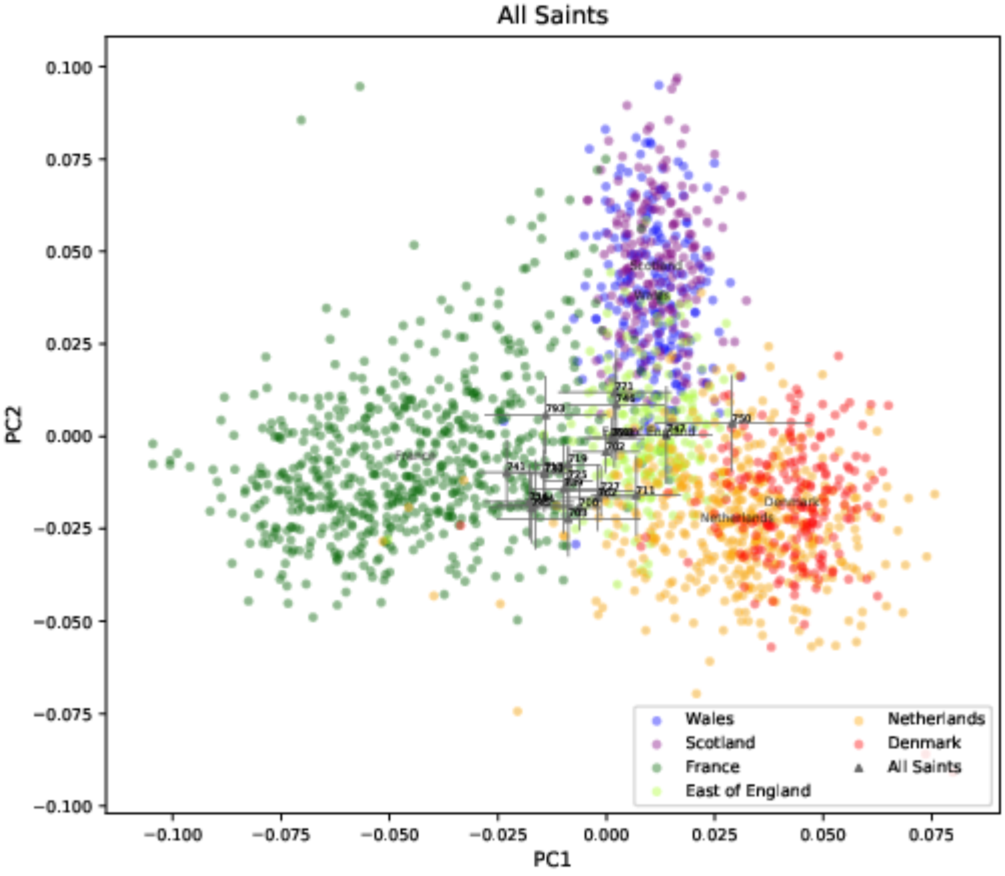

Fig. S3: PCA of West Europe individuals from UK Biobank, showing lsq-projection of historical genomes from All Saints by the Castle. Only genomes > 0.05x are included; error bars represent one standard deviation estimated from 20-fold block jackknife.

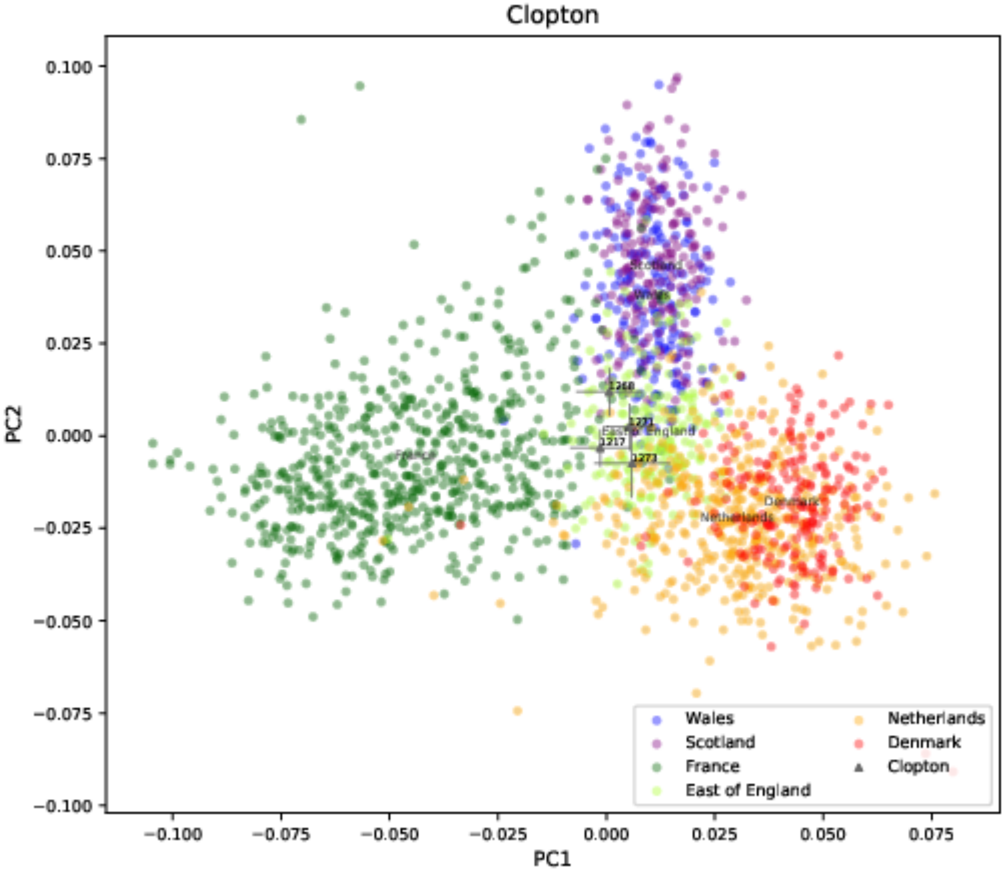

Fig. S4: PCA of West Europe individuals from UK Biobank, showing lsq-projection of historical genomes from Clopton. Only genomes > 0.05× are included; error bars represent one standard deviation estimated from 20-fold block jackknife.

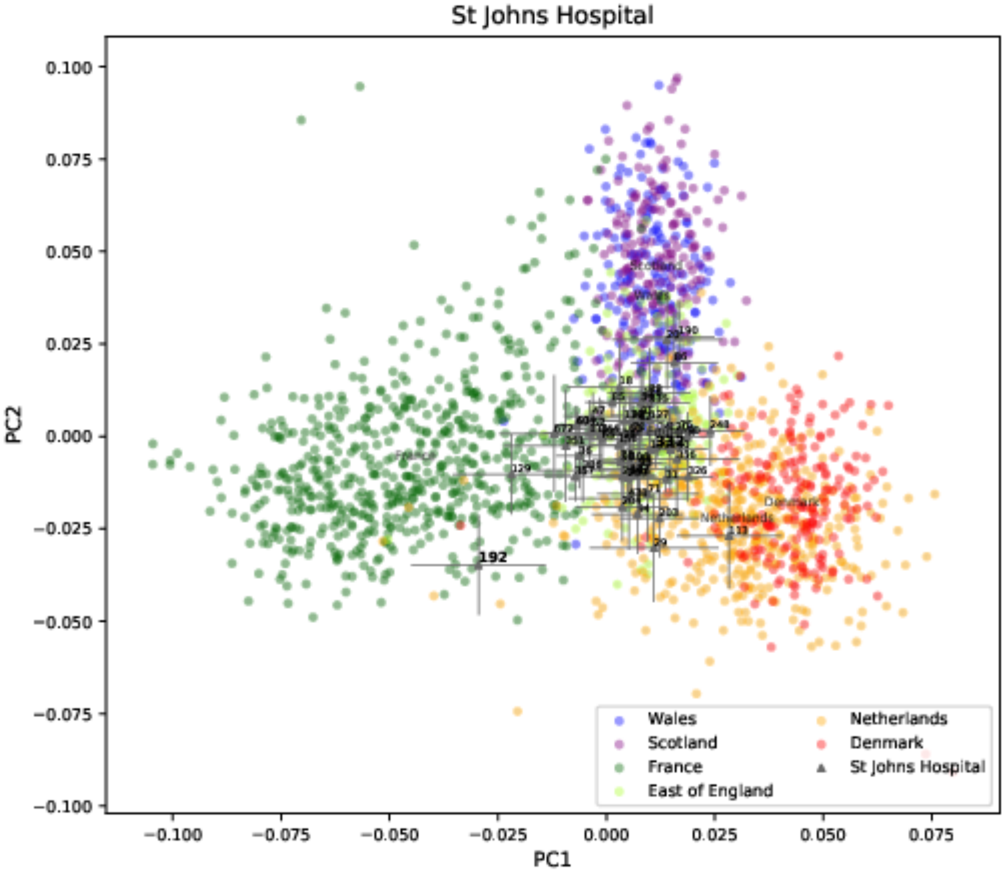

Fig. S5: PCA of West Europe individuals from UK Biobank, showing lsq-projection of historical genomes from the Hospital of St John. Only genomes > 0.05x are included; error bars represent one standard deviation estimated from 20-fold block jackknife; outliers labelled in bold.

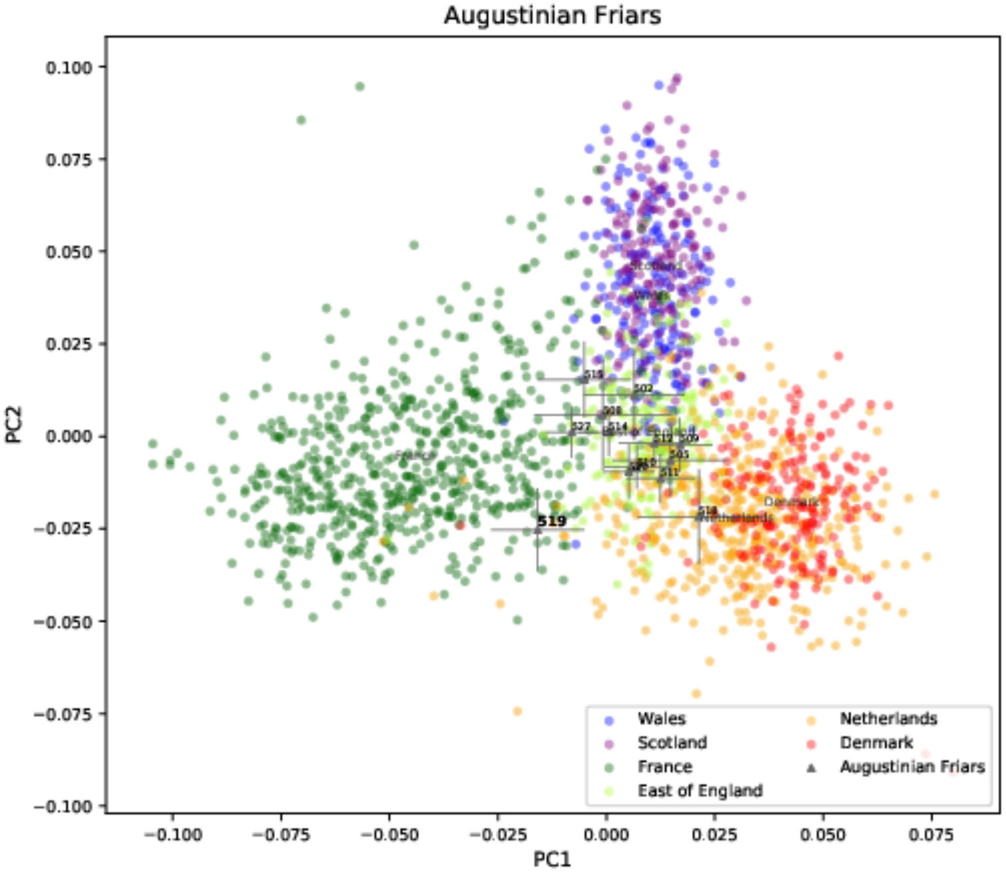

Fig. S6: PCA of West Europe individuals from UK Biobank, showing lsq-projection of historical genomes from Augustinian Friary. Only genomes > 0.05× are included; error bars represent one standard deviation estimated from 20-fold block jackknife; outliers labelled in bold.

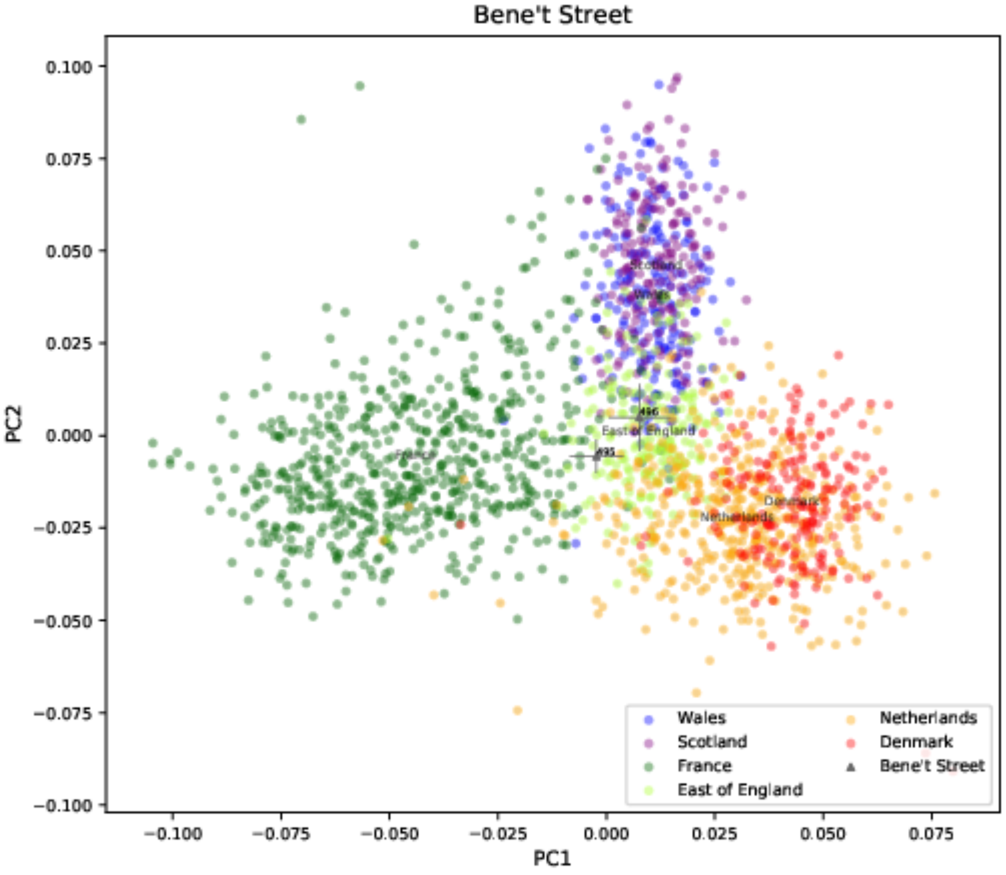

Fig. S7: PCA of West Europe individuals from UK Biobank, showing lsq-projection of historical genomes from Bene’t Street. Only genomes > 0.05× are included; error bars represent one standard deviation estimated from 20-fold block jackknife.

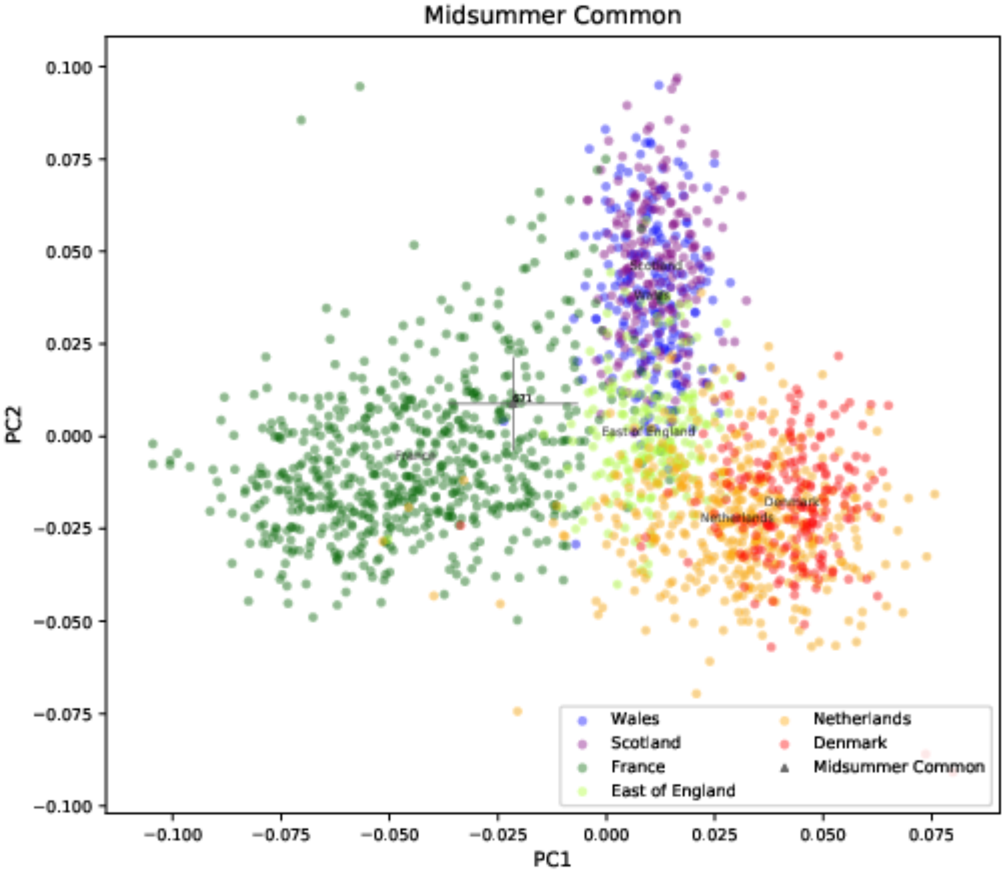

Fig. S8: PCA of West Europe individuals from UK Biobank, showing lsq-projection of historical genomes from Midsummer Common. Only genomes > 0.05x are included; error bars represent one standard deviation estimated from 20-fold block jackknife.

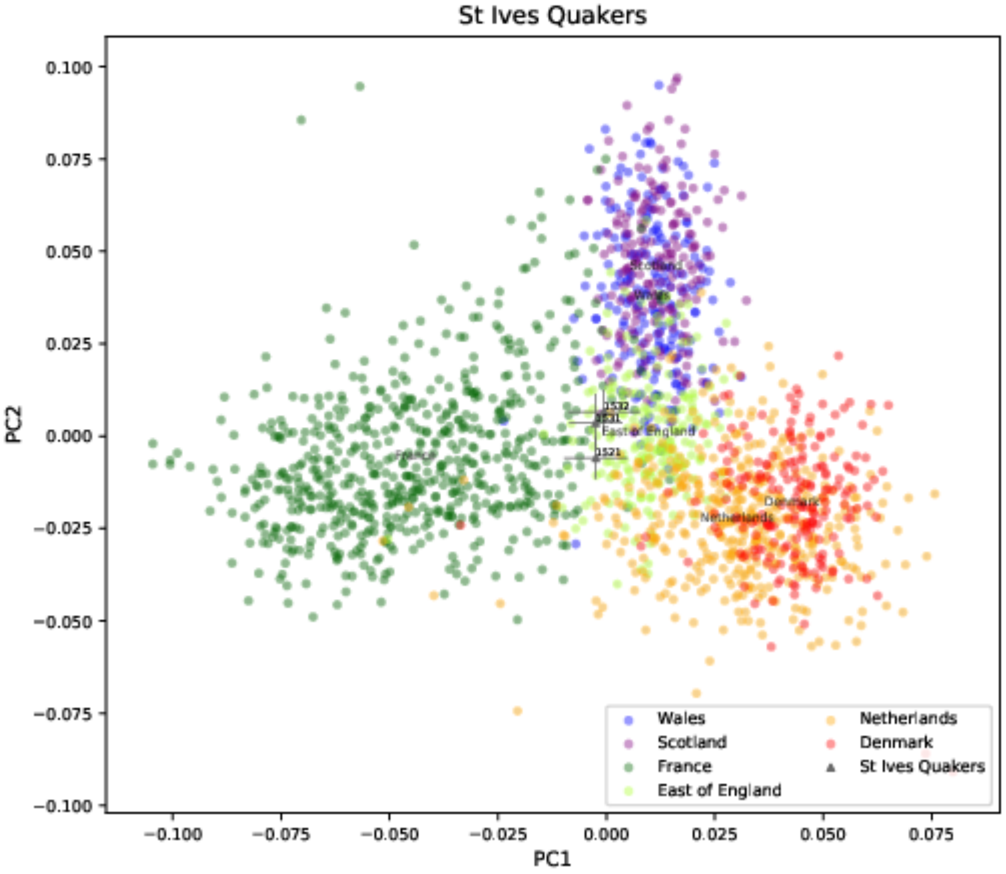

Fig. S9: PCA of West Europe individuals from UK Biobank, showing lsq-projection of historical genomes from Hemingford Gray Quakers. Only genomes > 0.05× are included; error bars represent one standard deviation estimated from 20-fold block jackknife.

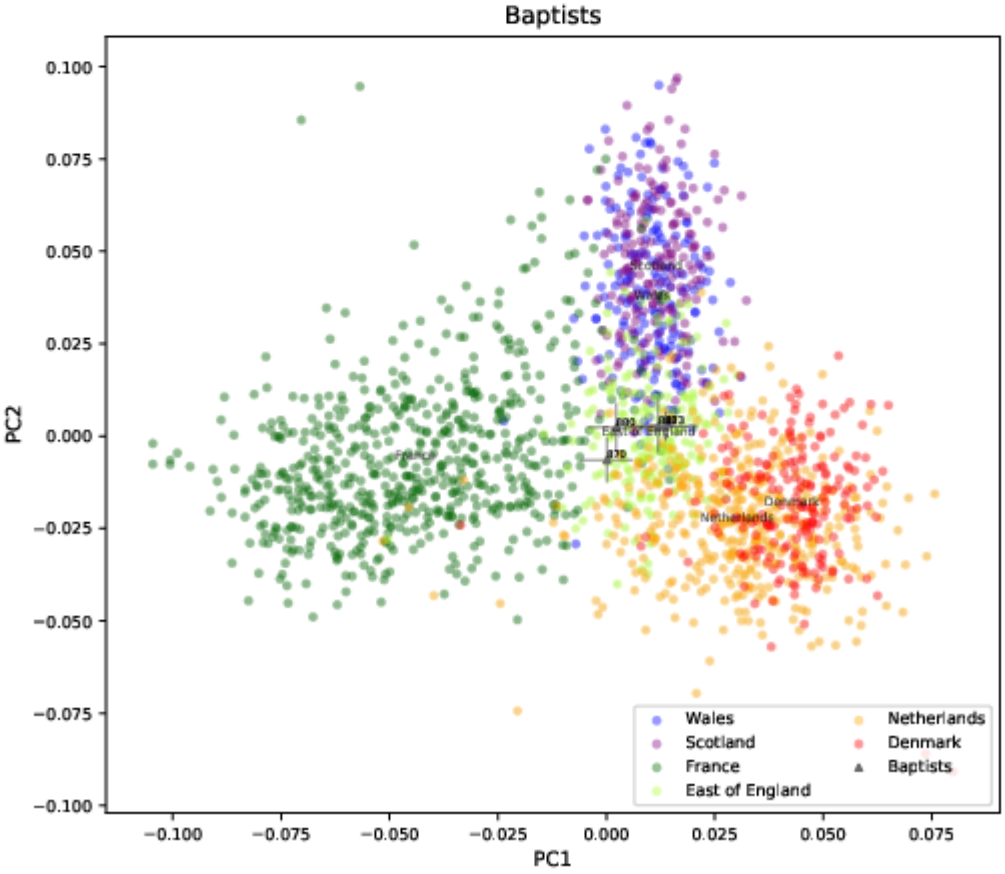

Fig. S10: PCA of west Europe individuals from UK Biobank, showing lsq-projection of historical genomes from Providence Calvinistic Baptist Chapel. Only genomes > 0.05x are included; error bars represent one standard deviation estimated from 20-fold block jackknife.

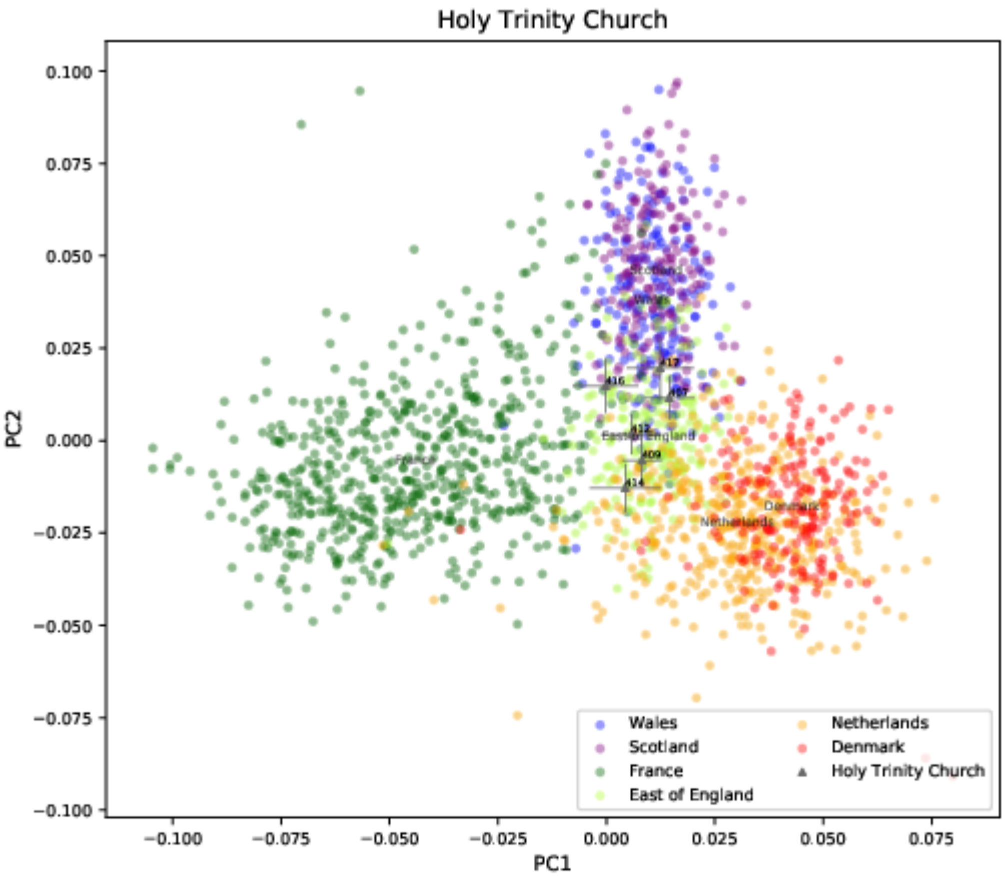

Fig. S11: PCA of west Europe individuals from UK Biobank, showing lsq-projection of historical genomes from Holy Trinity Church. Only genomes > 0.05× are included; error bars represent one standard deviation estimated from 20-fold block jackknife.

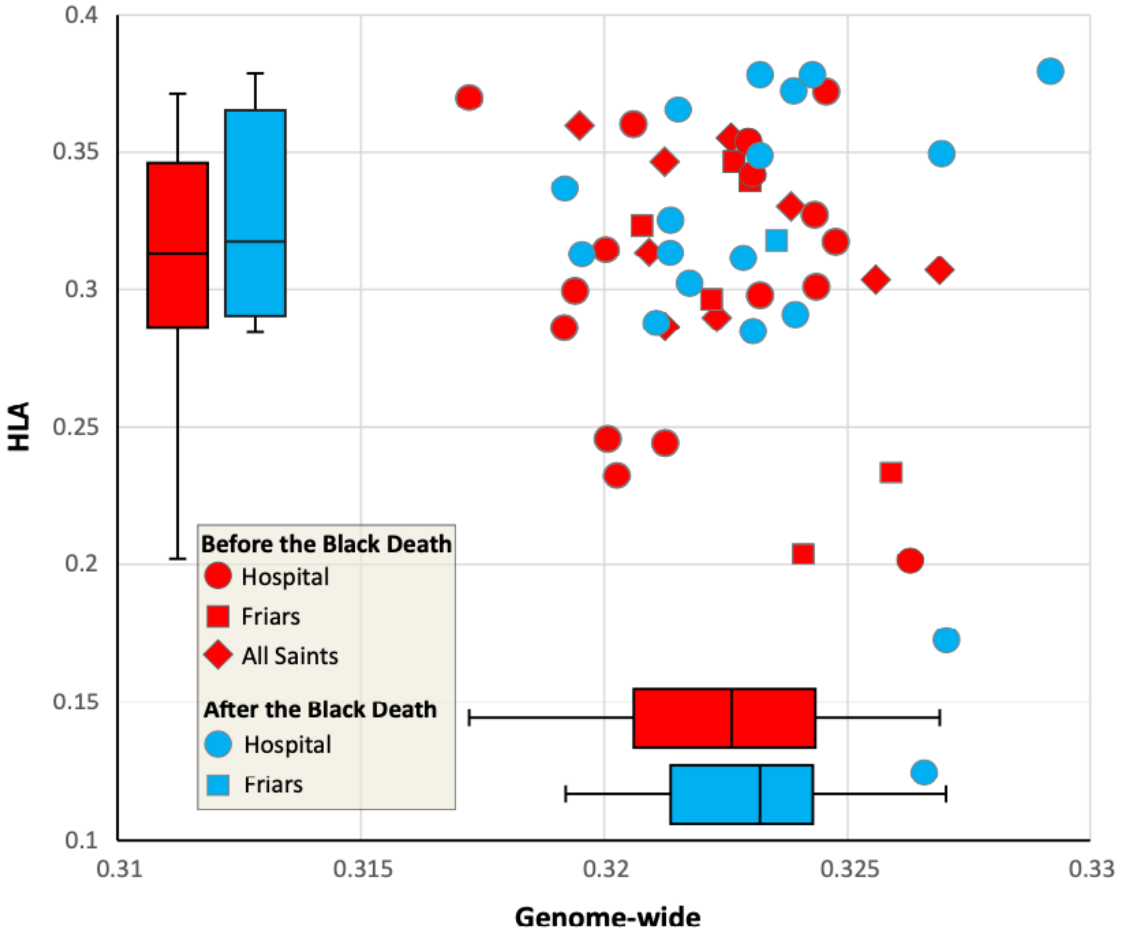

Fig. S12. Heterozygosity of the medieval Cambridge genomes from before and after the Black Death. Average heterozygosity estimates for 5.4 million variants with MAF > 0.05 were obtained from imputed genotypes of 50 genomes with coverage > 0.1× from 4 sites in Cambridge.

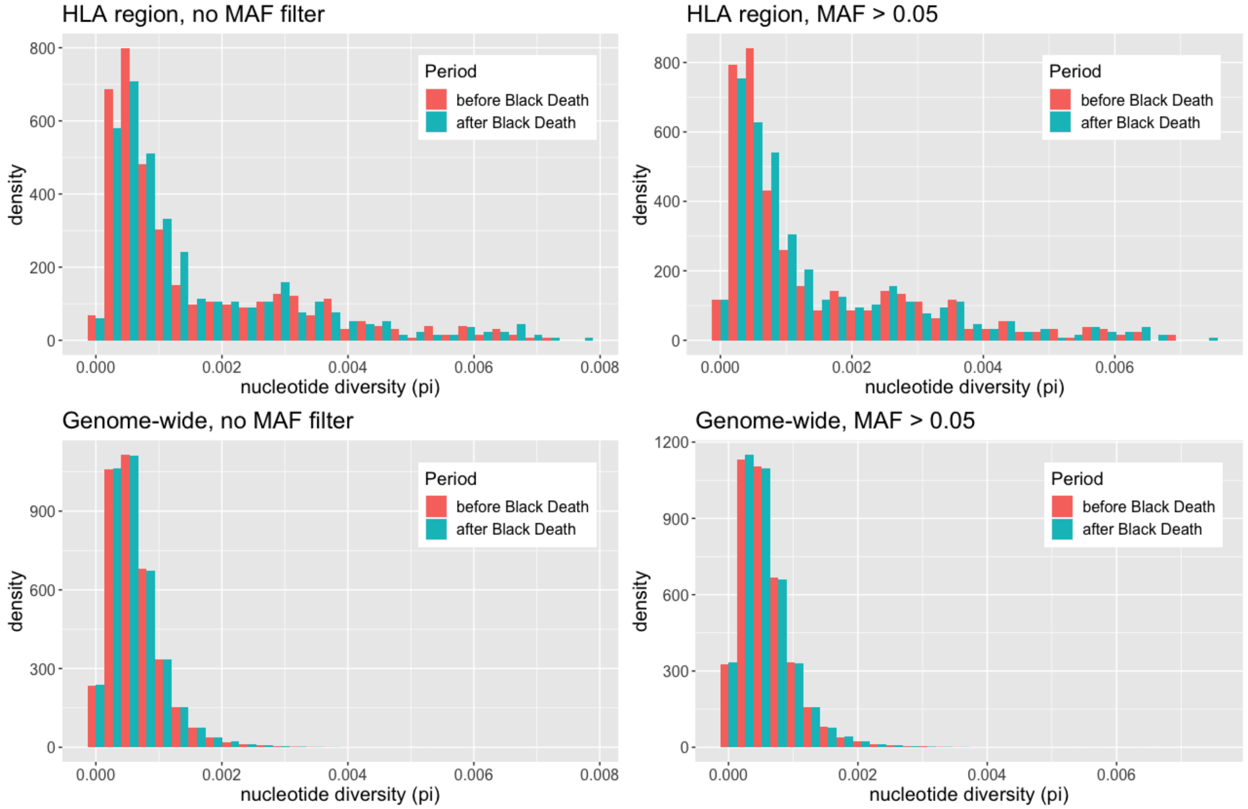

Fig. S13. Histograms comparing nucleotide diversity of the medieval Cambridge genomes from before and after the Black Death.

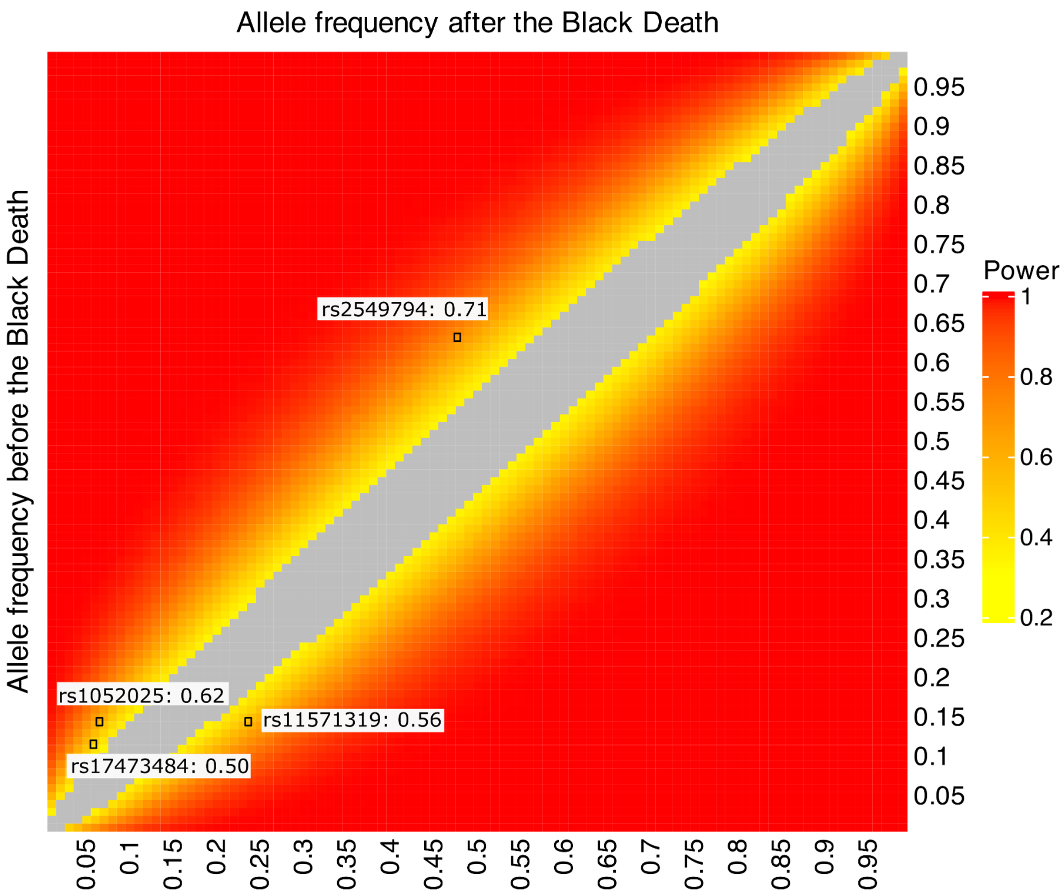

Fig. S14. The power to observe F_{ST} values greater than 0.0089, the 95 percentile reported by Klunk *et al.* (*16*) among neutral variants, based on the sample size of the Cambridge dataset for each combination of allele frequencies before and after the Black Death. The populations are assumed to be in Hardy-Weinberg equilibrium. The four variants highlighted by Klunk *et al.* are labeled according to the frequencies reported in their London cohort before and after the Black Death.

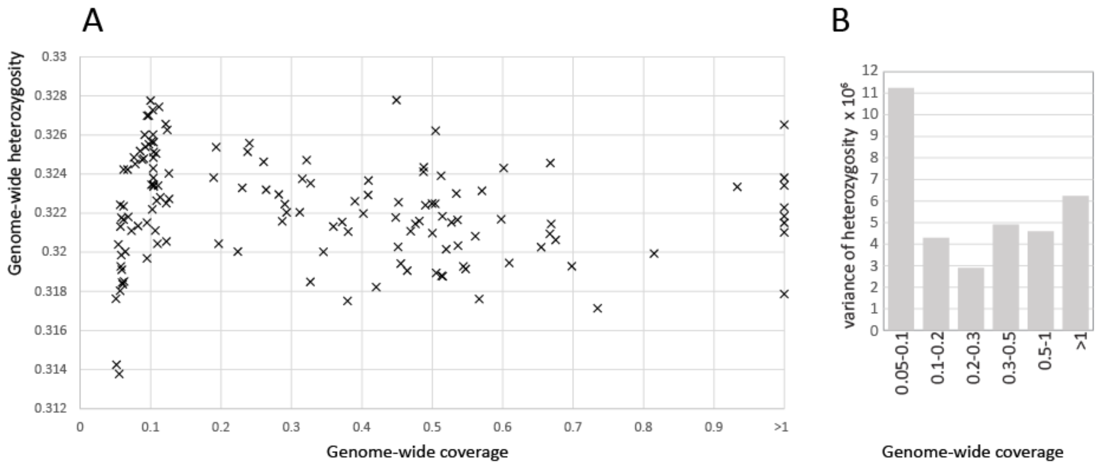

Fig. S15. Relationship between heterozygosity and coverage in imputed ancient genomes. A. Scatter plot of individual heterozygosity and coverage values of imputed genomes. Three individuals (PSN357, PSN412, PSN870) with long runs of homozygosity were excluded. B. Variance of heterozygosity estimates in genomes grouped by their coverage.

Supplementary Tables

Table S1. Sequence data summary for the newly reported later medieval and post-medieval genomes.

Table S2. Mitochondrial DNA haplogroup frequencies in Cambridge(shire) through historic time in context of modern and prehistoric data from across Britain.

Table S3. Average connectedness (PiC7cM) between medieval and postmedieval residents Cambridgeshire and modern genomes of the UK Biobank.

Table S4. Allele frequency of the phenotype informative SNPs by time period and, for medieval samples in this study, by relative time to the Second Pandemic (before or after).

Table S5. Sample-by-sample genotypes for the SNPs involved in the pigmentation prediction (eye, hair and skin colour) from the HIrisPlex-S set. Genotypes are reported in the form of counts of effective alleles.

Table S6. Sample-by-sample genotypes for the other SNPs involved diet and disease. Genotypes are reported in the form of counts of effective alleles.

Table S7. Kinship among medieval, post-medieval and modern genomes from the UK Biobank.

Table S8. Runs of homozygosity estimated from imputed genomes (coverage > 0.1x) using hapROH.

Table S9. Overlap of highly differentiated immunity gene variants between London and Cambridge cohorts.

Table S10. Enrichment of highly differentiated immunity variants in the Cambridge pre- and post-plague cohorts.

Table S11. Imputation accuracy evaluated by downsampling PSN31.

REFERENCES AND NOTES

1. P. Skoglund, I. Mathieson, Ancient genomics of modern humans: The first decade. *Annu. Rev. Genomics Hum. Genet.* **19**, 381–404 (2018).

2. L. Orlando, R. Allaby, P. Skoglund, C. Der Sarkissian, P. W. Stockhammer, M. C. Ávila-Arcos, Q. Fu, J. Krause, E. Willerslev, A. C. Stone, C. Warinner, Ancient DNA analysis. *Nat. Rev. Methods Primer* **1**, 14 (2021).

3. J. M. Kuhn, M. Jakobsson, T. Günther, Estimating genetic kin relationships in prehistoric populations. *PLOS ONE* **13**, e0195491 (2018).

4. L. M. Cassidy, R. Ó. Maoldúin, T. Kador, A. Lynch, C. Jones, P. C. Woodman, E. Murphy, G. Ramsey, M. Dowd, A. Noonan, C. Campbell, E. R. Jones, V. Mattiangeli, D. G. Bradley, A dynastic elite in monumental Neolithic society. *Nature* **582**, 384–388 (2020).

5. H. Ringbauer, J. Novembre, M. Steinrücken, Parental relatedness through time revealed by runs of homozygosity in ancient DNA. *Nat. Commun.* **12**, 5425 (2021).

6. C. Warinner, A. Herbig, A. Mann, J. A. Fellows Yates, C. L. Weiß, H. A. Burbano, L. Orlando, J. Krause, A Robust Framework for Microbial Archaeology. *Annu. Rev. Genomics Hum. Genet.* **18**, 321–356 (2017).

7. M. A. Spyrou, K. I. Bos, A. Herbig, J. Krause, Ancient pathogen genomics as an emerging tool for infectious disease research. *Nat. Rev. Genet.* **20**, 323–340 (2019).

8. S. Marciniak, G. H. Perry, Harnessing ancient genomes to study the history of human adaptation. *Nat. Rev. Genet.* **18**, 659–674 (2017).

9. E. K. Irving-Pease, R. Muktupavela, M. Dannemann, F. Racimo, Quantitative human paleogenetics: What can ancient DNA tell us about complex trait evolution? *Front. Genet.* **12**, 703541 (2021).

10. C. Cessford, C. L. Scheib, M. Guellil, M. Keller, C. Alexander, S. A. Inskip, J. E. Robb, Beyond Plague Pits: Using genetics to identify responses to plague in medieval cambridgeshire. *Eur. J. Archaeol.* **24**, 496–518 (2021).

11. O. J. Benedictow, *The Complete History of the Black Death* (Boydell & Brewer, 2021).

12. R. Horrox, Ed., *The Black Death* (Manchester Medieval Sources series, Manchester Univ. Press, Manchester, reprint., 1995).

13. A. Dumay, O. Gergaud, M. Roy, J.-P. Hugot, Is Crohn's Disease the price to pay today for having survived the black death? *J. Crohns Colitis* **13**, 1318–1322 (2019).

14. A. Immel, F. M. Key, A. Szolek, R. Barquera, M. K. Robinson, G. F. Harrison, W. H. Palmer, M. A. Spyrou, J. Susat, B. Krause-Kyora, K. I. Bos, S. Forrest, D. I. Hernández-Zaragoza, J. Sauter, U. Solloch, A. H. Schmidt, V. J. Schuenemann, E. Reiter, M. S. Kairies, R. Weiß, S. Arnold, J. Wahl, J. A. Hollenbach, O. Kohlbacher, A. Herbig, P. J. Norman, J. Krause, Analysis of genomic DNA from medieval plague victims suggests long-term effect of Yersinia pestis on human immunity genes. *Mol. Biol. Evol.* **38**, 4059–4076 (2021).

15. S. Gopalakrishnan, S. S. Ebenesersdóttir, I. K. C. Lundstrøm, G. Turner-Walker, K. H. S. Moore, P. Luisi, A. Margaryan, M. D. Martin, M. R. Ellegaard, Ó. Þ. Magnússon, Á. Sigurðsson, S. Snorradóttir, D. N. Magnúsdóttir, J. E. Laffoon, L. van Dorp, X. Liu, I. Moltke, M. C. Ávila-Arcos, J. G. Schraiber, S. Rasmussen, D. Juan, P. Gelabert, T. de-Dios, A. K. Fotakis, M. Iraeta-Orbegozo, Å. J. Vågene, S. D. Denham, A. Christophersen, H. K. Stenøien, F. G. Vieira, S. Liu, T. Günther, T. Kivisild, O. G. Moseng, B. Skar, C. Cheung, M. Sandoval-Velasco, N. Wales, H. Schroeder, P. F. Campos, V. B. Guðmundsdóttir, T. Sicheritz-Ponten, B. Petersen, J. Halgunset, E. Gilbert, G. L. Cavalleri, E. Hovig, I. Kockum, T. Olsson, L. Alfredsson, T. F. Hansen, T. Werge, E. Willerslev, F. Balloux, T. Marques-Bonet, C. Lalueza-Fox, R. Nielsen, K. Stefánsson, A. Helgason, M. T. P Gilbert, The population genomic legacy of the second plague pandemic. *Curr. Biol.* **32**, 4743–4751.e6 (2022).

16. J. Klunk, T. P. Vilgalys, C. E. Demeure, X. Cheng, M. Shiratori, J. Madej, R. Beau, D. Elli, M. I. Patino, R. Redfern, S. N. DeWitte, J. A. Gamble, J. L. Boldsen, A. Carmichael, N. Varlik, K. Eaton, J.-C. Grenier, G. B. Golding, A. Devault, J.-M. Rouillard, V. Yotova, R. Sindeaux, C. J. Ye, M. Bikaran, A. Dumaine, J. F. Brinkworth, D. Missiakas, G. A. Rouleau, M. Steinrücken, J. Pizarro-Cerdá, H. N. Poinar, L. B. Barreiro, Evolution of immune genes is associated with the Black Death. *Nature* **611**, 312–319 (2022).

17. S. N. DeWitte, J. W. Wood, Selectivity of Black Death mortality with respect to preexisting health. *Proc. Natl. Acad. Sci. U. S. A.* **105**, 1436–1441 (2008).

18. K. Godde, V. Pasillas, A. Sanchez, Survival analysis of the Black Death: Social inequality of women and the perils of life and death in Medieval London. *Am. J. Phys. Anthropol.* **173**, 168–178 (2020).

19. D. Herlihy, S. K. Cohn, *The Black Death and the Transformation of the West* (Harvard Univ. Press, 1997).

20. B. M. S. Campbell, *The Great Transition: Climate, Disease and Society in the Late-Medieval World* (Cambridge Univ. Press, ed. 1, 2016).

21. R. Martiniano, A. Caffell, M. Holst, K. Hunter-Mann, J. Montgomery, G. Müldner, R. L. McLaughlin, M. D. Teasdale, W. Van Rheenen, J. H. Veldink, L. H. Van Den Berg, O. Hardiman, M. Carroll, S. Roskams, J. Oxley, C. Morgan, M. G. Thomas, I. Barnes, C. McDonnell, M. J. Collins, D. G. Bradley, Genomic signals of migration and continuity in Britain before the Anglo-Saxons. *Nat. Commun.* **7**, 10326 (2016).

22. S. Schiffels, W. Haak, P. Paajanen, B. Llamas, E. Popescu, L. Loe, R. Clarke, A. Lyons, R. Mortimer, D. Sayer, C. Tyler-Smith, A. Cooper, R. Durbin, Iron Age and Anglo-Saxon genomes from East England reveal British migration history. *Nat. Commun.* **7**, 10408 (2016).

23. A. Rose, *Life in Medieval Cambridge: An Isotopic Analysis of Diet and Mobility*. (University of Cambridge, 2020).

24. J. Evans, C. Chenery, K. Mee, C. Cartwright, K. Lee, B. G. S. Andrew Marchant, B. G. S. Lina Hannaford, Biosphere Isotope Domains GB (V1): Interactive Website (2018).

25. D. N. Seidman, S. A. Shenoy, M. Kim, R. Babu, I. G. Woods, T. D. Dyer, D. M. Lehman, J. E. Curran, R. Duggirala, J. Blangero, A. L. Williams, Rapid, phase-free detection of long identity-by-descent segments enables effective relationship classification. *Am. J. Hum. Genet.* **106**, 453–466 (2020).

26. T. Kivisild, L. Saag, R. Hui, S. A. Biagini, V. Pankratov, E. D'Atanasio, L. Pagani, L. Saag, S. Rootsi, R. Mägi, E. Metspalu, H. Valk, M. Malve, K. Irdt, T. Reisberg, A. Solnik, C. L. Scheib, D. N. Seidman, A. L. Williams, K. Tambets, M. Metspalu, Patterns of genetic connectedness between modern and medieval Estonian genomes reveal the origins of a major ancestry component of the Finnish population. *Am. J. Hum. Genet.* **108**, 1792–1806 (2021).

27. W. M. Ormrod, B. Lambert, J. Mackman, *Immigrant England, 1300-1550* (Manchester Medieval Studies, Manchester Univ. Press, 2018).

28. W. M. Ormrod, J. Story, E. M. Tyler, Eds., *Migrants in Medieval England, c. 500-c. 1500* (Proceedings of the British Academy, Oxford Univ. Press, ed. 1, 2020).

29. S. Leslie, B. Winney, G. Hellenthal, D. Davison, A. Boumertit, T. Day, K. Hutnik, E. C. Royrvik, B. Cunliffe, D. J. Lawson, D. Falush, C. Freeman, M. Pirinen, S. Myers, M. Robinson, P. Donnelly, W. Bodmer, The fine-scale genetic structure of the British population. *Nature* **519**, 309–314 (2015).

30. A. Margaryan, D. J. Lawson, M. Sikora, F. Racimo, S. Rasmussen, I. Moltke, L. M. Cassidy, E. Jørsboe, A. Ingason, M. W. Pedersen, T. Korneliussen, H. Wilhelmson, M. M. Buś, P. de Barros Damgaard, R. Martiniano, G. Renaud, C. Bhérer, J. V. Moreno-Mayar, A. K. Fotakis, M. Allen, R. Allmäe, M. Molak, E. Cappellini, G. Scorrano, H. McColl, A. Buzhilova, A. Fox, A. Albrechtsen, B. Schütz, B. Skar, C. Arcini, C. Falys, C. H. Jonson, D. Błaszczyk, D. Pezhemsky, G. Turner-Walker, H. Gestsdóttir, I. Lundstrøm, I. Gustin, I. Mainland, I. Potekhina, I. M. Muntoni, J. Cheng, J. Stenderup, J. Ma, J. Gibson, J. Peets, J. Gustafsson, K. H. Iversen, L. Simpson, L. Strand, L. Loe, M. Sikora, M. Florek, M. Vretemark, M. Redknap, M. Bajka, T. Pushkina, M. Søvsø, N. Grigoreva, T. Christensen, O. Kastholm, O. Uldum, P. Favia, P. Holck, S. Sten, S. V. Arge, S. Ellingvåg, V. Moiseyev, W. Bogdanowicz, Y. Magnusson, L. Orlando, P. Pentz, M. D. Jessen, A. Pedersen, M. Collard, D. G. Bradley, M. L. Jørkov, J. Arneborg, N. Lynnerup, N. Price, M. T. P. Gilbert, M. E. Allentoft, J. Bill, S. M. Sindbæk, L. Hedeager, K. Kristiansen, R. Nielsen, T. Werge, E. Willerslev, Population genomics of the Viking world. *Nature* **585**, 390–396 (2020).

31. D. A. van Heel, L. Franke, K. A. Hunt, R. Gwilliam, A. Zhernakova, M. Inouye, M. C. Wapenaar, M. C. N. M. Barnardo, G. Bethel, G. K. T. Holmes, C. Feighery, D. Jewell, D. Kelleher, P. Kumar, S. Travis, J. R. Walters, D. S. Sanders, P. Howdle, J. Swift, R. J. Playford, W. M. McLaren, M. L.

Mearin, C. J. Mulder, R. McManus, R. McGinnis, L. R. Cardon, P. Deloukas, C. Wijmenga, A genome-wide association study for celiac disease identifies risk variants in the region harboring IL2 and IL21. *Nat. Genet.* **39**, 827–829 (2007).

32. A. Zhernakova, B. Z. Alizadeh, M. Bevova, M. A. van Leeuwen, M. J. H. Coenen, B. Franke, L. Franke, M. D. Posthumus, D. A. van Heel, G. van der Steege, T. R. D. J. Radstake, P. Barrera, B. O. Roep, B. P. C. Koeleman, C. Wijmenga, Novel association in chromosome 4q27 region with rheumatoid arthritis and confirmation of Type 1 diabetes point to a general risk locus for autoimmune diseases. *Am. J. Hum. Genet.* **81**, 1284–1288 (2007).

33. S. Pabst, T. Fränken, J. Schönau, S. Stier, G. Nickenig, R. Meyer, D. Skowasch, C. Grohé, Transforming growth factor-β gene polymorphisms in different phenotypes of sarcoidosis. *Eur. Respir. J.* **38**, 169–175 (2011).

34. L. Chaitanya, K. Breslin, S. Zuñiga, L. Wirken, E. Pośpiech, M. Kukla-Bartoszek, T. Sijen, P. de Knijff, F. Liu, W. Branicki, M. Kayser, S. Walsh, The HIrisPlex-S system for eye, hair and skin colour prediction from DNA: Introduction and forensic developmental validation. *Forensic Sci. Int. Genet.* **35**, 123–135 (2018).

35. M. Keller, M. Guellil, P. Slavin, L. Saag, K. Irdt, H. Niinemäe, A. Solnik, M. Malve, H. Valk, A. Kriiska, C. Cessford, S. A. Inskip, J. E. Robb, C. Cooper, C. von Planta, M. Seifert, T. Reitmaier, W. A. Baetsen, D. Walker, S. Lösch, S. Szidat, M. Metspalu, T. Kivisild, K. Tambets, C. L. Scheib, A refined phylochronology of the second plague pandemic in Western Eurasia. bioRxiv 2023.07.18.549544 [**Preprint**]. 19 July 2023. https://doi.org/10.1101/2023.07.18.549544.

36. M. A. Spyrou, M. Keller, R. I. Tukhbatova, C. L. Scheib, E. A. Nelson, A. Andrades Valtueña, G. U. Neumann, D. Walker, A. Alterauge, N. Carty, C. Cessford, H. Fetz, M. Gourvennec, R. Hartle, M. Henderson, K. von Heyking, S. A. Inskip, S. Kacki, F. M. Key, E. L. Knox, C. Later, P. Maheshwari-Aplin, J. Peters, J. E. Robb, J. Schreiber, T. Kivisild, D. Castex, S. Lösch, M. Harbeck, A. Herbig, K. I. Bos, J. Krause, Phylogeography of the second plague pandemic revealed through analysis of historical Yersinia pestis genomes. *Nat. Commun.* **10**, 4470 (2019).

37. F.-R. Zhang, W. Huang, S.-M. Chen, L.-D. Sun, H. Liu, Y. Li, Y. Cui, X.-X. Yan, H.-T. Yang, Rong-De Yang, T.-S. Chu, C. Zhang, L. Zhang, J.-W. Han, G.-Q. Yu, C. Quan, Y.-X. Yu, Z. Zhang, B.-Q. Shi, L.-H. Zhang, H. Cheng, C.-Y. Wang, Y. Lin, H.-F. Zheng, X.-A. Fu, X.-B. Zuo, Q. Wang, H. Long, Y.-P. Sun, Y.-L. Cheng, H.-Q. Tian, F.-S. Zhou, H.-X. Liu, W.-S. Lu, S.-M. He, W.-L. Du, M. Shen, Q.-Y. Jin, Y. Wang, H.-Q. Low, T. Erwin, N.-H. Yang, J.-Y. Li, X. Zhao, Y.-L. Jiao, L.-G. Mao, G. Yin, Z.-X. Jiang, X.-D. Wang, J.-P. Yu, Z.-H. Hu, C.-H. Gong, Y.-Q. Liu, R.-Y. Liu, D.-M. Wang, D. Wei, J.-X. Liu, W.-K. Cao, H.-Z. Cao, Y.-P. Li, W.-G. Yan, S.-Y. Wei, K.-J. Wang, M. L. Hibberd, S. Yang, X.-J. Zhang, J.-J. Liu, Genomewide association study of leprosy. *N. Engl. J. Med.* **361**, 2609–2618 (2009).

38. I. Gronau, M. J. Hubisz, B. Gulko, C. G. Danko, A. Siepel, Bayesian inference of ancient human demography from individual genome sequences. *Nat. Genet.* **43**, 1031–1034 (2011).

39. S. McCarthy, S. Das, W. Kretzschmar, O. Delaneau, A. R. Wood, A. Teumer, H. M. Kang, C. Fuchsberger, P. Danecek, K. Sharp, Y. Luo, C. Sidore, A. Kwong, N. Timpson, S. Koskinen, S. Vrieze, L. J. Scott, H. Zhang, A. Mahajan, J. Veldink, U. Peters, C. Pato, C. M. Van Duijn, C. E. Gillies, I. Gandin, M. Mezzavilla, A. Gilly, M. Cocca, M. Traglia, A. Angius, J. C. Barrett, D. Boomsma, K. Branham, G. Breen, C. M. Brummett, F. Busonero, H. Campbell, A. Chan, S. Chen, E. Chew, F. S. Collins, L. J. Corbin, G. D. Smith, G. Dedoussis, M. Dorr, A. E. Farmaki, L. Ferrucci, L. Forer, R. M. Fraser, S. Gabriel, S. Levy, L. Groop, T. Harrison, A. Hattersley, O. L. Holmen, K. Hveem, M. Kretzler, J. C. Lee, M. McGue, T. Meitinger, D. Melzer, J. L. Min, K. L. Mohlke, J. B. Vincent, M. Nauck, D. Nickerson, A. Palotie, M. Pato, N. Pirastu, M. McInnis, J. B. Richards, C. Sala, V. Salomaa, D. Schlessinger, S. Schoenherr, P. E. Slagboom, K. Small, T. Spector, D. Stambolian, M. Tuke, J. Tuomilehto, L. H. Van Den Berg, W. Van Rheenen, U. Volker, C. Wijmenga, D. Toniolo, E. Zeggini, P. Gasparini, M. G. Sampson, J. F. Wilson, T. Frayling, P. I. W. De Bakker, M. A. Swertz, S. McCarroll, C. Kooperberg, A. Dekker, D. Altshuler, C. Willer, W. Iacono, S. Ripatti, N. Soranzo, K. Walter, A. Swaroop, F. Cucca, C. A. Anderson, R. M. Myers, M. Boehnke, M. I. McCarthy, R. Durbin, G. Abecasis, J. Marchini, A reference panel of 64,976 haplotypes for genotype imputation. *Nat. Genet.* **48**, 1279–1283 (2016).

40. J. Gretzinger, D. Sayer, P. Justeau, E. Altena, M. Pala, K. Dulias, C. J. Edwards, S. Jodoin, L. Lacher, S. Sabin, Å. J. Vågene, W. Haak, S. S. Ebenesersdóttir, K. H. S. Moore, R. Radzeviciute,

K. Schmidt, S. Brace, M. A. Bager, N. Patterson, L. Papac, N. Broomandkhoshbacht, K. Callan, É. Harney, L. Iliev, A. M. Lawson, M. Michel, K. Stewardson, F. Zalzala, N. Rohland, S. Kappelhoff-Beckmann, F. Both, D. Winger, D. Neumann, L. Saalow, S. Krabath, S. Beckett, M. Van Twest, N. Faulkner, C. Read, T. Barton, J. Caruth, J. Hines, B. Krause-Kyora, U. Warnke, V. J. Schuenemann, I. Barnes, H. Dahlström, J. J. Clausen, A. Richardson, E. Popescu, N. Dodwell, S. Ladd, T. Phillips, R. Mortimer, F. Sayer, D. Swales, A. Stewart, D. Powlesland, R. Kenyon, L. Ladle, C. Peek, S. Grefen-Peters, P. Ponce, R. Daniels, C. Spall, J. Woolcock, A. M. Jones, A. V. Roberts, R. Symmons, A. C. Rawden, A. Cooper, K. I. Bos, T. Booth, H. Schroeder, M. G. Thomas, A. Helgason, M. B. Richards, D. Reich, J. Krause, S. Schiffels, The Anglo-Saxon migration and the formation of the early English gene pool. *Nature* **610**, 112–119 (2022).

41. J. Klunk, A. T. Duggan, R. Redfern, J. Gamble, J. L. Boldsen, G. B. Golding, B. S. Walter, K. Eaton, J. Stangroom, J. M. Rouillard, A. Devault, S. N. DeWitte, H. N. Poinar, Genetic resiliency and the Black Death: No apparent loss of mitogenomic diversity due to the Black Death in medieval London and Denmark. *Am. J. Phys. Anthropol.* **169**, 240–252 (2019).

42. M. Lewis, J. Montgomery, Youth mobility, migration, and health before and after the black death. *Bioarchaeology Int.* **7**, 111–129 (2023).

43. G. Caserta, Considerations about the marriage regulations of Canon Law and the apostolic penitentiary in late Middle Ages. *Ius Canonicum,* **XLVII**, 119–139 (2007).

44. J. C. Stephens, D. E. Reich, D. B. Goldstein, H. D. Shin, M. W. Smith, M. Carrington, C. Winkler, G. A. Huttley, R. Allikmets, L. Schriml, B. Gerrard, M. Malasky, M. D. Ramos, S. Morlot, M. Tzetis, C. Oddoux, F. S. di Giovine, G. Nasioulas, D. Chandler, M. Aseev, M. Hanson, L. Kalaydjieva, D. Glavac, P. Gasparini, E. Kanavakis, M. Claustres, M. Kambouris, H. Ostrer, G. Duff, V. Baranov, H. Sibul, A. Metspalu, D. Goldman, N. Martin, D. Duffy, J. Schmidtke, X. Estivill, S. J. O'Brien, M. Dean, Dating the origin of the *CCR5*-Δ32 AIDS-resistance Allele by the coalescence of haplotypes. *Am. J. Hum. Genet.* **62**, 1507–1515 (1998).

45. D. Y. C. Brandt, J. César, J. Goudet, D. Meyer, The effect of balancing selection on population differentiation: A study with HLA genes. *G3 (Bethesda)* **8**, 2805–2815 (2018).

46. Y. Souilmi, R. Tobler, A. Johar, M. Williams, S. T. Grey, J. Schmidt, J. C. Teixeira, A. Rohrlach, J. Tuke, O. Johnson, G. Gower, C. Turney, M. Cox, A. Cooper, C. D. Huber, Admixture has obscured signals of historical hard sweeps in humans. *Nat. Ecol. Evol.* **6**, 2003–2015 (2022).

47. B. Ferwerda, M. B. B. McCall, M. C. de Vries, J. Hopman, B. Maiga, A. Dolo, O. Doumbo, M. Daou, D. de Jong, L. A. B. Joosten, R. A. Tissingh, F. A. G. Reubsaet, R. Sauerwein, J. W. M. van der Meer, A. J. A. M. van der Ven, M. G. Netea, Caspase-12 and the inflammatory response to Yersinia pestis. *PLOS ONE* **4**, e6870 (2009).

48. R. M. Clay, *The Mediaeval Hospitals of England* (Methuen, 1909).

49. P. Richards, *The Medieval Leper and His Northern Heirs* (D.S. Brewer Ltd, 1977).

50. A. R. Barton, C. G. Santander, P. Skoglund, I. Moltke, D. Reich, I. Mathieson, Insufficient evidence for natural selection associated with the Black Death, Genomics, 2023); https://doi.org/10.1101/2023.03.14.532615.

51. P. G. Bronson, S. J. Mack, H. A. Erlich, M. Slatkin, A sequence-based approach demonstrates that balancing selection in classical human leukocyte antigen (HLA) loci is asymmetric. *Hum. Mol. Genet.* **22**, 252–261 (2013).

52. A. S. Maróstica, K. Nunes, E. C. Castelli, N. S. B. Silva, B. S. Weir, J. Goudet, D. Meyer, How HLA diversity is apportioned: influence of selection and relevance to transplantation. *Philos. Trans. R. Soc. B Biol. Sci.* **377**, 20200420 (2022).

53. D. Enard, D. A. Petrov, Evidence that RNA viruses drove adaptive introgression between Neanderthals and modern humans. *Cell* **175**, 360–371.e13 (2018).

54. E. Dunbar, G. T. Cook, P. Naysmith, B. G. Tripney, S. Xu, AMS ^{14}C dating at the Scottish Universities environmental research centre (SUERC) radiocarbon dating laboratory. *Radiocarbon* **58**, 9–23 (2016).

55. C. Bronk Ramsey, Bayesian analysis of radiocarbon dates. *Radiocarbon* **51**, 337–360 (2009).

56. C. Bronk Ramsey, S. Lee, Recent and planned developments of the program OxCal. *Radiocarbon* **55**, 720–730 (2013).

57. P. J. Reimer, E. Bard, A. Bayliss, J. W. Beck, P. G. Blackwell, C. B. Ramsey, C. E. Buck, H. Cheng, R. L. Edwards, M. Friedrich, P. M. Grootes, T. P. Guilderson, H. Haflidason, I. Hajdas, C. Hatté, T. J. Heaton, D. L. Hoffmann, A. G. Hogg, K. A. Hughen, K. F. Kaiser, B. Kromer, S. W. Manning, M. Niu, R. W. Reimer, D. A. Richards, E. M. Scott, J. R. Southon, R. A. Staff, C. S. M. Turney, J. van der Plicht, IntCal13 and marine13 radiocarbon age calibration curves 0–50,000 years cal BP. *Radiocarbon* **55**, 1869–1887 (2013).

58. P. J. Reimer, W. E. N. Austin, E. Bard, A. Bayliss, P. G. Blackwell, C. Bronk Ramsey, M. Butzin, H. Cheng, R. L. Edwards, M. Friedrich, P. M. Grootes, T. P. Guilderson, I. Hajdas, T. J. Heaton, A. G. Hogg, K. A. Hughen, B. Kromer, S. W. Manning, R. Muscheler, J. G. Palmer, C. Pearson, J. van der Plicht, R. W. Reimer, D. A. Richards, E. M. Scott, J. R. Southon, C. S. M. Turney, L. Wacker, F. Adolphi, U. Büntgen, M. Capano, S. M. Fahrni, A. Fogtmann-Schulz, R. Friedrich, P. Köhler, S. Kudsk, F. Miyake, J. Olsen, F. Reinig, M. Sakamoto, A. Sookdeo, S. Talamo, The IntCal20 Northern hemisphere radiocarbon age calibration Curve (0–55 cal kBP). *Radiocarbon* **62**, 725–757 (2020).

59. M. Meyer, M. Kircher, Illumina sequencing library preparation for highly multiplexed target capture and sequencing. *Cold Spring Harb. Protoc.* (2010), **2010** pdb.prot5448.

60. M. Martin, Cutadapt removes adapter sequences from high-throughput sequencing reads. *EMBnet.journal* **17**, 10 (2011).

61. H. Li, R. Durbin, Fast and accurate short read alignment with Burrows–Wheeler transform. *Bioinformatics* **25**, 1754–1760 (2009).

62. H. Li, B. Handsaker, A. Wysoker, T. Fennell, J. Ruan, N. Homer, G. Marth, G. Abecasis, R. Durbin, 1000 Genome Project Data Processing Subgroup, The sequence alignment/map format and SAMtools. *Bioinformatics* **25**, 2078–2079 (2009).

63. K. Okonechnikov, A. Conesa, F. García-Alcalde, Qualimap 2: Advanced multi-sample quality control for high-throughput sequencing data. *Bioinformatics* **32**, 292–294 (2016).

64. T. Derrien, J. Estellé, S. Marco Sola, D. G. Knowles, E. Raineri, R. Guigó, P. Ribeca, Fast computation and applications of genome mappability. *PLOS ONE* **7**, e30377 (2012).

65. H. Jónsson, A. Ginolhac, M. Schubert, P. L. F. Johnson, L. Orlando, mapDamage2.0: Fast approximate Bayesian estimates of ancient DNA damage parameters. *Bioinforma. Oxf. Engl.* **29**, 1682–1684 (2013).

66. Q. Fu, A. Mittnik, P. L. F. Johnson, K. Bos, M. Lari, R. Bollongino, C. Sun, L. Giemsch, R. Schmitz, J. Burger, A. M. Ronchitelli, F. Martini, R. G. Cremonesi, J. Svoboda, P. Bauer, D. Caramelli, S. Castellano, D. Reich, S. Pääbo, J. Krause, A revised timescale for human evolution based on ancient mitochondrial genomes. *Curr. Biol.* **23**, 553–559 (2013).

67. T. S. Korneliussen, A. Albrechtsen, R. Nielsen, ANGSD: Analysis of next generation sequencing data. *BMC Bioinformatics* **15**, 356 (2014).

68. P. Skoglund, J. Storå, A. Götherström, M. Jakobsson, Accurate sex identification of ancient human remains using DNA shotgun sequencing. *J. Archaeol. Sci.* **40**, 4477–4482 (2013).

69. H. Weissensteiner, D. Pacher, A. Kloss-Brandstätter, L. Forer, G. Specht, H.-J. Bandelt, F. Kronenberg, A. Salas, S. Schönherr, HaploGrep 2: Mitochondrial haplogroup classification in the era of high-throughput sequencing. *Nucleic Acids Res.* **44**, W58–W63 (2016).

70. P. Hallast, C. Batini, D. Zadik, P. Maisano Delser, J. H. Wetton, E. Arroyo-Pardo, G. L. Cavalleri, P. de Knijff, G. Destro Bisol, B. M. Dupuy, H. A. Eriksen, L. B. Jorde, T. E. King, M. H. Larmuseau, A. Lopez de Munain, A. M. Lopez-Parra, A. Loutradis, J. Milasin, A. Novelletto, H. Pamjav, A. Sajantila, W. Schempp, M. Sears, A. Tolun, C. Tyler-Smith, A. Van Geystelen, S. Watkins, B. Winney, M. A. Jobling, The Y-chromosome tree bursts into leaf: 13,000 high-confidence SNPs covering the majority of known clades. *Mol. Biol. Evol.* **32**, 661–673 (2015).

71. M. Karmin, L. Saag, M. Vicente, M. A. Wilson Sayres, M. Järve, U. G. Talas, S. Rootsi, A. M. Ilumäe, R. Mägi, M. Mitt, L. Pagani, T. Puurand, Z. Faltyskova, F. Clemente, A. Cardona, E.

Metspalu, H. Sahakyan, B. Yunusbayev, G. Hudjashov, M. DeGiorgio, E. L. Loogväli, C. Eichstaedt, M. Eelmets, G. Chaubey, K. Tambets, S. Litvinov, M. Mormina, Y. Xue, Q. Ayub, G. Zoraqi, T. S. Korneliussen, F. Akhatova, J. Lachance, S. Tishkoff, K. Momynaliev, F. X. Ricaut, P. Kusuma, H. Razafindrazaka, D. Pierron, M. P. Cox, G. N. N. Sultana, R. Willerslev, C. Muller, M. Westaway, D. Lambert, V. Skaro, L. Kovačević, S. Turdikulova, D. Dalimova, R. Khusainova, N. Trofimova, V. Akhmetova, I. Khidiyatova, D. V. Lichman, J. Isakova, E. Pocheshkhova, Z. Sabitov, N. A. Barashkov, P. Nymadawa, E. Mihailov, J. W. T. Seng, I. Evseeva, A. B. Migliano, S. Abdullah, G. Andriadze, D. Primorac, L. Atramentova, O. Utevska, L. Yepiskoposyan, D. Marjanović, A. Kushniarevich, D. M. Behar, C. Gilissen, L. Vissers, J. A. Veltman, E. Balanovska, M. Derenko, B. Malyarchuk, A. Metspalu, S. Fedorova, A. Eriksson, A. Manica, F. L. Mendez, T. M. Karafet, K. R. Veeramah, N. Bradman, M. F. Hammer, L. P. Osipova, O. Balanovsky, E. K. Khusnutdinova, K. Johnsen, M. Remm, M. G. Thomas, C. Tyler-Smith, P. A. Underhill, E. Willerslev, R. Nielsen, M. Metspalu, R. Villems, T. Kivisild, A recent bottleneck of Y chromosome diversity coincides with a global change in culture. *Genome Res.* **25**, 459–466 (2015).

72. G. D. Poznik, Y. Xue, F. L. Mendez, T. F. Willems, A. Massaia, M. A. Wilson Sayres, Q. Ayub, S. A. McCarthy, A. Narechania, S. Kashin, Y. Chen, R. Banerjee, J. L. Rodriguez-Flores, M. Cerezo, H. Shao, M. Gymrek, A. Malhotra, S. Louzada, R. Desalle, G. R. S. Ritchie, E. Cerveira, T. W. Fitzgerald, E. Garrison, A. Marcketta, D. Mittelman, M. Romanovitch, C. Zhang, X. Zheng-Bradley, G. R. Abecasis, S. A. McCarroll, P. Flicek, P. A. Underhill, L. Coin, D. R. Zerbino, F. Yang, C. Lee, L. Clarke, A. Auton, Y. Erlich, R. E. Handsaker, C. D. Bustamante, C. Tyler-Smith, Punctuated bursts in human male demography inferred from 1,244 worldwide Y-chromosome sequences. *Nat. Genet.* **48**, 593–599 (2016).

73. R. Martiniano, B. De Sanctis, P. Hallast, R. Durbin, Placing ancient DNA sequences into reference phylogenies. *Mol. Biol. Evol.* **39**, msac017 (2022).

74. R. Hui, E. D’Atanasio, L. M. Cassidy, C. L. Scheib, T. Kivisild, Evaluating genotype imputation pipeline for ultra-low coverage ancient genomes. *Sci. Rep.* **10**, 18542 (2020).

75. B. L. Browning, S. R. Browning, Genotype imputation with millions of reference samples. *Am. J. Hum. Genet.* **98**, 116–126 (2016).

76. B. L. Browning, Y. Zhou, S. R. Browning, A one-penny imputed genome from next-generation reference panels. *Am. J. Hum. Genet.* **103**, 338–348 (2018).

77. 1000 Genomes Project Consortium, A. Auton, L. D. Brooks, R. M. Durbin, E. P. Garrison, H. M. Kang, J. O. Korbel, J. L. Marchini, S. M. Carthy, G. A. Mc Vean, G. R. Abecasis, A global reference for human genetic variation. *Nature* **526**, 68–74 (2015).

78. M. Guellil, M. Keller, J. M. Dittmar, S. A. Inskip, C. Cessford, A. Solnik, T. Kivisild, M. Metspalu, J. E. Robb, C. L. Scheib, An invasive Haemophilus influenzae serotype b infection in an Anglo-Saxon plague victim. *Genome Biol.* **23**, 22 (2022).

79. A. McKenna, M. Hanna, E. Banks, A. Sivachenko, K. Cibulskis, A. Kernytsky, K. Garimella, D. Altshuler, S. Gabriel, M. Daly, M. A. DePristo, The genome analysis toolkit: A MapReduce framework for analyzing next-generation DNA sequencing data. *Genome Res.* **20**, 1297–1303 (2010).

80. G. Abraham, Y. Qiu, M. Inouye, FlashPCA2: principal component analysis of Biobank-scale genotype datasets. *Bioinformatics* **33**, 2776–2778 (2017).

81. Free Software Foundation, GNU Datamash, (2014); www.gnu.org/software/datamash/.

82. V. D. Blondel, J.-L. Guillaume, R. Lambiotte, E. Lefebvre, Fast unfolding of communities in large networks. *J. Stat. Mech. Theory Exp.*, **2008** P10008 (2008).

83. G. Csardi, T. Nepusz, The igraph software package for complex network research. *InterJournal Complex Syst.* 1695, 1–9 (2006).

84. C. C. Chang, C. C. Chow, L. C. Tellier, S. Vattikuti, S. M. Purcell, J. J. Lee, Second-generation PLINK: Rising to the challenge of larger and richer datasets. *GigaScience* **4**, 7 (2015).

85. P. Danecek, A. Auton, G. Abecasis, C. A. Albers, E. Banks, M. A. DePristo, R. E. Handsaker, G. Lunter, G. T. Marth, S. T. Sherry, G. McVean, R. Durbin, 1000 Genomes Project Analysis Group, The variant call format and VCFtools. *Bioinformatics* **27**, 2156–2158 (2011).

86. M. E. Allentoft, M. Sikora, K. G. Sjögren, S. Rasmussen, M. Rasmussen, J. Stenderup, P. B. Damgaard, H. Schroeder, T. Ahlström, L. Vinner, A. S. Malaspinas, A. Margaryan, T. Higham, D. Chivall, N. Lynnerup, L. Harvig, J. Baron, P. D. Casa, P. Dąbrowski, P. R. Duffy, A. V. Ebel, A. Epimakhov, K. Frei, M. Furmanek, T. Gralak, A. Gromov, S. Gronkiewicz, G. Grupe, T. Hajdu, R. Jarysz, V. Khartanovich, A. Khokhlov, V. Kiss, J. Kolář, A. Kriiska, I. Lasak, C. Longhi, G. McGlynn, A. Merkevicius, I. Merkyte, M. Metspalu, R. Mkrtchyan, V. Moiseyev, L. Paja, G. Pálfi, D. Pokutta, Ł. Pospieszny, T. Douglas Price, L. Saag, M. Sablin, N. Shishlina, V. Smrčka, V. I. Soenov, V. Szeverényi, G. Tóth, S. V. Trifanova, L. Varul, M. Vicze, L. Yepiskoposyan, V. Zhitenev, L. Orlando, T. Sicheritz-Pontén, S. Brunak, R. Nielsen, K. Kristiansen, E. Willerslev, Population genomics of Bronze Age Eurasia. *Nature* **522**, 167–172 (2015).

87. T. Günther, H. Malmström, E. M. Svensson, A. Omrak, F. Sánchez-Quinto, G. M. Kılınç, M. Krzewińska, G. Eriksson, M. Fraser, H. Edlund, A. R. Munters, A. Coutinho, L. G. Simões, M. Vicente, A. Sjölander, B. Jansen Sellevold, R. Jørgensen, P. Claes, M. D. Shriver, C. Valdiosera, M. G. Netea, J. Apel, K. Lidén, B. Skar, J. Storå, A. Götherström, M. Jakobsson, Population genomics of Mesolithic Scandinavia: Investigating early postglacial migration routes and high-latitude adaptation. *PLoS Biol.* **16**, e2003703 (2018).

88. I. Olalde, M. E. Allentoft, F. Sánchez-Quinto, G. Santpere, C. W. K. Chiang, M. DeGiorgio, J. Prado-Martinez, J. A. Rodríguez, S. Rasmussen, J. Quilez, O. Ramírez, U. M. Marigorta, M. Fernández-Callejo, M. E. Prada, J. M. V. Encinas, R. Nielsen, M. G. Netea, J. Novembre, R. A. Sturm, P. Sabeti, T. Marquès-Bonet, A. Navarro, E. Willerslev, C. Lalueza-Fox, Derived immune and ancestral pigmentation alleles in a 7,000-year-old Mesolithic European. *Nature* **507**, 225–228 (2014).

89. L. Saag, S. V. Vasilyev, L. Varul, N. V. Kosorukova, D. V. Gerasimov, S. V. Oshibkina, S. J. Griffith, A. Solnik, L. Saag, E. D'Atanasio, E. Metspalu, M. Reidla, S. Rootsi, T. Kivisild, C. L. Scheib, K. Tambets, A. Kriiska, M. Metspalu, Genetic ancestry changes in stone to bronze age transition in the East European plain. *Sci. Adv.* **7**, eabd6535 (2021).

90. T. Saupe, F. Montinaro, C. Scaggion, N. Carrara, T. Kivisild, E. D'Atanasio, R. Hui, A. Solnik, O. Lebrasseur, G. Larson, L. Alessandri, I. Arienzo, F. De Angelis, M. F. Rolfo, R. Skeates, L.

Silvestri, J. Beckett, S. Talamo, A. Dolfini, M. Miari, M. Metspalu, S. Benazzi, C. Capelli, L. Pagani, C. L. Scheib, Ancient genomes reveal structural shifts after the arrival of Steppe-related ancestry in the Italian Peninsula. *Curr. Biol.* **31**, 2576–2591.e12 (2021).

91. B. S. Weir, C. C. Cockerham, Estimating F-statistics for the analysis of population structure. *Evolution* **38**, 1358–1370 (1984).

92. C. Cessford, A. Dickens, The Manor of Hintona: The origins and development of Church End, Cherry Hinton. *Proc. Camb. Antiqu. Soc.* **94**, 51–72 (2005).

93. C. Cessford, A. Slater, Beyond the manor of hintona further thoughts on the development of church end, cherry hinton: The neath farm site. *Proc. Camb. Antiqu. Soc.* **103**, 39–60 (2014).

94. M. Lally, 69 to 115 Church End, Cherry Hinton, Cambridgeshire: Post Excavation Assessment and Updated Project Design (Archaeological Solutions Report 3012, 2008).

95. HMC, Sixth Report of the Royal Commission on Historical Manuscripts. Part I: Report and Appendix (HMSO, 1877).

96. C. Cessford, A. Dickens, Castle Hill, Cambridge: Excavation of Saxon, medieval and post-medieval deposits, Saxon execution site and a medieval coinhoard. *Proc. Camb. Antiqu. Soc.* **94**, 73–102 (2005).

97. M. Rubin, *Charity and Community in Medieval Cambridge* (Cambridge Univ. Press, ed. 1, 1987.

98. M. Underwood, Ed., *The Cartulary of the Hospital of St John the Evangelist, Cambridge* (Cambridgeshire Records Society Map Series, Cambridgeshire Records Society, 2008).

99. C. Cessford, The St. John's hospital cemetery and environs, Cambridge: Contextualizing the medieval urban dead: Contextualizing the medieval urban dead. *Archaeol. J.* **172**, 52–120 (2015).

100. C. Cessford, Former Old Examination Hall, North Range Buildings, New Museums Site, Cambridge: an archaeological excavation (Cambridge Archaeology Unit, 2017).

101. C. Cessford, North Range Buildings, New Museums Site, Cambridge: further archaeological investigations (Cambridge Archaeology Unit, 2020).

102. C. Cessford, B. Neil, The people of the Cambridge Austin friars. *Archaeol. J.* **179**, 383–444 (2022).

103. C. Cessford, M. Samuel, V. Herring, N. Holder, P. Mills, The architecture of the Augustinian Friary, Cambridge, *Antiqu. J.*, **103** 162–194 (2023).

104. C. Cessford, A. Hall, B. Mulder, B. Neil, I. Riddler, J. Wiles, E. Cameron, Q. Mould, Buried with their buckles on: Clothed burial at the Augustinian Friary Cambridge, *Mediev. Archaeol.* **66**, 151–187 (2022).

105. C. Cessford, D. Fallon, Hostel Yard and Environs, Corpus Christi College, Cambridge: An Archaeological Watching Brief" (2006).

106. G. Rees, A 19th Century Baptist Cemetery at St Matthew's Primary School, Norfolk Street, Cambridge (Oxford Archaeology East, 2014).

107. R. Newman, Holy Trinity Church, Cambridge: Archaeological Excavation and Monitoring, 2016–2017 (Archaeology Data Service, 2018).

108. J. A. Alexander, "Clopton: the life-cycle of a Cambridgeshire village" in *East Anglian Studies*, L. M. Munby, Ed. (Heffer and Sons, 1968), pp. 48–70.

109. S. Sims, Hemingfords Flood Alleviation Scheme, St Ives, Cambridgeshire: archaeological watching brief report (Oxford Archaeology, 2007).

110. C. Fowler, I. Olalde, V. Cummings, I. Armit, L. Büster, S. Cuthbert, N. Rohland, O. Cheronet, R. Pinhasi, D. Reich, A high-resolution picture of kinship practices in an Early Neolithic tomb. *Nature* **601**, 584–587 (2022).

111. I. Olalde, S. Brace, M. E. Allentoft, I. Armit, K. Kristiansen, T. Booth, N. Rohland, S. Mallick, A. Szécsényi-Nagy, A. Mittnik, E. Altena, M. Lipson, I. Lazaridis, T. K. Harper, N. Patterson, N. Broomandkhoshbacht, Y. Diekmann, Z. Faltyskova, D. Fernandes, M. Ferry, E. Harney, P. De

Knijff, M. Michel, J. Oppenheimer, K. Stewardson, A. Barclay, K. W. Alt, C. Liesau, P. Rios, C. Blasco, J. V. Miguel, R. M. Garcia, A. A. Fernandez, E. Banffy, M. Bernabo-Brea, D. Billoin, C. Bonsall, L. Bonsall, T. Allen, L. Buster, S. Carver, L. C. Navarro, O. E. Craig, G. T. Cook, B. Cunliffe, A. Denaire, K. E. Dinwiddy, N. Dodwell, M. Ernee, C. Evans, M. Kucharik, J. F. Farre, C. Fowler, M. Gazenbeek, R. G. Pena, M. Haber-Uriarte, E. Haduch, G. Hey, N. Jowett, T. Knowles, K. Massy, S. Pfrengle, P. Lefranc, O. Lemercier, A. Lefebvre, C. H. Martinez, V. G. Olmo, A. B. Ramirez, J. L. Maurandi, T. Majo, J. I. McKinley, K. McSweeney, B. G. Mende, A. Mod, G. Kulcsar, V. Kiss, A. Czene, R. Patay, A. Endrodi, K. Kohler, T. Hajdu, T. Szeniczey, J. Dani, Z. Bernert, M. Hoole, O. Cheronet, D. Keating, P. Veleminsky, M. Dobe, F. Candilio, F. Brown, R. F. Fernandez, A. M. Herrero-Corral, S. Tusa, E. Carnieri, L. Lentini, A. Valenti, A. Zanini, C. Waddington, G. Delibes, E. Guerra-Doce, B. Neil, M. Brittain, M. Luke, R. Mortimer, J. Desideri, M. Besse, G. Brucken, M. Furmanek, A. Hauszko, M. Mackiewicz, A. Rapinski, S. Leach, I. Soriano, K. T. Lillios, J. L. Cardoso, M. P. Pearson, P. Wodarczak, T. D. Price, P. Prieto, P. J. Rey, R. Risch, M. A. R. Guerra, A. Schmitt, J. Serralongue, A. M. Silva, V. Smrcka, L. Vergnaud, J. Zilhao, D. Caramelli, T. Higham, M. G. Thomas, D. J. Kennett, H. Fokkens, V. Heyd, A. Sheridan, K. G. Sjogren, P. W. Stockhammer, J. Krause, R. Pinhasi, W. Haak, I. Barnes, C. Lalueza-Fox, D. Reich, The Beaker phenomenon and the genomic transformation of northwest Europe. *Nature* **555**, 190–196 (2018).

112. N. Patterson, M. Isakov, T. Booth, L. Büster, C.-E. Fischer, I. Olalde, H. Ringbauer, A. Akbari, O. Cheronet, M. Bleasdale, N. Adamski, E. Altena, R. Bernardos, S. Brace, N. Broomandkhoshbacht, K. Callan, F. Candilio, B. Culleton, E. Curtis, L. Demetz, K. S. D. Carlson, C. J. Edwards, D. M. Fernandes, M. G. B. Foody, S. Freilich, H. Goodchild, A. Kearns, A. M. Lawson, I. Lazaridis, M. Mah, S. Mallick, K. Mandl, A. Micco, M. Michel, G. B. Morante, J. Oppenheimer, K. T. Özdoğan, L. Qiu, C. Schattke, K. Stewardson, J. N. Workman, F. Zalzala, Z. Zhang, B. Agustí, T. Allen, K. Almássy, L. Amkreutz, A. Ash, C. Baillif-Ducros, A. Barclay, L. Bartosiewicz, K. Baxter, Z. Bernert, J. Blažek, M. Bodružić, P. Boissinot, C. Bonsall, P. Bradley, M. Brittain, A. Brookes, F. Brown, L. Brown, R. Brunning, C. Budd, J. Burmaz, S. Canet, S. Carnicero-Cáceres, M. Čaušević-Bully, A. Chamberlain, S. Chauvin, S. Clough, N. Čondić, A. Coppa, O. Craig, M. Črešnar, V. Cummings, S. Czifra, A. Danielisová, R. Daniels, A. Davies, P. De Jersey, J. Deacon, C. Deminger, P. W. Ditchfield, M. Dizdar, M. Dobeš, M. Dobisíková, L. Domboróczki, G. Drinkall, A. Đukić, M.

Ernée, C. Evans, J. Evans, M. Fernández-Götz, S. Filipović, A. Fitzpatrick, H. Fokkens, C. Fowler, A. Fox, Z. Gallina, M. Gamble, M. R. González Morales, B. González-Rabanal, A. Green, K. Gyenesei, D. Habermehl, T. Hajdu, D. Hamilton, J. Harris, C. Hayden, J. Hendriks, B. Hernu, G. Hey, M. Horňák, G. Ilon, E. Istvánovits, A. M. Jones, M. B. Kavur, K. Kazek, R. A. Kenyon, A. Khreisheh, V. Kiss, J. Kleijne, M. Knight, L. M. Kootker, P. F. Kovács, A. Kozubová, G. Kulcsár, V. Kulcsár, C. Le Pennec, M. Legge, M. Leivers, L. Loe, O. López-Costas, T. Lord, D. Los, J. Lyall, A. B. Marín-Arroyo, P. Mason, D. Matošević, A. Maxted, L. McIntyre, J. McKinley, K. McSweeney, B. Meijlink, B. G. Mende, M. Menđušić, M. Metlička, S. Meyer, K. Mihovilić, L. Milasinovic, S. Minnitt, J. Moore, G. Morley, G. Mullan, M. Musilová, B. Neil, R. Nicholls, M. Novak, M. Pala, M. Papworth, C. Paresys, R. Patten, D. Perkić, K. Pesti, A. Petit, K. Petriščáková, C. Pichon, C. Pickard, Z. Pilling, T. D. Price, S. Radović, R. Redfern, B. Resutík, D. T. Rhodes, M. B. Richards, A. Roberts, J. Roefstra, P. Sankot, A. Šefčáková, A. Sheridan, S. Skae, M. Šmolíková, K. Somogyi, Á. Somogyvári, M. Stephens, G. Szabó, A. Szécsényi-Nagy, T. Szeniczey, J. Tabor, K. Tankó, C. T. Maria, R. Terry, B. Teržan, M. Teschler-Nicola, J. F. Torres-Martínez, J. Trapp, R. Turle, F. Ujvári, M. Van Der Heiden, P. Veleminsky, B. Veselka, Z. Vytlačil, C. Waddington, P. Ware, P. Wilkinson, L. Wilson, R. Wiseman, E. Young, J. Zaninović, A. Žitňan, C. Lalueza-Fox, P. De Knijff, I. Barnes, P. Halkon, M. G. Thomas, D. J. Kennett, B. Cunliffe, M. Lillie, N. Rohland, R. Pinhasi, I. Armit, D. Reich, Large-scale migration into Britain during the middle to late bronze age. *Nature* **601**, 588–594 (2022).

113. S. Brace, Y. Diekmann, T. J. Booth, L. van Dorp, Z. Faltyskova, N. Rohland, S. Mallick, I. Olalde, M. Ferry, M. Michel, J. Oppenheimer, N. Broomandkhoshbacht, K. Stewardson, R. Martiniano, S. Walsh, M. Kayser, S. Charlton, G. Hellenthal, I. Armit, R. Schulting, O. E. Craig, A. Sheridan, M. Parker Pearson, C. Stringer, D. Reich, M. G. Thomas, I. Barnes, Ancient genomes indicate population replacement in Early Neolithic Britain. *Nat. Ecol. Evol.* **3**, 765–771 (2019).

114. C. L. Scheib, R. Hui, E. D'Atanasio, A. W. Wohns, S. A. Inskip, A. Rose, C. Cessford, T. C. O'Connell, J. E. Robb, C. Evans, R. Patten, T. Kivisild, East Anglian early Neolithic monument burial linked to contemporary Megaliths. *Ann. Hum. Biol.* **46**, 145–149 (2019).

115. N. S. Enattah, T. Sahi, E. Savilahti, J. D. Terwilliger, L. Peltonen, I. Järvelä, Identification of a variant associated with adult-type hypolactasia. *Nat. Genet.* **30**, 233–237 (2002).

116. A. M. Hancock, D. B. Witonsky, E. Ehler, G. Alkorta-Aranburu, C. Beall, A. Gebremedhin, R. Sukernik, G. Utermann, J. Pritchard, G. Coop, A. Di Rienzo, Colloquium paper: Human adaptations to diet, subsistence, and ecoregion are due to subtle shifts in allele frequency. *Proc. Natl. Acad. Sci. U. S. A.* **107 Suppl 2**, 8924–8930 (2010).

117. Y. S. Aulchenko, S. Ripatti, I. Lindqvist, D. Boomsma, I. M. Heid, P. P. Pramstaller, B. W. J. H. Penninx, A. C. J. W. Janssens, J. F. Wilson, T. Spector, N. G. Martin, N. L. Pedersen, K. O. Kyvik, J. Kaprio, A. Hofman, N. B. Freimer, M.-R. Jarvelin, U. Gyllensten, H. Campbell, I. Rudan, A. Johansson, F. Marroni, C. Hayward, V. Vitart, I. Jonasson, C. Pattaro, A. Wright, N. Hastie, I. Pichler, A. A. Hicks, M. Falchi, G. Willemsen, J.-J. Hottenga, E. J. C. de Geus, G. W. Montgomery, J. Whitfield, P. Magnusson, J. Saharinen, M. Perola, K. Silander, A. Isaacs, E. J. G. Sijbrands, A. G. Uitterlinden, J. C. M. Witteman, B. A. Oostra, P. Elliott, A. Ruokonen, C. Sabatti, C. Gieger, T. Meitinger, F. Kronenberg, A. Döring, H.-E. Wichmann, J. H. Smit, M. I. McCarthy, C. M. van Duijn, L. Peltonen, Loci influencing lipid levels and coronary heart disease risk in 16 European population cohorts. *Nat. Genet.* **41**, 47–55 (2009).

118. F. Racimo, D. Marnetto, E. Huerta-Sánchez, Signatures of Archaic adaptive introgression in present-day human populations. *Mol. Biol. Evol.* **34**, 296–317 (2017).

119. C. Sabatti, S. K. Service, A.-L. Hartikainen, A. Pouta, S. Ripatti, J. Brodsky, C. G. Jones, N. A. Zaitlen, T. Varilo, M. Kaakinen, U. Sovio, A. Ruokonen, J. Laitinen, E. Jakkula, L. Coin, C. Hoggart, A. Collins, H. Turunen, S. Gabriel, P. Elliot, M. I. McCarthy, M. J. Daly, M.-R. Järvelin, N. B. Freimer, L. Peltonen, Genome-wide association analysis of metabolic traits in a birth cohort from a founder population. *Nat. Genet.* **41**, 35–46 (2009).

120. S. Kathiresan, O. Melander, C. Guiducci, A. Surti, N. P. Burtt, M. J. Rieder, G. M. Cooper, C. Roos, B. F. Voight, A. S. Havulinna, B. Wahlstrand, T. Hedner, D. Corella, E. S. Tai, J. M. Ordovas, G. Berglund, E. Vartiainen, P. Jousilahti, B. Hedblad, M.-R. Taskinen, C. Newton-Cheh, V. Salomaa, L. Peltonen, L. Groop, D. M. Altshuler, M. Orho-Melander, Six new loci associated with blood low-density lipoprotein cholesterol, high-density lipoprotein cholesterol or triglycerides in humans. *Nat. Genet.* **40**, 189–197 (2008).

121. M. García-Closas, D. W. Hein, D. Silverman, N. Malats, M. Yeager, K. Jacobs, M. A. Doll, J. D. Figueroa, D. Baris, M. Schwenn, M. Kogevinas, A. Johnson, N. Chatterjee, L. E. Moore, T. Moeller, F. X. Real, S. Chanock, N. Rothman, A single nucleotide polymorphism tags variation in the arylamine N-acetyltransferase 2 phenotype in populations of European background. *Pharmacogenet. Genomics* **21**, 231–236 (2011).

122. A. Sabbagh, P. Darlu, B. Crouau-Roy, E. S. Poloni, Arylamine N-acetyltransferase 2 (NAT2) genetic diversity and traditional subsistence: A worldwide population survey. *PLOS ONE* **6**, e18507 (2011).

123. S. Mathieson, I. Mathieson, FADS1 and the timing of human adaptation to agriculture. *Mol. Biol. Evol.* **35**, 2957–2970 (2018).

124. T. J. Wang, F. Zhang, J. B. Richards, B. Kestenbaum, J. B. van Meurs, D. Berry, D. P. Kiel, E. A. Streeten, C. Ohlsson, D. L. Koller, L. Peltonen, J. D. Cooper, P. F. O'Reilly, D. K. Houston, N. L. Glazer, L. Vandenput, M. Peacock, J. Shi, F. Rivadeneira, M. I. McCarthy, P. Anneli, I. H. de Boer, M. Mangino, B. Kato, D. J. Smyth, S. L. Booth, P. F. Jacques, G. L. Burke, M. Goodarzi, C.-L. Cheung, M. Wolf, K. Rice, D. Goltzman, N. Hidiroglou, M. Ladouceur, N. J. Wareham, L. J. Hocking, D. Hart, N. K. Arden, C. Cooper, S. Malik, W. D. Fraser, A.-L. Hartikainen, G. Zhai, H. M. Macdonald, N. G. Forouhi, R. J. F. Loos, D. M. Reid, A. Hakim, E. Dennison, Y. Liu, C. Power, H. E. Stevens, L. Jaana, R. S. Vasan, N. Soranzo, J. Bojunga, B. M. Psaty, M. Lorentzon, T. Foroud, T. B. Harris, A. Hofman, J.-O. Jansson, J. A. Cauley, A. G. Uitterlinden, Q. Gibson, M.-R. Järvelin, D. Karasik, D. S. Siscovick, M. J. Econs, S. B. Kritchevsky, J. C. Florez, J. A. Todd, J. Dupuis, E. Hyppönen, T. D. Spector, Common genetic determinants of vitamin D insufficiency: a genome-wide association study. *Lancet* **376**, 180–188 (2010).

125. H. D. Shin, C. Winkler, J. C. Stephens, J. Bream, H. Young, J. J. Goedert, T. R. O'Brien, D. Vlahov, S. Buchbinder, J. Giorgi, C. Rinaldo, S. Donfield, A. Willoughby, S. J. O'Brien, M. W. Smith, Genetic restriction of HIV-1 pathogenesis to AIDS by promoter alleles of IL10. *Proc. Natl. Acad. Sci. U. S. A.* **97**, 14467–14472 (2000).

126. S. Shrestha, H. W. Wiener, B. Aissani, W. Song, A. Shendre, C. M. Wilson, R. A. Kaslow, J. Tang, Interleukin-10 (IL-10) pathway: genetic variants and outcomes of HIV-1 infection in African American adolescents. *PLOS ONE* **5**, e13384 (2010).

127. J. M. Valverde-Villegas, B. P. Dos Santos, R. M. de Medeiros, V. S. Mattevi, R. K. Lazzaretti, E. Sprinz, R. Kuhmmer, J. A. B. Chies, Endosomal toll-like receptor gene polymorphisms and susceptibility to HIV and HCV co-infection - Differential influence in individuals with distinct ethnic background. *Hum. Immunol.* **78**, 221–226 (2017).

128. M. Sironi, M. Biasin, R. Cagliani, D. Forni, M. De Luca, I. Saulle, S. Lo Caputo, F. Mazzotta, J. Macías, J. A. Pineda, A. Caruz, M. Clerici, A common polymorphism in TLR3 confers natural resistance to HIV-1 infection. *J. Immunol. Baltim. Md* **188**, 818–823 (2012).

129. M. P. Martin, M. M. Lederman, H. B. Hutcheson, J. J. Goedert, G. W. Nelson, Y. van Kooyk, R. Detels, S. Buchbinder, K. Hoots, D. Vlahov, S. J. O'Brien, M. Carrington, Association of DC-SIGN promoter polymorphism with increased risk for parenteral, but not mucosal, acquisition of human immunodeficiency virus type 1 infection. *J. Virol.* **78**, 14053–14056 (2004).

130. A. Al-Qahtani, M. Al-Ahdal, A. Abdo, F. Sanai, M. Al-Anazi, N. Khalaf, N. A. Viswan, H. Al-Ashgar, H. Al-Humaidan, R. Al-Suwayeh, Z. Hussain, S. Alarifi, M. Al-Okail, F. N. Almajhdi, Toll-like receptor 3 polymorphism and its association with hepatitis B virus infection in Saudi Arabian patients. *J. Med. Virol.* **84**, 1353–1359 (2012).

131. C. M. Johnson, E. A. Lyle, K. O. Omueti, V. A. Stepensky, O. Yegin, E. Alpsoy, L. Hamann, R. R. Schumann, R. I. Tapping, Cutting Edge: A common polymorphism impairs cell surface trafficking and functional responses of TLR1 but protects against leprosy. *J. Immunol.* **178**, 7520–7524 (2007).

132. S. H. Wong, S. Gochhait, D. Malhotra, F. H. Pettersson, Y. Y. Teo, C. C. Khor, A. Rautanen, S. J. Chapman, T. C. Mills, A. Srivastava, A. Rudko, M. B. Freidin, V. P. Puzyrev, S. Ali, S. Aggarwal, R. Chopra, B. S. N. Reddy, V. K. Garg, S. Roy, S. Meisner, S. K. Hazra, B. Saha, S. Floyd, B. J. Keating, C. Kim, B. P. Fairfax, J. C. Knight, P. C. Hill, R. A. Adegbola, H. Hakonarson, P. E. M. Fine, R. M. Pitchappan, R. N. K. Bamezai, A. V. S. Hill, F. O. Vannberg, Leprosy and the adaptation of human toll-like receptor 1. *PLOS Pathog.* **6**, e1000979 (2010).

133. R. P. Schuring, L. Hamann, W. R. Faber, D. Pahan, J. H. Richardus, R. R. Schumann, L. Oskam, Polymorphism N248S in the human Toll-like receptor 1 gene is related to leprosy and leprosy reactions. *J Infect Dis* **199**, 1816–1819 (2009).

134. B. R. Sapkota, M. Macdonald, W. R. Berrington, E. A. Misch, C. Ranjit, M. R. Siddiqui, G. Kaplan, T. R. Hawn, Association of TNF, MBL, and VDR polymorphisms with leprosy phenotypes. *Hum. Immunol.* **71**, 992–998 (2010).

135. G A V Silva, R. Ramasawmy, A L Boechat, A C Morais, B K S Carvalho, K B A Sousa, V C Souza, M G S Cunha, R H Barletta-Naveca, M P Santos, F G Naveca, Association of TNF -1031 C/C as a potential protection marker for leprosy development in Amazonas state patients, Brazil. *Hum. Immunol.* **76**, 137–141 (2015).

136. B. Krause-Kyora, J. Susat, F. M. Key, D. Kühnert, E. Bosse, A. Immel, C. Rinne, S.-C. Kornell, D. Yepes, S. Franzenburg, H. O. Heyne, T. Meier, S. Lösch, H. Meller, S. Friederich, N. Nicklisch, K. W. Alt, S. Schreiber, A. Tholey, A. Herbig, A. Nebel, J. Krause, Neolithic and medieval virus genomes reveal complex evolution of hepatitis B. *eLife* **7**, e36666 (2018).

137. E. A. Misch, W. R. Berrington, J. C. Vary Jr., T. R. Hawn, Leprosy and the human genome. *Microbiol. Mol. Biol. Rev.* **74**, 589–620 (2010).

138. V. M. Fava, C. Sales-Marques, A. Alcaïs, M. O. Moraes, E. Schurr, Age-dependent association of TNFSF15/TNFSF8 variants and leprosy type 1 reaction. *Front. Immunol.* **8**:155 (2017).

139. Y. Sun, A. Irwanto, L. Toyo-Oka, M. Hong, H. Liu, A. K. Andiappan, H. Choi, Y. Hitomi, G. Yu, Y. Yu, F. Bao, C. Wang, X. Fu, Z. Yue, H. Wang, H. Zhang, M. Kawashima, K. Kojima, M. Nagasaki, M. Nakamura, S.-K. Yang, B. D. Ye, Y. Denise, O. Rotzschke, K. Song, K. Tokunaga, F. Zhang, J. Liu, Fine-mapping analysis revealed complex pleiotropic effect and tissue-specific regulatory mechanism of TNFSF15 in primary biliary cholangitis Crohn’s disease and leprosy. *Sci. Rep.* **6**, 31429 (2016).

140. C. Sales-Marques, H. Salomão, V. M. Fava, L. E. Alvarado-Arnez, E. P. Amaral, C. C. Cardoso, I. M. F. Dias-Batista, W. L. da Silva, P. Medeiros, M. da Cunha Lopes Virmond, F. C. F. Lana, A. G.

Pacheco, M. O. Moraes, M. T. Mira, A. C. Pereira Latini, NOD2 and CCDC122-LACC1 genes are associated with leprosy susceptibility in Brazilians. *Hum. Genet.* **133**, 1525–1532 (2014).

141. H. Schurz, M. Daya, M. Möller, E. G. Hoal, M. Salie, TLR1, 2, 4, 6 and 9 variants associated with tuberculosis susceptibility: A systematic review and meta-analysis. *PLOS ONE* **10**, e0139711 (2015).

142. M. Dannemann, A. M. Andrés, J. Kelso, Introgression of Neandertal- and Denisovan-like haplotypes contributes to adaptive variation in human toll-like receptors. *Am. J. Hum. Genet.* **98**, 22–33 (2016).

143. E. Sánchez, J. M. Sabio, J. L. Callejas, E. de Ramón, R. Garcia-Portales, F. J. García-Hernández, J. Jiménez-Alonso, M. F. González-Escribano, J. Martín, B. P. Koeleman, Association study of genetic variants of pro-inflammatory chemokine and cytokine genes in systemic lupus erythematosus. *BMC Med. Genet.* **7**, 48 (2006).

144. J. A. Shah, J. C. Vary, T. T. H. Chau, N. D. Bang, N. T. B. Yen, J. J. Farrar, S. J. Dunstan, T. R. Hawn, Human TOLLIP regulates TLR2 and TLR4 signaling and its polymorphisms are associated with susceptibility to tuberculosis. *J. Immunol.* **189**, 1737–1746 (2012).

145. C. C. Khor, S. J. Chapman, F. O. Vannberg, A. Dunne, C. Murphy, E. Y. Ling, A. J. Frodsham, A. J. Walley, O. Kyrieleis, A. Khan, C. Aucan, S. Segal, C. E. Moore, K. Knox, S. J. Campbell, C. Lienhardt, A. Scott, P. Aaby, O. Y. Sow, R. T. Grignani, J. Sillah, G. Sirugo, N. Peshu, T. N. Williams, K. Maitland, R. J. O. Davies, D. P. Kwiatkowski, N. P. Day, D. Yala, D. W. Crook, K. Marsh, J. A. Berkley, L. A. J. O'Neill, A. V. S. Hill, A Mal functional variant is associated with protection against invasive pneumococcal disease, bacteremia, malaria and tuberculosis. *Nat. Genet.* **39**, 523–528 (2007).

146. P. O. Flores-Villanueva, J. A. Ruiz-Morales, C.-H. Song, L. M. Flores, E.-K. Jo, M. Montaño, P. F. Barnes, M. Selman, J. Granados, A functional promoter polymorphism in monocyte chemoattractant protein-1 is associated with increased susceptibility to pulmonary tuberculosis. *J. Exp. Med.* **202**, 1649–1658 (2005).

147. G. Kerner, G. Laval, E. Patin, S. Boisson-Dupuis, L. Abel, J.-L. Casanova, L. Quintana-Murci, Human ancient DNA analyses reveal the high burden of tuberculosis in Europeans over the last 2,000 years. *Am. J. Hum. Genet.* **108**, 517–524 (2021).

148. M. Saleh, J. P. Vaillancourt, R. K. Graham, M. Huyck, S. M. Srinivasula, E. S. Alnemri, M. H. Steinberg, V. Nolan, C. T. Baldwin, R. S. Hotchkiss, T. G. Buchman, B. A. Zehnbauer, M. R. Hayden, L. A. Farrer, S. Roy, D. W. Nicholson, Differential modulation of endotoxin responsiveness by human caspase-12 polymorphisms. *Nature* **429**, 75–79 (2004).

149. K. Fujikura, Multiple loss-of-function variants of taste receptors in modern humans. *Sci. Rep.* **5**, 12349 (2015).

150. A. M. Sutherland, K. R. Walley, T.-A. Nakada, A. H. P. Sham, M. M. Wurfel, J. A. Russell, A nonsynonymous polymorphism of IRAK4 associated with increased prevalence of gram-positive infection and decreased response to toll-like receptor ligands. *J. Innate Immun.* **3**, 447–458 (2011).

151. K. A. Hunt, A. Zhernakova, G. Turner, G. A. R. Heap, L. Franke, M. Bruinenberg, J. Romanos, L. C. Dinesen, A. W. Ryan, D. Panesar, R. Gwilliam, F. Takeuchi, W. M. McLaren, G. K. T. Holmes, P. D. Howdle, J. R. F. Walters, D. S. Sanders, R. J. Playford, G. Trynka, C. J. J. Mulder, M. L. Mearin, W. H. M. Verbeek, V. Trimble, F. M. Stevens, C. O'Morain, N. P. Kennedy, D. Kelleher, D. J. Pennington, D. P. Strachan, W. L. McArdle, C. A. Mein, M. C. Wapenaar, P. Deloukas, R. McGinnis, R. McManus, C. Wijmenga, D. A. van Heel, Newly identified genetic risk variants for celiac disease related to the immune response. *Nat. Genet.* **40**, 395–402 (2008).

152. A. Zhernakova, C. C. Elbers, B. Ferwerda, J. Romanos, G. Trynka, P. C. Dubois, C. G. F. de Kovel, L. Franke, M. Oosting, D. Barisani, M. T. Bardella, Finnish Celiac Disease Study Group, L. A. B. Joosten, P. Saavalainen, D. A. van Heel, C. Catassi, M. G. Netea, C. Wijmenga, Evolutionary and functional analysis of celiac risk loci reveals SH2B3 as a protective factor against bacterial infection. *Am. J. Hum. Genet.* **86**, 970–977 (2010).

153. A. J. Monsuur, P. I. W. de Bakker, A. Zhernakova, D. Pinto, W. Verduijn, J. Romanos, R. Auricchio, A. Lopez, D. A. van Heel, J. B. A. Crusius, C. Wijmenga, Effective detection of human

leukocyte antigen risk alleles in celiac disease using tag single nucleotide polymorphisms. *PLOS ONE* **3**, e2270 (2008).

154. E. A. Stahl, S. Raychaudhuri, E. F. Remmers, G. Xie, S. Eyre, B. P. Thomson, Y. Li, F. A. S. Kurreeman, A. Zhernakova, A. Hinks, C. Guiducci, R. Chen, L. Alfredsson, C. I. Amos, K. G. Ardlie, BIRAC Consortium, A. Barton, J. Bowes, E. Brouwer, N. P. Burtt, J. J. Catanese, J. Coblyn, M. J. H. Coenen, K. H. Costenbader, L. A. Criswell, J. B. A. Crusius, J. Cui, P. I. W. de Bakker, P. L. De Jager, B. Ding, P. Emery, E. Flynn, P. Harrison, L. J. Hocking, T. W. J. Huizinga, D. L. Kastner, X. Ke, A. T. Lee, X. Liu, P. Martin, A. W. Morgan, L. Padyukov, M. D. Posthumus, T. R. D. J. Radstake, D. M. Reid, M. Seielstad, M. F. Seldin, N. A. Shadick, S. Steer, P. P. Tak, W. Thomson, A. H. M. van der Helm-van Mil, I. E. van der Horst-Bruinsma, C. E. van der Schoot, P. L. C. M. van Riel, M. E. Weinblatt, A. G. Wilson, G. J. Wolbink, B. P. Wordsworth, YEAR Consortium, C. Wijmenga, E. W. Karlson, R. E. M. Toes, N. de Vries, A. B. Begovich, J. Worthington, K. A. Siminovitch, P. K. Gregersen, L. Klareskog, R. M. Plenge, Genome-wide association study meta-analysis identifies seven new rheumatoid arthritis risk loci. *Nat. Genet.* **42**, 508–514 (2010).

155. V. D. Peltekova, R. F. Wintle, L. A. Rubin, C. I. Amos, Q. Huang, X. Gu, B. Newman, M. Van Oene, D. Cescon, G. Greenberg, A. M. Griffiths, P. H. St George-Hyslop, K. A. Siminovitch, Functional variants of OCTN cation transporter genes are associated with Crohn disease. *Nat. Genet.* **36**, 471–475 (2004).

156. P. Gaj, A. Habior, M. Mikula, J. Ostrowski, Lack of evidence for association of primary sclerosing cholangitis and primary biliary cirrhosis with risk alleles for Crohn's disease in Polish patients. *BMC Med. Genet.* **9**, 81 (2008).

157. S. Nakagome, S. Mano, L. Kozlowski, J. M. Bujnicki, H. Shibata, Y. Fukumaki, J. R. Kidd, K. K. Kidd, S. Kawamura, H. Oota, Crohn's disease risk alleles on the NOD2 locus have been maintained by natural selection on standing variation. *Mol. Biol. Evol.* **29**, 1569–1585 (2012).

158. Z. Liu, P. K. Yadav, X. Xu, J. Su, C. Chen, M. Tang, H. Lin, J. Yu, J. Qian, P.-C. Yang, X. Wang, The increased expression of IL-23 in inflammatory bowel disease promotes intraepithelial and

lamina propria lymphocyte inflammatory responses and cytotoxicity. *J. Leukoc. Biol.* **89**, 597–606 (2011).

159. V. Gateva, J. K. Sandling, G. Hom, K. E. Taylor, S. A. Chung, X. Sun, W. Ortmann, R. Kosoy, R. C. Ferreira, G. Nordmark, I. Gunnarsson, E. Svenungsson, L. Padyukov, G. Sturfelt, A. Jönsen, A. A. Bengtsson, S. Rantapää-Dahlqvist, E. C. Baechler, E. E. Brown, G. S. Alarcón, J. C. Edberg, R. Ramsey-Goldman, G. McGwin, J. D. Reveille, L. M. Vilá, R. P. Kimberly, S. Manzi, M. A. Petri, A. Lee, P. K. Gregersen, M. F. Seldin, L. Rönnblom, L. A. Criswell, A.-C. Syvänen, T. W. Behrens, R. R. Graham, A large-scale replication study identifies TNIP1, PRDM1, JAZF1, UHRF1BP1 and IL10 as risk loci for systemic lupus erythematosus. *Nat. Genet.* **41**, 1228–1233 (2009).

160. H. Bouali, P. Nietert, T. M. Nowling, J. Pandey, M. A. Dooley, G. Cooper, J. Harley, D. L. Kamen, J. Oates, G. Gilkeson, Association of the G-463A myeloperoxidase gene polymorphism with renal disease in african americans with systemic lupus erythematosus. *J. Rheumatol.* **34**, 2028–2034 (2007).

161. Y. Li, W. Liao, M. Cargill, M. Chang, N. Matsunami, B.-J. Feng, A. Poon, K. P. Callis-Duffin, J. J. Catanese, A. M. Bowcock, M. F. Leppert, P.-Y. Kwok, G. G. Krueger, A. B. Begovich, Carriers of rare missense variants in IFIH1 are protected from psoriasis. *J. Invest. Dermatol.* **130**, 2768–2772 (2010).

162. E. Galimova, R. Rätsep, T. Traks, K. Kingo, V. Escott-Price, S. Kõks, Interleukin-10 family cytokines pathway: Genetic variants and psoriasis. *Br. J. Dermatol.* **176**, 1577–1587 (2017).

163. H. Tang, Z. Cheng, W. Ma, Y. Liu, Z. Tong, R. Sun, H. Liu, TLR10 and NFKBIA contributed to the risk of hip osteoarthritis: Systematic evaluation based on Han Chinese population. *Sci. Rep.* **8**, 10243 (2018).

164. M. S. Rajeevan, I. Dimulescu, J. Murray, V. R. Falkenberg, E. R. Unger, Pathway-focused genetic evaluation of immune and inflammation related genes with chronic fatigue syndrome. *Hum. Immunol.* **76**, 553–560 (2015).

165. P. R. Burton, D. G. Clayton, L. R. Cardon, N. Craddock, P. Deloukas, A. Duncanson, D. P. Kwiatkowski, M. I. Mc Carthy, W. H. Ouwehand, N. J. Samani, J. A. Todd, P. Donnelly, J. C. Barrett, P. R. Burton, D. Davison, P. Donnelly, J. C. Barrett, P. R. Burton, D. Davison, P. Donnelly, D. Easton, D. Evans, H.-T. Leung, J. L. Marchini, A. P. Morris, C. C. A. Spencer, M. D. Tobin, L. R. Cardon, D. G. Clayton, A. P. Attwood, J. P. Boorman, B. Cant, U. Everson, J. M. Hussey, J. D. Jolley, A. S. Knight, K. Koch, E. Meech, S. Nutland, C. V. Prowse, H. E. Stevens, N. C. Taylor, G. R. Walters, N. M. Walker, N. A. Watkins, T. Winzer, J. A. Todd, W. H. Ouwehand, R. W. Jones, Wendy L. Mc Ardle, S. M. Ring, D. P. Strachan, M. Pembrey, G. Breen, D. S. Clair, S. Caesar, K. Gordon-Smith, L. Jones, C. Fraser, E. K. Green, D. Grozeva, M. L. Hamshere, P. A. Holmans, I. R. Jones, G. Kirov, V. Moskvina, I. Nikolov, M. C. O'Donovan, M. J. Owen, N. Craddock, D. A. Collier, A. Elkin, A. Farmer, R. Williamson, P. M. Guffin, A. H. Young, I. N. Ferrier, S. G. Ball, A. J. Balmforth, J. H. Barrett, D. Timothy Bishop, M. M. Iles, A. Maqbool, N. Yuldasheva, A. S. Hall, P. S. Braund, P. R. Burton, R. J. Dixon, M. Mangino, S. Stevens, M. D. Tobin, J. R. Thompson, N. J. Samani, F. Bredin, M. Tremelling, M. Parkes, H. Drummond, C. W. Lees, E. R. Nimmo, J. Satsangi, S. A. Fisher, A. Forbes, C. M. Lewis, C. M. Onnie, N. J. Prescott, J. Sanderson, C. G. Mathew, J. Barbour, M. Khalid Mohiuddin, C. E. Todhunter, J. C. Mansfield, T. Ahmad, F. R. Cummings, D. P. Jewell, J. Webster, M. J. Brown, D. G. Clayton, G. Mark Lathrop, J. Connell, A. Dominiczak, N. J. Samani, Carolina A. Braga Marcano, B. Burke, R. Dobson, J. Gungadoo, K. L. Lee, P. B. Munroe, S. J. Newhouse, A. Onipinla, C. Wallace, M. Xue, M. Caulfield, M. Farrall, A. Barton, The Biologics in RA Genetics and Genomics, I. N. Bruce, H. Donovan, S. Eyre, P. D. Gilbert, S. L. Hider, A. M. Hinks, S. L. John, C. Potter, A. J. Silman, D. P. M. Symmons, W. Thomson, J. Worthington, D. G. Clayton, D. B. Dunger, S. Nutland, H. E. Stevens, N. M. Walker, B. Widmer, J. A. Todd, T. M. Frayling, R. M. Freathy, H. Lango, J. R. B. Perry, B. M. Shields, M. N. Weedon, A. T. Hattersley, G. A. Hitman, M. Walker, K. S. Elliott, C. J. Groves, C. M. Lindgren, N. W. Rayner, N. J. Timpson, E. Zeggini, Mark I. Mc Carthy, M. Newport, G. Sirugo, E. Lyons, F. Vannberg, A. V. S. Hill, L. A. Bradbury, C. Farrar, J. J. Pointon, P. Wordsworth, M. A. Brown, J. A. Franklyn, J. M. Heward, M. J. Simmonds, S. C. L. Gough, S. Seal, Breast Cancer Susceptibility Collaboration, M. R. Stratton, N. Rahman, M. Ban, A. Goris, S. J. Sawcer, A. Compston, D. Conway, M. Jallow, M. Newport, G. Sirugo, K. A. Rockett, D. P. Kwiatkowski, S. J. Bumpstead, A. Chaney, K. Downes, M. J. R. Ghori, R. Gwilliam, S. E. Hunt, M. Inouye, A. Keniry, E. King, R. M. Ginnis, S. Potter, R. Ravindrarajah, P. Whittaker, C. Widden, D. Withers, P. Deloukas,

(Wellcome Trust Sanger Institute Hinxton), H.-T. Leung, S. Nutland, H. E. Stevens, N. M. Walker, J. A. Todd, D. Easton, D. G. Clayton, P. R. Burton, M. D. Tobin, J. C. Barrett, D. Evans, A. P. Morris, L. R. Cardon, N. J. Cardin, D. Davison, T. Ferreira, J. Pereira-Gale, I. B. Hallgrimsdóttir, B. N. Howie, J. L. Marchini, C. C. A. Spencer, Z. Su, Y. Y. Teo, D. Vukcevic, P. Donnelly, D. Bentley, M. A. Brown, L. R. Cardon, M. Caulfield, D. G. Clayton, A. Compston, N. Craddock, P. Deloukas, P. Donnelly, M. Farrall, S. C. L. Gough, A. S. Hall, A. T. Hattersley, A. V. S. Hill, D. P. Kwiatkowski, C. G. Mathew, Mark I. Mc Carthy, W. H. Ouwehand, M. Parkes, M. Pembrey, N. Rahman, N. J. Samani, M. R. Stratton, J. A. Todd, J. Worthington,, Genome-wide association study of 14,000 cases of seven common diseases and 3,000 shared controls. *Nature* **447**, 661–678 (2007).

166. B. E. Hart, R. I. Tapping, Genetic Diversity of Toll-Like Receptors and Immunity to M. leprae Infection. *J. Trop. Med.* **2012**, 415057 (2012).

167. S. Walsh, F. Liu, K. N. Ballantyne, M. van Oven, O. Lao, M. Kayser, IrisPlex: A sensitive DNA tool for accurate prediction of blue and brown eye colour in the absence of ancestry information. *Forensic Sci. Int. Genet.* **5**, 170–180 (2011).

168. S. Walsh, F. Liu, A. Wollstein, L. Kovatsi, A. Ralf, A. Kosiniak-Kamysz, W. Branicki, M. Kayser, The HIrisPlex system for simultaneous prediction of hair and eye colour from DNA. *Forensic Sci. Int. Genet.* **7**, 98–115 (2013).

169. S. Walsh, L. Chaitanya, K. Breslin, C. Muralidharan, A. Bronikowska, E. Pospiech, J. Koller, L. Kovatsi, A. Wollstein, W. Branicki, F. Liu, M. Kayser, Global skin colour prediction from DNA. *Hum. Genet.* **136**, 847–863 (2017).

170. S. Walsh, L. Chaitanya, L. Clarisse, L. Wirken, J. Draus-Barini, L. Kovatsi, H. Maeda, T. Ishikawa, T. Sijen, P. De Knijff, W. Branicki, F. Liu, M. Kayser, Developmental validation of the HIrisPlex system: DNA-based eye and hair colour prediction for forensic and anthropological usage. *Forensic Sci. Int. Genet.* **9**, 150–161 (2014).
